# A core outcome set for evaluating the effectiveness of mixed-diagnosis falls prevention interventions for people with Multiple Sclerosis, Parkinson’s Disease and stroke

**DOI:** 10.1371/journal.pone.0294193

**Published:** 2023-11-13

**Authors:** Nicola O’Malley, Susan Coote, Fiona McCullough Staunton, Eileen O’Connor, Amanda M. Clifford

**Affiliations:** 1 School of Allied Health, Faculty of Education and Health Sciences, University of Limerick, Limerick, Ireland; 2 Ageing Research Centre, Health Research Institute, University of Limerick, Limerick, Ireland; 3 Centre of Physical Activity for Health, Health Research Institute, University of Limerick, Limerick, Ireland; 4 Multiple Sclerosis Society of Ireland, Dublin, Ireland; 5 Falls Research Public and Patient Involvement Panel, School of Allied Health, Faculty of Education and Health Sciences, University of Limerick, Limerick, Ireland; 6 Health Service Executive Mid-West Community Healthcare Organisation, Rehabilitation Unit, St. Joseph’s Hospital, Ennis, Clare, Ireland; The University of Sydney, AUSTRALIA

## Abstract

**Introduction:**

Clinical trials evaluating the effectiveness of falls prevention interventions for people with Multiple Sclerosis (MS), Parkinson’s Disease (PD) and stroke measure heterogeneous outcomes, often omitting those meaningful to patients. A core outcome set (COS) is a standardised set of outcomes that should be assessed in all trials within a research area. The aim of this study was to develop a COS for evaluating mixed-diagnosis falls prevention interventions for people with MS, PD and stroke in non-acute and community settings, with input from relevant stakeholder groups.

**Methods:**

Previously published research undertaken by the team, including a qualitative study with 20 patients and a review of the literature, were used to derive a longlist of potential outcomes. Outcomes were prioritised for inclusion in the COS using a three-round online Delphi survey. A multi-stakeholder, consensus meeting was conducted to agree upon the final COS and to provide a recommendation for a single outcome measure for each outcome in the COS.

**Results:**

Forty-eight participants were recruited across four stakeholder groups (researchers, patients, clinicians, and service-planners/policymakers). A total of 42 participants (87.5%) completed all three rounds of the surveys. Sixty-two outcomes were considered for inclusion in the COS throughout the Delphi process. A total of 15 participants attended the consensus meeting where they agreed upon the final COS and accompanying measurement instruments: fall incidence, injurious fall incidence, quality of life, falls self-efficacy, fear of falling, activity curtailment due to fear of falling, and cost-effectiveness. Attendees at the consensus meeting recommended that the proposed mechanism of impact of an intervention is considered when selecting additional outcomes outside of those in the COS to assess.

**Conclusions:**

This study identified a COS for evaluating the effectiveness of mixed-diagnosis falls prevention interventions for people with MS, PD and stroke. It is recommended that this COS and accompanying measurement instruments be used in all future trials in this research area so that findings can be combined and compared.

## Introduction

The extent of the problem of falls among people with neurological conditions has been well established in the literature, with research showing that more than half of people with MS and PD will fall within a three- or six-month period, respectively [1, 2], while nearly three quarters of stroke survivors fall within a year of the stroke occurring [3]. Fall events can have detrimental consequences for individuals, including physical injury, reduced psychosocial wellbeing and increased dependence [4–8]. In addition, the impact of falls at a societal level cannot be overlooked, with falls resulting in substantial healthcare utilisation and increased acute and long-term care needs [4, 5, 9].

Heterogeneity of outcomes evaluated and reported in falls prevention trials, in addition to substantial variation in the definition of these outcomes and the measures used to assess them, are consistently recognised as limitations in falls research for people with MS, PD and stroke [10–14]. This heterogeneity between trials hinders the synthesis and comparison of data and, consequently, certainty in the available evidence [10, 13, 14]. A proactive approach is therefore required to prevent these issues from transcending into future mixed-diagnosis falls research.

A core outcome set (COS) is a standardised set of outcomes that should be measured and reported at a minimum in all trials for a specific health construct or condition [15]. There are two main steps in the development of a COS, the first is to gain consensus on ‘what’ to measure and the second is to determine ‘how’ to define and measure the outcomes selected [15]. The development of a COS is increasingly recognised as a solution to the problem of heterogeneous outcomes [16, 17]. Additional benefits of a COS include a reduction in selective reporting of outcomes and increased relevance of outcomes to key stakeholders [16]. The advantages of a COS are evident in falls research for older adults where the establishment of a COS in 2005 has enabled the completion of large-scale meta-analyses that have informed the development of comprehensive evidence-based falls prevention guidelines for this cohort [18–21]. Consistent and relevant outcome reporting of mixed-diagnosis falls prevention trials for people with MS, PD and stroke would enable comparison and combination of data, thereby facilitating advancements in evidence-based practice.

The aim of this study was to develop a consensus-based core outcome set for evaluating the effectiveness of mixed-diagnosis falls prevention interventions for people with MS, PD and stroke. To achieve this, there were three specific objectives: (1) to generate a list of potential outcomes based on existing literature and through completing primary research, (2) to gain consensus on a list of core outcomes for inclusion in future studies, and (3) to provide recommendations for standardised definitions and measures for these core outcomes.

## Materials and methods

### Protocol and prospective registration

Detailed methodology for this study was prospectively registered with the COMET Initiative (https://comet-initiative.org/Studies/Details/1940) and published in an open access repository [22]. The research methods used in this study are based on the recommendations of the COMET Initiative [17] and the Core Outcome Set-STAndards for Development (COS-STAD) [23], and are reported in line with the Core Outcome Set-STAndards for Reporting (COS-STAR) Equator Network guidelines [24].

### Ethical considerations

Ethics approval for this study was granted by the Faculty of Education and Health Sciences Research Ethics Committee at the University of Limerick (EHSREC No: 2021_06_12). Participants were given a unique identifier at entry to this study and their personal information was only accessible to members of the research team. Electronic informed consent was obtained prior to participation in this study. At the beginning of round one of the surveys, participants read the participant information leaflet, research privacy notice and informed consent form. Once they had read these documents, participants could consent to taking part and having their pseudonymised data included in analyses by ticking the relevant box. Participants were only able to access the survey once they had consented to taking part and were advised that they could withdraw from this study at any time without explanation.

### Design

**Fig 1:**
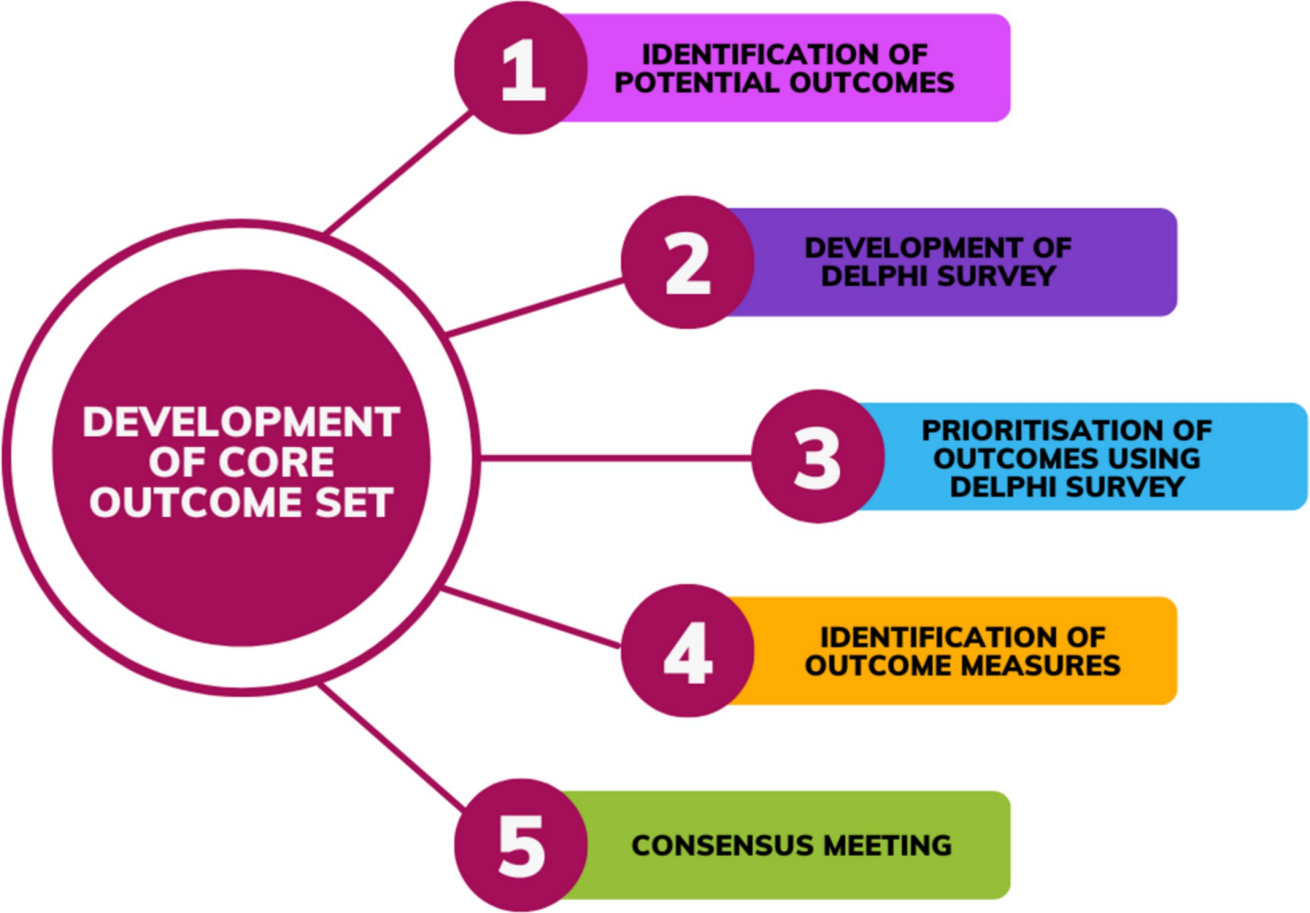
Flowchart of the main stages in the core outcome set development process.

This was an international, multi-perspective Delphi consensus study, with five key stages as presented in Fig 1 and described in further detail below.

#### Stage 1: Identifying potential outcomes

A comprehensive list of outcomes related to falls prevention for people with MS, PD and stroke was derived from multiple sources. Firstly, outcomes used in existing literature were extracted from an umbrella review of systematic reviews investigating the effectiveness of falls prevention interventions for people with MS, PD and stroke [11]. A systematic literature search using 15 electronic databases, grey literature searches and hand-screening of reference lists was undertaken to identify potentially relevant systematic reviews. Quantitative systematic reviews, mixed-methods systematic reviews (quantitative components only) or pooled analyses investigating the effectiveness of non-surgical or non-pharmacological falls prevention interventions were included in the umbrella review. Further details regarding the search strategy, inclusion and exclusion criteria, and data extraction are reported elsewhere [11, 25]. This umbrella review included 18 systematic reviews, representing 73 unique primary studies. The outcomes reported, their definitions, the outcome measures used and the time points for measurement were recorded for each systematic review. This list was complemented with additional outcomes that are not typically assessed in trials but were considered meaningful and important to people with MS, PD and stroke. These outcomes were identified through a qualitative study undertaken among people living with MS, PD and stroke [26]. A total of 20 individuals participated in online focus groups or semi-structured telephone interviews. Audio-recorded data were transcribed verbatim and analysed through the six phases of reflexive thematic analysis, using a predominantly inductive approach [27–29]. Outcomes that are desirable to participants were identified through this qualitative study and were combined with those reported in the systematic reviews included in the umbrella review. Any outcomes suggested by participants that were not assessed in the systematic reviews were added to the longlist of potential outcomes for the Delphi surveys. The list was further supplemented by discussions with the members of the public and patient involvement (PPI) panel. Finally, respondents to the subsequent Delphi survey had the opportunity to propose additional outcomes at the end of round one.

#### Stage 2: Development of the Delphi survey

The lists of outcomes generated from each of the above sources were reviewed and discussed by members of the research team and the PPI panel to identify all distinct outcomes to be included in the Delphi survey rounds for consideration as a core outcome. The online software Qualtrics (Provo, UT) was used to design and administer the three Delphi survey rounds. All outcomes identified through the first stage of this study were presented in alphabetical order in the survey to avoid potential weighting [30]. The survey was developed with input from the PPI panel to promote ease of completion. Following its development, the survey was pilot tested on researchers (n = 2), patients (n = 2) and clinicians (n = 2) to assess for clarity and was modified accordingly prior to formal circulation to participants.

Delphi survey respondents were asked to score each outcome using the Grading of Recommendations Assessment, Development and Evaluations (GRADE) nine-point Likert scale, where a score of 1–3 signifies that an outcome is of limited importance, 4–6 indicates an outcome that is important but not critical, and 7–9 denotes a critically important outcome [15, 31]. This study used the ‘70/15%’ consensus definition, with consensus on inclusion defined as 70% or more of the participants rating it is critically important (7–9) and less than 15% rating it as being of limited importance (1–3), while consensus that an outcome should be excluded from the COS was defined as 70% or more of the participants rating is as being of limited importance (1–3) and less than 15% rating it as critically important (7–9) [15]. Any score distribution outside of those described above demonstrated a lack of consensus with respect to the inclusion or exclusion of the outcome in the COS [15].

#### Stage 3: Prioritisation of outcomes

The consensus process involved a three-round online Delphi process. In round one, participants were asked to rate the importance of each outcome using the GRADE scale outlined above and were also encouraged to provide the rationale for each of their scores. At the end of the survey, participants had a free-text option to suggest additional outcomes for inclusion in the next round of the surveys. The outcomes suggested were reviewed by members of the research team to determine if they were unique outcomes [30]. All outcomes from round one were brought forward to round two to allow participants to reflect on the scores and feedback of each participant group before deciding whether or not they wished to change their score.

In round two of the Delphi process, participants were provided with their own score, the overall ratings for the overall group and for each stakeholder group, and a summary of the reasons that participants gave for their scoring of each outcome. Participants were asked to reflect on this information and then rescore all outcomes again using the GRADE scale. Following the completion of round two of the Delphi process, outcomes were separated into three categories: category A (outcomes reaching the criteria for consensus on inclusion), category B (outcomes not reaching consensus) or category C (outcomes reaching the criteria for consensus on exclusion). Outcomes in category A were added to the preliminary COS, outcomes in category B were added to the list of ‘supplementary outcomes’ and were considered for inclusion in a third round of the Delphi process, and outcomes in category C were excluded from any further consideration for the COS.

In line with the predefined criteria described in the study protocol, a third round of the Delphi surveys was completed for any outcomes in category B that were rated as critically important by more than 50% of participants and rated as having limited importance by less than 15% in round two. As per previous rounds, participants were provided with their own score, the overall ratings for the overall group and for each stakeholder group, and a summary of the reasons that participants gave for their scoring of each outcome before being asked to rescore the outcomes. Following round three, the scores for each outcome were analysed to determine if they now met the criteria for category A or if they were to remain in category B.

Following a research team meeting where the very high number of outcomes in category B after three rounds of the surveys was noted and discussed, it was decided by team members that the outcomes in category B should be further subdivided to allow for adequate time to discuss potential outcomes for the final COS during the consensus meeting. Any outcome on this list that >50% of participants had rated as critically important and <15% of participants had rated as being of limited importance were added to category B1 and were carried forward for further discussion at the meeting as they were nearing the predefined threshold for inclusion. Outcomes in category B that <50% of participants had rated as critically important or >15% had rated as being of limited importance were added to category B2 and were excluded from any further consideration for the final COS as it was decided that they had not been sufficiently prioritised by the overall group throughout the Delphi process to warrant inclusion. Outcomes in category B2 remained on the supplementary outcomes list from this point onwards.

#### Stage 4: Identification of outcome measures and definitions

This stage of the project was centred around the selection of a single instrument for measuring each core outcome, to ensure consistency and comparability across all future trials. A pragmatic approach was taken to this process with outcome measures and definitions extracted from the umbrella review or else identified through targeted literature searches. Our research team and stakeholder group reviewed the available evidence and provisionally recommended the use of a single outcome measure after consideration and discussion of the following: the frequency with which the measure has been used and its responsiveness in existing studies, the time and resources required to use the measure, and the available data on their psychometric properties as outlined in the COnsensus-based Standards for the selection of health status Measurement INstruments (COSMIN) recommendations (validity, reliability, responsiveness and interpretability) [32, 33].

#### Stage 5: Consensus meeting

A virtual face-to-face consensus meeting was held using the Zoom platform on the 5^th^ of January 2023. This online interactive meeting took place with representatives from each stakeholder group to discuss, vote and gain consensus on the final COS, in addition to the definitions and measures to be used to assess these outcomes. At the end of second Delphi survey, all participants were asked to select if they would like to take part in the consensus meeting. A poll was sent around to those who responded yes to determine their availability to attend at different dates and times. The meeting took place on the date that suited the majority of participants.

The meeting commenced with a presentation describing the preliminary COS and the category B1 outcomes that were near the inclusion criteria. The ratings from the overall group and from each stakeholder group were presented for each outcome on these lists. This was followed by a discussion between attendees regarding the reasons for including or excluding each outcome from the COS and a final vote. The definition for consensus was a minimum of 70% of participants and at least one patient participant voting for the outcome to be included in the COS [34]. Any outcomes not meeting these criteria were added to the supplementary outcomes list accompanying this COS. For each outcome achieving consensus for inclusion in the final COS, recommendations regarding outcome measures and definitions were discussed. Attendees were encouraged to provide feedback on the recommendations and/or to suggest alternatives for discussion. Following this, a final vote on the selection of a single outcome measure and definition, where applicable, took place. Once again, the definition for consensus was a minimum of 70% of participants and at least one patient participant voting for the outcome measure/definition to be recommended alongside the COS.

### Participants

Participants were eligible if they were aged 18 years or over, able to read and write in English, and were either (a) living with a confirmed diagnosis of MS, PD and/or stroke, (b) researchers with a minimum of three peer-reviewed papers in the research field, (c) clinicians providing interventions to people with MS, PD and stroke, or (d) service-planners/policymakers for falls prevention services.

Participants were recruited to this study from May 2022 to September 2022. Patient participants were recruited through support groups/community services, in addition to social media and other communications of relevant organisations. Participants across the other three stakeholder groups (researchers, clinicians, and service-planners/policymakers) were recruited via email identified from relevant research papers, professional body email lists, special interest groups and social media.

### Data analysis

The Delphi survey responses were analysed using SPSSv28. For all outcomes, descriptive statistics were used to show the scores from the overall group and each individual stakeholder group for the three GRADE categories to determine if they met the predefined inclusion or exclusion criteria. A conventional approach to content analysis was used to analyse all qualitative data collated from open comments [35]. Participants who identified as being a representative of more than one of the stakeholder groups in this study were asked to state in what primary capacity they were completing the study and were analysed as part of this stakeholder group to prevent double counting of data. Attrition bias was assessed by comparing the distribution of median scores across the outcomes for those not participating in rounds two or three with those who did [36].

### Public and patient involvement

**Fig 2:**
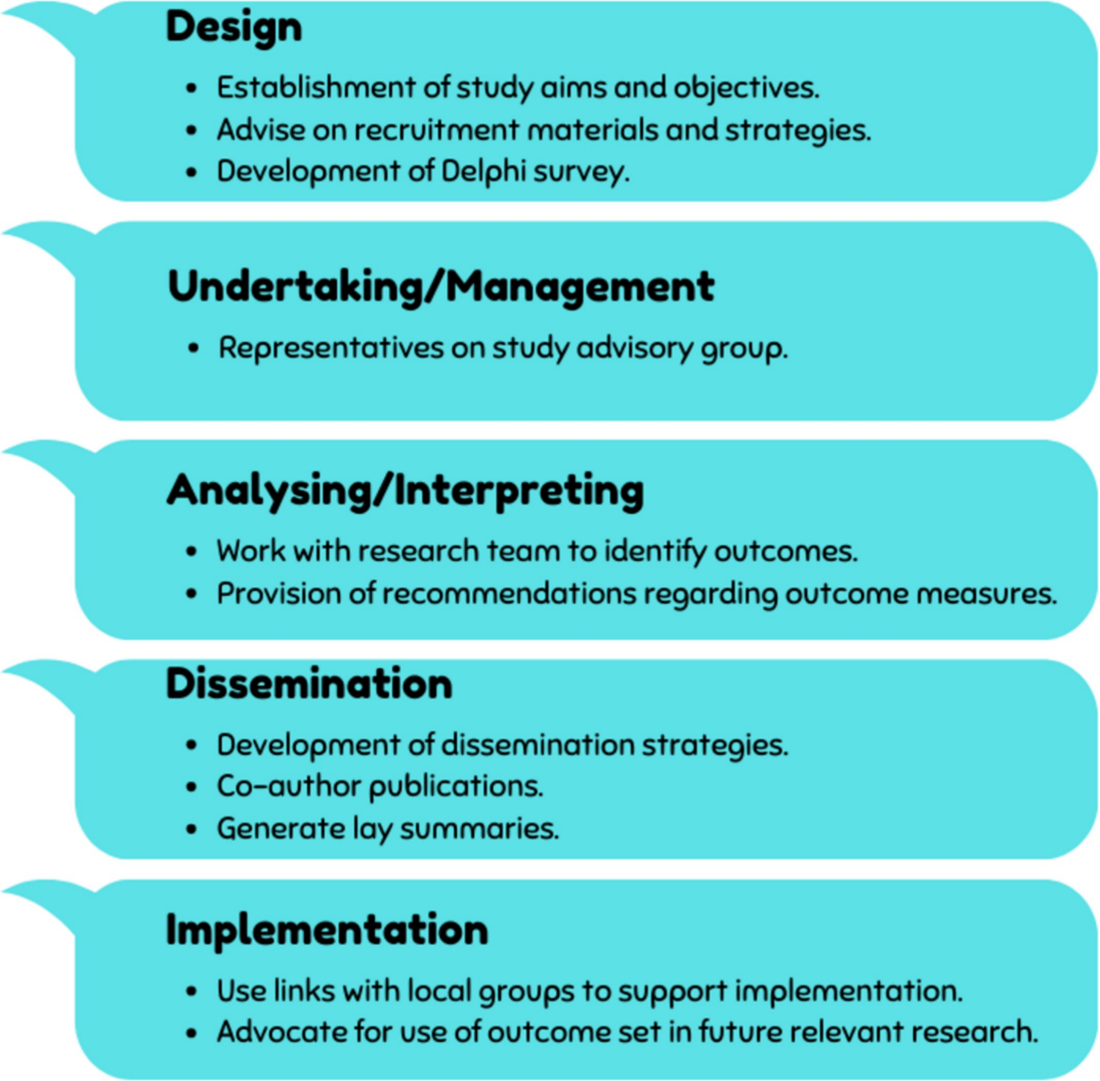
Overview of public and patient involvement in the study.

The importance and benefits of including patients and other relevant stakeholders in the design and oversight of COS studies as ‘public research partners’ has been highlighted by the COMET Initiative [17]. Consequently, a PPI panel was established to guide the development of this study. This group included relevant stakeholders including individuals living with MS, PD and stroke, healthcare professionals, and representatives working with patient organisations. As shown in Fig 2, this PPI panel had roles in the design stage through to the dissemination and implementation stages of this study.

### Deviations from protocol

Given the high number of outcomes in category B following three rounds of the surveys, this category had to be subdivided so that not all outcomes in this category were brought forward for consideration during the consensus meeting, as originally planned, to allow for sufficient time to discuss potential outcomes for the final COS and appropriate outcome measures.

## Results

### Identification of outcomes

Our review of the literature [11] led to the identification of 39 individual outcomes. A further 13 unique outcomes were proposed by people with MS, PD and stroke during our qualitative study [26], while six additional outcomes were suggested by members of the PPI panel. Finally, four new outcomes were submitted by participants at the end of round one of the Delphi surveys. Consequently, a total of 62 outcomes were considered for inclusion in this COS. The full list of outcomes is presented in S1 Appendix.

### Delphi process

**Fig 3:**
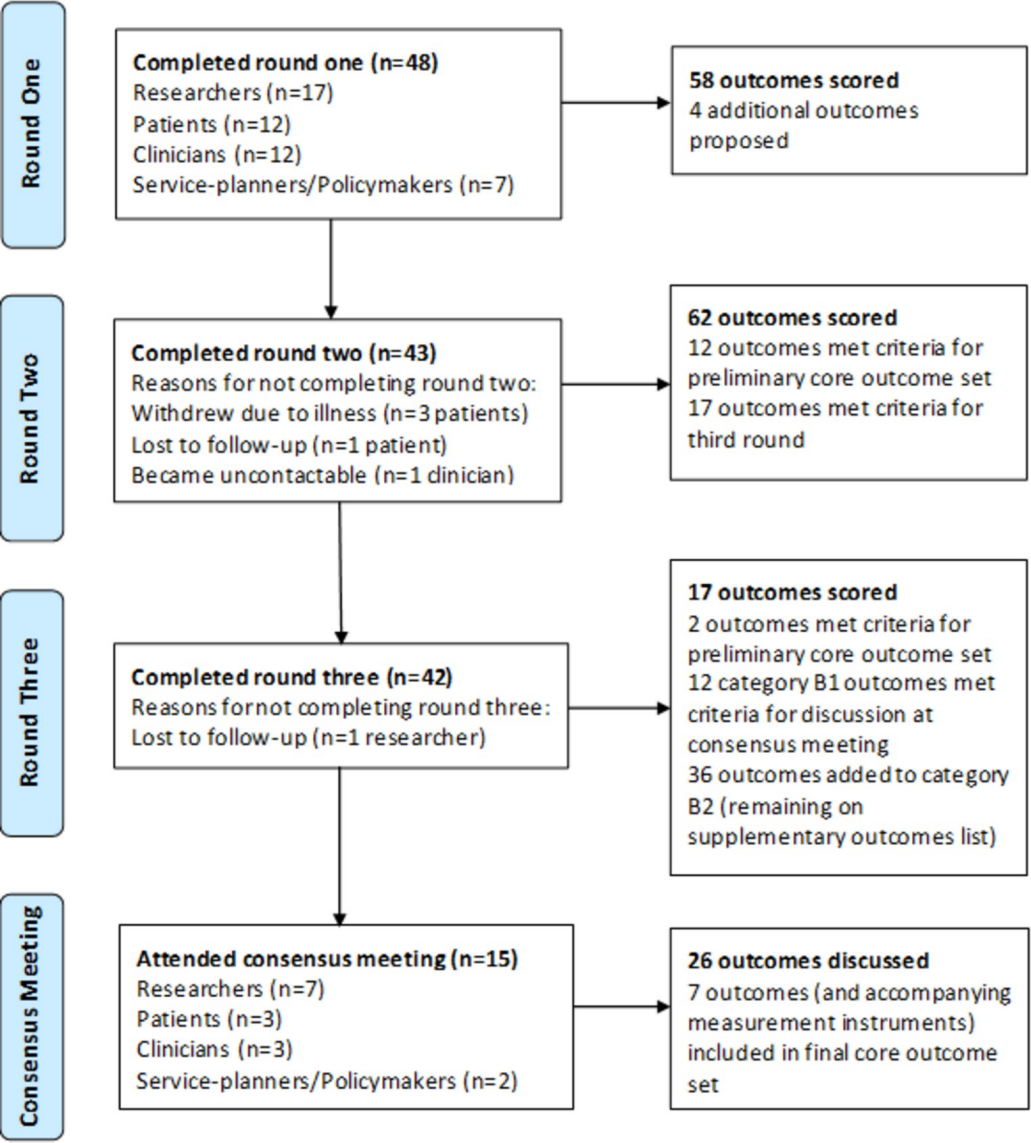
Flow of participants throughout the Delphi process.

A total of 48 participants from 11 countries registered to participate in this study across the four stakeholder groups. When divided based on primary capacity for completing the survey, n = 12 were included in the patient participant group, n = 17 in the researcher group, n = 12 in the clinician group, and n = 7 in the service-planner/policymaker group. Demographic characteristics of participants are presented in Table 1. The flow of participants throughout the Delphi consensus process is summarised in Fig 3. A total of 42 participants (87.5%) completed all three rounds of the surveys. Participants who did not complete round two of the Delphi survey had comparable median scores in round one (with overlap in interquartile ranges) when compared with the scores of participants who completed both surveys. Also, the second-round scores of the participant lost to follow-up in round three were comparable to the median scores of those who completed all three survey rounds (S2 Appendix).

**Table 1:** Characteristics of the Delphi survey participants.

	Round 1 (n = 48)	Round 2 (n = 43)	Round 3 (n = 42)
**Relevant stakeholder group *n* (%)** (Some listed more than one group)			
Researcher	17 (35%)	17 (40%)	16 (38%)
Patient	13 (27%)	9 (21%)	9 (21%)
Clinician	24 (50%)	23 (53%)	23 (55%)
Service-planner/ Policymaker	8 (17%)	8 (19%)	8 (19%)
**Primary capacity for completing study *n* (%)**			
Researcher	17 (35%)	17 (39%)	16 (38%)
Patient	12 (25%)	8 (19%)	8 (19%)
Clinician	12 (25%)	11 (26%)	11 (26%)
Service-planner/ Policymaker	7 (15%)	7 (16%)	7 (17%)
**Gender *n* (%)**			
Female	33 (69%)	30 (70%)	30 (71%)
Male	15 (31%)	13 (30%)	12 (29%)
**Country *n* (%)**			
Ireland	23 (48%)	20 (47%)	20 (48%)
England	7 (15%)	6 (14%)	6 (14%)
Australia	6 (13%)	6 (14%)	6 (14%)
USA	3 (6%)	3 (7%)	2 (5%)
Canada	3 (6%)	3 (7%)	3 (7%)
Italy	1 (2%)	1 (2.2%)	1 (2.4%)
Japan	1 (2%)	1 (2.2%)	1 (2.4%)
Singapore	1 (2%)	1 (2.2%)	1 (2.4%)
Belgium	1 (2%)	1 (2.2%)	1 (2.4%)
France	1 (2%)	0 (0%)	0 (0%)
Germany	1 (2%)	1 (2.2%)	1 (2.4%)
**Age (years)**			
Mean (standard deviation)	48.79 (11.83)	48.07 (10.41)	48.17 (10.51)
Range	25–86	25–69	25–69

The results of each round of the Delphi surveys are presented in detail in S3–S5 Appendices and the categorisation of outcomes following completion of the survey rounds is summarised in S6 Appendix. Following the Delphi process, a total of 26 outcomes were brought forward for discussion at the consensus meeting; 14 category A outcomes that met the pre-defined criteria for inclusion in the preliminary COS, and 12 category B1 outcomes identified as nearing the threshold for inclusion. All other outcomes were in category B2, remaining on the supplementary outcomes list and were not given further consideration for inclusion in the final COS.

### Consensus meeting

The consensus meeting was attended by a panel of 15: n = 3 patient participants, n = 7 researchers, n = 3 clinicians, and n = 2 service-planners/policymakers. Demographic characteristics of the individuals who attended the consensus meeting are summarised in Table 2. A summary of the final COS is presented in S7 Appendix. Each recommendation made by the attendees at the consensus meeting, followed by a summary of the key discussion points that informed the recommendation, is described in further detail below.

**Table 2:** Characteristics of the participants in attendance at the consensus meeting.

**Relevant stakeholder group *n* (%)** (Some listed more than one group)	
Researcher	7 (47%)
Patient	3 (20%)
Clinician	7 (47%)
Service-planner/ Policymaker	3 (20%)
**Primary capacity for completing study *n* (%)**	
Researcher	7 (47%)
Patient	3 (20%)
Clinician	3 (20%)
Service-planner/ Policymaker	2 (13%)
**Gender *n* (%)**	
Female	12 (80%)
Male	3 (20%)
**Country *n* (%)**	
Ireland	10 (66%)
England	1 (7%)
USA	1 (7%)
Canada	2 (13%)
Italy	1 (7%)

### Fall incidence

#### Recommendation

Fall incidence should be assessed in all future mixed-diagnosis falls prevention interventions for people with MS, PD and stroke. This outcome should be reported as falls per person per year. Fall events should be prospectively recorded daily with follow-up by team members at least monthly to reduce recall bias. A fall event should be defined as ‘an unexpected event in which the participant comes to rest on the ground, floor, or lower level’ [18].

#### Key discussion points

Falls rate and total number of falls were prioritised as core outcomes in the Delphi survey rounds. Due to the degree of overlap between these outcomes and subsequent outcome measures, a recommendation was made to the attendees at the consensus meeting to combine these outcomes into one outcome, fall incidence. No counterarguments were made to this proposal and all attendees voted for fall incidence to be included in the COS. Our research team proposed the use of falls per person per year to report this outcome as it allows for comparison of studies with different reporting periods [37]. Further, given the issue of under-reporting of fall events associated with the retrospective recording of falls [38, 39], we suggested that falls be prospectively recorded daily with follow-up by team members at least monthly to reduce recall bias [37]. Potential challenges with the monthly follow-up by the research team, particularly with respect to large intervention studies with high numbers of participants were noted. A counter argument proposed that follow-up with participants affords the research team the opportunity to clarify what happened and determine whether it meets the definition for a fall, thereby improving the accuracy of findings.

The second part of the discussion focused on the definition of a fall. From our review of the literature, we identified that the most commonly used definition is ‘an unexpected event in which the participant comes to rest on the ground, floor or lower level’ [18]. However, from our qualitative research we identified discrepancies between that definition and what patients consider a fall event, namely that patients consider a fall event a ‘loss of balance’ and believe that instances where an external support or individual prevents them from ending up on the ground should be considered a fall event. In light of these findings, our research team proposed making amendments to the definition of a fall that were reflective of these views. Following an extensive discussion, consensus from attendees was that the original definition should be included with the COS as extra elements opens the definition to interpretation by participants and so could compromise the accuracy of the outcomes. Participants stated that instances where an individual has a voluntary reaction to reach out and grab a support demonstrates a positive reactive falls prevention strategy and so should not be considered a fall event. It was also suggested that amendments could lead to the overreporting of fall events in circumstances where an overprotective person caught an individual to prevent a fall despite the individual having the balance reactions to prevent the fall themselves. Finally, participants felt that the amendments to the definition would describe near-fall events rather than actual fall events.

### Injurious fall incidence

#### Recommendation

Injurious fall incidence should be assessed in all future mixed-diagnosis falls prevention interventions for people with MS, PD and stroke. This outcome should be reported as injurious falls per person per year. Injurious falls should be prospectively recorded daily with follow-up by team members at least monthly to reduce recall bias. The standardised system for categorising and defining fall related injuries proposed by Schwenk et al. (2012) should be used (Table 3) [40]. Each category should be reported even if a study is not powered to detect effects as this will enable future meta-analyses.

**Table 3:** Standardised system for categorising and defining fall-related injuries.

Category	Definition
A–serious injury	Medically recorded fracture, head or internal injury requiring accident and emergency or inpatient treatment
B–moderate injury	Wounds, bruises, sprains, cuts requiring a medical/health professional examination such as physical examination, x-ray or suture
C–minor injury	Minor bruises or abrasions not requiring health professional assistance; reduction in physical function (e.g., due to pain, fear of falling) for at least three days
D–no injury	No physical injury detected

Schwenk M, Lauenroth A, Stock C, Moreno RR, Oster P, McHugh G, et al. Definitions and methods of measuring and reporting on injurious falls in randomised controlled fall prevention trials: a systematic review. BMC Med Res Methodol. 2012;12:50.

#### Key discussion points

Due to degree of overlap between the outcomes and outcomes measures, a recommendation was made to combine number of injurious falls, number of falls resulting in healthcare utilisation and number of fall-related fractures into one outcome, injurious fall incidence. All attendees agreed that injurious fall incidence should be included in the final COS.

A systematic review undertaken by Schwenk et al. (2012) highlighted the substantial variation in the definitions and methods used to measure and document injurious falls [40]. Following this, they developed a standardised system for categorising and defining fall-related injuries [40]. Our research team proposed the use of this standardised system to measure injurious fall incidence as it captures the data of the three outcomes that were combined to generate this outcome. Some concerns over the accuracy of measures to assess injurious falls were expressed, with participants describing whether or not someone will seek medical attention for an injurious fall can be very subjective and system-dependent. A patient participant also described how if an individual who does not typically fall suddenly experiences a fall event, they may seek medical attention not necessarily because they are injured but out of concern that their condition is progressing or that they need to change treatment. Following a discussion, participants ultimately agreed that the use of a standardised system was necessary to compare fall-related injuries and voted to include this measure.

### Quality of life

#### Recommendation

Quality of life should be assessed in all future mixed-diagnosis falls prevention interventions for people with MS, PD and stroke. This outcome should be measured using the 5-Level EuroQoL-5 (EQ-5D-5L) [41].

#### Key discussion points

During the discussion on whether to include quality of life in the COS, it was suggested that only falls outcomes should be included in the final COS since they align with the primary aim of the intervention, the prevention of falls. In response, other participants stated that the prevention of falls should not be the sole aim of an intervention. They described how fall events could be reduced by preventing an individual from completing all activities but did not believe that this should be considered a successful intervention. Rather, they stated that the goal of the intervention should be to improve quality of life by enabling individuals to complete their desired activities in a safer manner. It was also suggested that, given the importance placed upon quality of life by patient participants, individuals would not adhere to an intervention that was not improving this outcome. Following this discussion, all attendees voted to include quality of life in the final COS.

Our research team proposed the use of the EQ-5D-5L to measure quality of life as it has been shown to be valid and reliable across multiple conditions and settings, it is available in more than 130 languages, can be used in cost-effectiveness analysis and requires minimal time to complete [42, 43]. One clinician stated that the measure can be slightly more time consuming in practice to interpret correctly if you are not familiar with the measure. No other arguments for or against its use were made and during the final vote the EQ-5D-5L reached consensus for inclusion.

### Falls self-efficacy

#### Recommendation

Falls self-efficacy should be assessed in all future mixed-diagnosis falls prevention interventions for people with MS, PD and stroke. This outcome should be measured using the Falls Efficacy Scale-International (FES-I) [44].

#### Key discussion points

While discussing whether to include falls self-efficacy in the final COS, one participant suggested combining falls self-efficacy, balance confidence and fear of falling into one outcome or else only including one of them in the COS. Other attendees disagreed with this proposal, asserting that the three outcomes are different constructs and as such should be kept separate. Following the final vote, falls self-efficacy was included in the COS.

Our research team proposed the use of the FES-I to assess falls self-efficacy as it has shown to be valid and reliable across multiple conditions, has been translated and tested in multiple languages, is available for free, and requires no equipment and minimal time [45–50]. Participants agreed that this is the best available measure for assessing falls self-efficacy and voted to include it.

### Fear of falling

#### Recommendation

Fear of falling should be assessed in all future mixed-diagnosis falls prevention interventions for people with MS, PD and stroke. This outcome should be assessed using the direct question ‘Are you afraid of falling?’ and five-point Likert scale: not at all, slightly, moderately, very or extremely.

#### Key discussion points

No counterarguments were made to the inclusion of fear of falling and during the final vote it reached consensus for inclusion in the COS. In our umbrella review, FES-I was the most commonly reported measure for assessing fear of falling, however, evidence has shown that falls self-efficacy and fear of falling are two distinct constructs [7, 51]. In the absence of a validated outcome measure, our research team proposed the use of a direct question and five-point Likert scale to measure fear of falling. Participants described benefits of the Likert scale including that it requires minimal time and is easy to administer. One clinician proposed changing the wording of the question from ‘are you afraid of falling?’ to ‘are you concerned about falling?’ as the terminology of the question may feed into the fear. However, a counterproposal was put forward that if we are measuring fear directly that it should be in the question to ensure we are capturing it accurately and not crossing over into other constructs such as falls self-efficacy. Following the vote, the direct question and five-point Likert scale reached consensus for inclusion.

### Activity curtailment due to fear of falling

#### Recommendation

Activity curtailment due to fear of falling should be assessed in all future mixed-diagnosis falls prevention interventions for people with MS, PD and stroke. This outcome should be assessed using the direct question ‘Do you think that fear of falling has made you cut down on any activities that you used to do?’ with the following response options: yes, no, don’t know or refused.

#### Key discussion points

All participants voted to include activity curtailment due to fear of falling in the COS. Our research team proposed the use of the Fear of Falling Avoidance Behaviour Questionnaire (FFABQ) as it has been validated for older adults with PD and stroke, no equipment is required, takes minimal time to complete and has high clinical feasibility [52]. A participant suggested the use of a single-item direct question for activity curtailment that is used in falls research for older adults: ‘Do you think that fear of falling has made you cut down on any activities that you used to do?’ [53, 54]. Participants were in favour of this single-item question and voted to include it in the final COS.

### Cost-effectiveness

#### Recommendation

Cost-effectiveness should be assessed in all future mixed-diagnosis falls prevention interventions for people with MS, PD and stroke. Researchers should consult with a health economist to determine the most appropriate way to calculate cost-effectiveness of an intervention.

#### Key discussion points

Cost-effectiveness was among the category B1 outcomes that had not met the criteria for inclusion in the preliminary COS but were brought forward for consideration at the consensus meeting as they were nearing the consensus threshold (i.e., >50% voted as being critically important and <15% voted as being of limited importance). When discussion on these category B1 outcomes was invited, participants proposed the inclusion of cost-effectiveness in the final COS despite not reaching consensus for priority in the survey rounds. The group recognised the challenges of doing a cost-effectiveness analysis, especially in terms of international standardisation, but felt it was important to include the outcome in the COS to optimise translation of interventions to practice and to promote adoption for funding by healthcare authorities. Participants also suggested that where similar interventions demonstrated similar effects, a cost-effectiveness analysis could be the factor to determine which to implement. Consequently, participants voted to include cost-effectiveness in the final COS. No recommendation regarding what measure to use was made by the group, instead, they recommended consulting a health economist to inform the decision.

### Mechanism of impact informing choice of additional outcomes

#### Recommendation

The anticipated mechanism of impact of an intervention should be considered and articulated when additional outcomes are assessed and reported.

#### Key discussion points

While they had been prioritised for inclusion in the COS during the Delphi survey rounds, the remaining five outcomes on the preliminary COS (dynamic balance, balance confidence, objectively assessed mobility, understanding of personal falls risk factors, and falls self-management skills) did not reach consensus for inclusion in the final COS. Following an interactive discussion between attendees, it was agreed that while these outcomes are important, their selection should be dependent on a number of factors such as the proposed mechanism of impact of the intervention, the aim of the intervention, and participant characteristics (e.g., stage of disease or level of mobility). Therefore, participants concluded that as they are only relevant to specific interventions, e.g., dynamic balance is only applicable to interventions aiming to reduce falls through improving balance, they should not be included as a core outcome for all falls prevention interventions.

### Category B1 outcomes

#### Recommendation

Ability to independently perform activities of daily living, falls risk, level of physical activity, lower limb strength, number of fallers, number of falls resulting in a long lie, number of recurrent fallers, objectively assessed ability to perform activities of daily living, self-efficacy, static balance, and time to first post-intervention fall should remain on the supplementary outcomes list.

#### Key discussion points

Those in attendance at the consensus meeting agreed that none of the remaining outcomes in category B1 (ability to independently perform activities of daily living, falls risk, level of physical activity, lower limb strength, number of fallers, number of falls resulting in a long lie, number of recurrent fallers, objectively assessed ability to perform activities of daily living, self-efficacy, static balance, and time to first post-intervention fall) were important enough to be included in the final COS. Consequently, these 11 outcomes remained on the supplementary outcomes list.

## Discussion

In this study, utilising formal consensus methods recommended by the COMET Initiative, we developed a COS of seven outcomes for mixed-diagnosis falls prevention interventions for people with MS, PD and stroke–fall incidence, injurious fall incidence, quality of life, falls self-efficacy, fear of falling, activity curtailment due to fear of falling, and cost-effectiveness. A single measurement instrument for assessing each of these outcomes was recommended. Consideration of the proposed mechanism of impact of an intervention when selecting additional outcomes was also recommended by the panel. This work was informed by an umbrella review, a qualitative study involving 20 people with MS, PD and stroke, a multi-perspective three-stage online Delphi survey that was completed by 48 participants from 11 countries, and an international multi-stakeholder consensus meeting. This COS should be used in future studies, reviews and guidelines on falls prevention for people with these neurological conditions.

A large number of outcomes were included for consideration across this Delphi process, and this is reflective of the broad nature of falls and falls risk among people with MS, PD and stroke. This COS does not preclude other outcomes from being assessed, but rather represents the minimum that should be measured [17]. Many outcomes that were not included in the final COS were still considered very important outcomes by participants depending on the type of intervention involved. Consequently, a key discussion during the consensus meeting was the need to consider the proposed mechanism of impact of the intervention when deciding what outcomes to assess besides those outlined in the COS. This recommendation aligns with the position set in the Medical Research Council (MRC) framework for the development and evaluation of complex interventions which asserts that uncovering the implicit theoretical basis of an intervention is crucial because it supports the identification of mechanisms of change and, therefore, relevant outcomes [55, 56].

Besides fall incidence, the majority of outcomes included in the final COS are currently infrequently evaluated in relevant research studies [11]. Moreover, despite reaching the highest level of agreement across the overall group (91%) for inclusion in the final COS during the Delphi surveys, activity curtailment due to fear of falling was not assessed in any of the papers in our umbrella review. To date, the only COS evaluating falls prevention interventions is that designed by Lamb et al. (2005) for the evaluation of fall injury prevention trials among older adults [18]. However, key differences exist between their COS and the one developed through this study, most notably the inclusion of fear of falling and activity curtailment due to fear of falling, outcomes reported as highly important by patients in our qualitative study, in addition to the inclusion of cost-effectiveness. The identification and inclusion of additional outcomes beyond those typically used in existing studies highlights the importance of involving relevant stakeholders in the consensus process to ensure that the selected outcomes are meaningful and relevant in future research.

A key objective of this project was to recommend the use of a single measurement instrument for each core outcome, to facilitate consistency and comparability across future trials. Although consensus was achieved on the inclusion of a measurement instrument for all outcomes except cost-effectiveness, evidence of the validity and information on the psychometric properties to support strong recommendations were often lacking. In the absence of such supporting data, other factors such as the frequency of use of the measure in existing studies, in addition to the time and resources required to administer it, were also taken into consideration, with participants often favouring the selection of a simple and pragmatic outcome measure. While the selection of measurement instruments that do not require excessive resource commitment may enhance implementation of the COS, further research is required to determine if they are the most appropriate and suitable outcome measures. This is especially true for the measurement instruments for fear of falling and activity curtailment due to fear of falling which have not been evaluated psychometrically. Given the small number of response options for the activity curtailment outcome measure, and that a relative degree of activity curtailment may be a protective mechanism for some individuals [57], exploration of the responsiveness of this suggested measurement instrument should be a priority. Also of note, despite being prioritised as critically important during the survey rounds, we were unable to identify any outcome measure for understanding of personal falls risk factors or falls self-management skills. Consequently, future research should focus on the development and psychometric evaluation of outcome measures for outcomes in the COS, as well as those prioritised during the survey rounds. As new evidence and outcome measures emerge, updates will be required to the existing recommendations in this COS.

Falls research for people with MS, PD and stroke is relatively in its infancy but is a rapidly growing research field. It is anticipated that as this important topic area continues to develop, further intervention types and approaches will be designed and evaluated. While the consensus process employed in this study ultimately resulted in the development of a COS with seven key outcomes, it is possible that the relevance and importance of outcomes in this field may evolve as research progresses. COMET describe how periodic review of a COS can enable developers to confirm its ongoing validity, ensure that included outcomes remain important and relevant, and to determine if additional outcomes should be added [17]. However, specific guidance on the optimal frequency with which to complete such a review is not currently available. In instances where periodic reviews were planned in advance, COS developers typically proposed timeframes ranging from three to five years [17]. Therefore, it is recommended that a review of this COS would take place in four years to ensure that it reflects any advancements in the research field and to decide whether an update of the COS is required.

### Strengths and limitations

In conducting this study, a number of strengths and limitations are acknowledged. Particular strengths of this study include the use of methods in line with the COMET [17] and COS-STAD recommendations [23], published in an a priori protocol [22]. Additionally, the involvement of the PPI panel throughout all stages of this study can be viewed as a major strength. Another strength is that the identification of potential outcomes was not restricted to the findings of the literature review. This is important as outcomes collated through literature reviews most often include outcomes that are important to researchers, potentially overlooking outcomes that patients consider important [58]. Our research team undertook primary qualitative research to capture the perspectives of people living with MS, PD and stroke. The value of this approach is evident through the inclusion of activity curtailment due to fear of falling, an outcome identified through our qualitative study, in the final COS. Moreover, this study engaged multiple stakeholder groups, including researchers, people with MS, PD and stroke, clinicians, and service-planners/policymakers, to achieve consensus on the most important outcomes. The use of an online Delphi process meant that the views and expertise of individuals from a range of countries could be captured. Furthermore, the recommendation of a measurement instrument to accompany each outcome in the COS means that it is readily implementable. Despite being presented in the survey rounds, no condition-specific outcome met the criteria for inclusion in the final COS. Therefore, the COS developed as part of this study is suitable for use in the evaluation of both single-diagnosis and mixed-diagnosis falls prevention interventions for people with MS, PD and stroke, thereby facilitating comparison of intervention effect within and across neurological conditions. Finally, a multi-modal approach to the dissemination of this COS to promote its uptake has been articulated in the protocol, including publication of the COS in an open-access peer-reviewed journal, presentation of the COS at national and international conferences, and dissemination of the COS through relevant professional and patient organisations [22].

Although we sought international participation in this study, our surveys were only available in English due to time and resource constraints. Our aim was to retain a minimum sample of 40 participants, with approximately 10 participants per stakeholder group [59]. While a total of 42 participants did complete all three survey rounds, nine of those individuals had a diagnosis of MS, PD or stroke, and eight were involved in service-planning/policymaking. The smaller sample of service-planners/policymakers is likely reflective of the smaller pool to recruit from but, importantly, no service-planners/policymakers were lost to follow-up throughout the study process, demonstrating the interest of that stakeholder group in the COS. The majority of patient participants lost to follow-up was due to illness. Additional efforts were made throughout this study process to ensure that the patient perspective was captured, including the establishment of a PPI panel, the completion of primary qualitative research, and the need for at least one patient participant in attendance at the consensus meeting to vote to include an outcome for it to meet the criteria. All participants who completed the second round of the survey were invited to participate in the consensus meeting; however, it was primarily researchers who expressed interest and subsequently attended. We considered restricting the number of researchers who attended the meeting but, ultimately, decided that the composition of the group who attended the meeting largely reflected the composition of the group of Delphi survey respondents. It must also be considered that the findings of the consensus meeting may have been different had other participants attended. However, the consensus meeting is a key element of the Delphi process as it allows for controlled discussion and debate, and the research team made efforts to ensure that the viewpoints of those who were not in attendance were considered by providing a breakdown of the Delphi survey results for each outcome. A pragmatic approach to the identification and selection of outcome measures was taken due to time limitations of this study, with targeted searches used to identify the best available evidence for the outcome measures proposed. The outcome measures recommended for use received high levels of agreement for inclusion from attendees at the consensus meeting, but the authors acknowledge that these recommendations may need to be updated based on future research.

## Conclusions

This is the first study to define a COS and accompanying measurement instruments for evaluating the effectiveness of mixed-diagnosis falls prevention interventions for people with MS, PD and stroke. Core outcomes identified through this consensus process include fall incidence, injurious fall incidence, quality of life, falls self-efficacy, fear of falling, activity curtailment due to fear of falling, and cost-effectiveness. When assessing and reporting additional outcomes, researchers should articulate the proposed mechanism of impact of the intervention. Uptake of this COS in all future trials will facilitate consistent reporting, improve evidence synthesis and reduce research waste to accelerate the development and evaluation of interventions, ultimately resulting in advancements in this research field.

## Supporting information

S1 Appendix Outcomes included in the Delphi process. (PDF)

S2 Appendix Attrition assessment between Delphi survey rounds. (PDF)

S3 Appendix Summary of survey responses from round one. (PDF)

S4 Appendix Summary of survery responses from round two. (PDF)

S5 Appendix Summary of survey responses from round three. (PDF)

S6 Appendix Summary of the categorisation of outcomes following completion of the Delphi survey rounds. (PDF)

S7 Appendix Summary of the final core outcome set for evaluating the effectiveness of mixed diagnosis falls prevention interventions for people with Multiple Sclerosis, Parkinson’s Disease and stroke. (PNG)
