# Supplementary material for: A core outcome set for evaluating the effectiveness of mixed-diagnosis falls prevention interventions for people with Multiple Sclerosis, Parkinson’s Disease and stroke
Source: PLoS One. 2023 Nov 13;18(11):e0294193. doi: 10.1371/journal.pone.0294193 (PMC10642845; doi:10.1371/journal.pone.0294193)
Supplement: S1 Appendix — (PDF) [file pone.0294193.s001.pdf]

Appendix 1: Outcomes included in the Delphi process.

Outcome	Source
Self-reported ability to perform activities of daily living	Umbrella review
Objectively assessed ability to perform activities of daily living	Umbrella review
Ability to engage in social activities	Umbrella review
Self-efficacy	Umbrella review
Static balance	Umbrella review
Dynamic balance	Umbrella review
Cognition	Umbrella review
Cost-effectiveness	Umbrella review
Disease impact	Umbrella review
Disease severity	Umbrella review
Dizziness	Umbrella review
Total number of falls	Umbrella review
Falls rate	Umbrella review
Number of fallers	Umbrella review
Number of recurrent fallers	Umbrella review
Number of injurious falls	Umbrella review
Number of falls resulting in healthcare utilisation	Umbrella review
Number of fall-related fractures	Umbrella review
Time to first post-intervention fall	Umbrella review
Number of near falls	Umbrella review
Falls risk	Umbrella review
Fatigue severity	Umbrella review
Fatigue impact	Umbrella review
Fear of falling	Umbrella review
Falls self-efficacy	Umbrella review
Balance confidence	Umbrella review
Level of physical activity	Umbrella review
Lower limb strength	Umbrella review
Anxiety	Umbrella review
Depression	Umbrella review

Objectively assessed mobility	Umbrella review
Self-reported mobility	Umbrella review
Walking distance	Umbrella review
Walking speed	Umbrella review
Stride length	Umbrella review
Cadence	Umbrella review
Walking self-efficacy	Umbrella review
Quality of life	Umbrella review
Sleep quality	Umbrella review
Ability to independently perform activities of daily living	Qualitative study
Bone density	Qualitative study
Self-perceived impact on carer/family	Qualitative study
Endurance	Qualitative study
Number of falls resulting in a long lie	Qualitative study
Activity curtailment due to fear of falling	Qualitative study
Fitness	Qualitative study
Flexibility	Qualitative study
Knowledge of how to fall	Qualitative study
Knowledge of how to get up from the floor after a fall	Qualitative study
Pain	Qualitative study
Falls self-management skills	Qualitative study
Understanding of personal falls risk factors	Qualitative study
Impact on carer	Stakeholder group
Bradykinesia	Stakeholder group
Freezing of gait	Stakeholder group
Dual-tasking ability	Stakeholder group
Perceived control of falls	Stakeholder group
Peer-support	Stakeholder group
Falls rate adjusted for activity exposure	Survey respondent
Time spent out of bed during daytime	Survey respondent
Joining a support/community group	Survey respondent
Changes to home/work environment	Survey respondent
