# Supplementary material for: A core outcome set for evaluating the effectiveness of mixed-diagnosis falls prevention interventions for people with Multiple Sclerosis, Parkinson’s Disease and stroke
Source: PLoS One. 2023 Nov 13;18(11):e0294193. doi: 10.1371/journal.pone.0294193 (PMC10642845; doi:10.1371/journal.pone.0294193)
Supplement: S2 Appendix — (PDF) [file pone.0294193.s002.pdf]

Appendix 2: Attrition assessment between Delphi survey rounds.

**Outcome**	**First round results for responders to second round (n=43)** Median (IQR)	**First round results for non-responders to second round (n=5)** Median (IQR)	**Second round results for responders to third round (n=42)** Median (IQR)	**Second round results for non-responder to third round (n=1)**
**Self-reported ability to perform activities of daily living**	6 (5-8)	7 (4-9)	6 (5-7)	6
**Objectively assessed ability to perform activities of daily living**	7 (6-9)	8 (5.75-8.75)	7 (6-9)	7
**Ability to engage in social activities**	7 (5-8)	5 (5-8)	6.5 (5-8)	6
**Self-efficacy**	7 (6-8)	7 (6-9)	7.5 (5.75-8)	6
**Static balance**	7 (5-8)	7 (6.5-9)	7 (5-8)	6
**Dynamic balance**	8 (7-9)	9 (8-9)	8 (7-9)	8
**Cognition**	8 (5-9)	8 (6.5-9)	7 (5-9)	7
**Cost-effectiveness**	7 (5-8)	5 (2.5-6)	7 (5-8)	7
**Disease impact**	7 (5-8)	9 (7-9)	6 (4.75-8)	6
**Disease severity**	6 (3-8)	8 (6.5-9)	5 (2.75-8)	5
**Dizziness**	6 (5-8)	7 (5.5-8.5)	5 (4-8)	7
**Total number of falls**	8 (7-9)	8 (7.25-8.75)	8.5 (7-9)	7
**Falls rate**	9 (7-9)	6 (5-8.5)	8.5 (7-9)	7
**Number of fallers**	7 (5-9)	7 (5.5-7.5)	7.5 (5-8.25)	7
**Number of recurrent fallers**	8 (6-9)	7 (5.-7.5)	8 (5-9)	7
**Number of injurious falls**	8 (7-9)	5 (4.5-5.5)	8 (7-9)	8
**Number of falls resulting in healthcare utilisation**	7 (6-9)	6.5 (5-7.5)	8 (6-9)	7

**Number of fall-related fractures**	8 (7-9)	5 (4-7)	8 (6-9)	7
**Time to first post-intervention fall**	7 (6-8)	6 (4.5-7)	7 (5-8)	7
**Number of near falls**	6 (4-7.5)	6 (4.5-7.5)	6 (5-7)	4
**Falls risk**	7 (5-9)	5 (5-7.5)	7 (5-8)	8
**Fatigue severity**	6 (5-7)	6 (6-9)	5 (4-7)	4
**Fatigue impact**	7 (5-8)	6 (5-8)	5.5 (4.75-7)	4
**Fear of falling**	8 (7-9)	5 (4.5-7.5)	8 (7-9)	7
**Falls self-efficacy**	7 (5-8)	7 (5-8.5)	7 (6-8)	8
**Balance confidence**	7 (6-8)	7 (4.5-8.5)	7 (6-8)	6
**Level of physical activity**	7 (5-8)	7 (5-8)	7 (5-8)	6
**Lower limb strength**	7 (6-8)	8 (6.5-9)	7 (5-8)	6
**Anxiety**	6 (5-7)	5 (4-7.5)	6 (4.75-7)	5
**Depression**	6 (4-7)	5 (4-7)	5 (3.75-7)	5
**Objectively assessed mobility**	7 (6-9)	7 (5-8.5)	7.5 (6-9)	6
**Self-reported mobility**	6 (5-7)	5 (5-7.5)	6 (5-7)	5
**Walking distance**	6 (5-7)	7 (5.5-9)	6 (4.75-7)	6
**Walking speed**	6 (5-8)	5 (5-6)	6 (3.75-7)	6
**Stride length**	5 (4-7)	5 (4.5-5.5)	5 (3.75-6.25)	2
**Cadence**	5 (4-7)	5 (4.5-5.5)	5 (3-6)	2
**Walking self-efficacy**	6 (5-8)	5 (5-7)	5 (5-6)	5
**Quality of life**	8 (6-9)	9 (7-9)	8 (6-9)	7
**Sleep quality**	5 (3-7)	7 (5.5-9)	5 (3-6.25)	6
**Ability to independently perform activities of daily living**	7 (5-8)	7 (5-8.5)	7 (5-8)	5
**Bone density**	5 (2-7)	5 (5-8)	5 (2-5)	2
**Self-perceived impact on carer/family**	6 (5-7)	7 (5-8.5)	5 (4-6.25)	4
**Endurance**	6 (5-7)	6 (5-8)	5 (5-7)	6

**Number of falls resulting in a long lie**	8 (6-9)	7 (6.5-8.5)	7.5 (6-9)	6
**Activity curtailment due to fear of falling**	8 (7-9)	6 (5-8)	8 (8-9)	7
**Fitness**	5 (5-6)	7 (5-8.5)	5 (4-6)	2
**Flexibility**	5 (3-6)	6 (5-6.5)	5 (3-6)	2
**Knowledge of how to fall**	6 (5-8)	7 (4.5-8.5)	5 (4-7.25)	8
**Knowledge of how to get up from the floor after a fall**	7 (5-8)	8 (6-9)	7 (5-8)	7
**Pain**	6 (5-8)	5 (5-9)	5 (3-7)	2
**Falls self-management skills**	8 (7-9)	7 (5-8.5)	8 (7-9)	7
**Understanding of personal falls risk factors**	7 (6-9)	7 (5.5-8)	7 (6-8.25)	7
**Impact on carer**	5 (5-7)	5 (5-6)	6 (5-7)	6
**Bradykinesia**	5 (4-7)	7 (3.5-9)	5 (3-6.25)	5
**Freezing of gait**	7 (5-8)	8.5 (5.75-9)	6 (4.75-7.25)	6
**Dual-tasking ability**	6 (5-8)	6 (5-9)	6 (5-8)	6
**Perceived control of falls**	7 (5-8)	6 (5-8)	6.5 (5-8)	7
**Peer-support**	5 (3-8)	6 (2.5-7)	5 (3-7.25)	5
**Falls rate adjusted for activity exposure**	N/A	N/A	7 (5.75-8)	6
**Time spent out of bed during daytime**	N/A	N/A	5 (4-7)	2
**Joining a support/ community group**	N/A	N/A	6 (5-7)	2
**Changes to home/work environment**	N/A	N/A	6 (5-7)	6
