# Supplementary material for: A core outcome set for evaluating the effectiveness of mixed-diagnosis falls prevention interventions for people with Multiple Sclerosis, Parkinson’s Disease and stroke
Source: PLoS One. 2023 Nov 13;18(11):e0294193. doi: 10.1371/journal.pone.0294193 (PMC10642845; doi:10.1371/journal.pone.0294193)
Supplement: S3 Appendix — (PDF) [file pone.0294193.s003.pdf]

Appendix 3: Summary of survey responses from round one.

Activity curtailment due to fear of falling

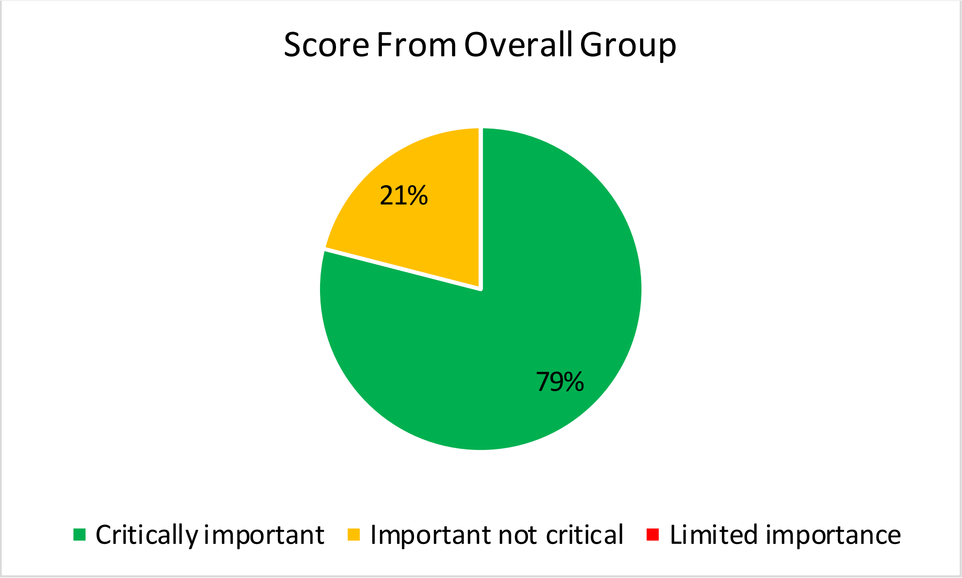

Scores From Each Stakeholder Group

Patients
Critically important: 75%
Important not critical: 25%
Limited importance: 0%

Researchers
Critically important: 82%
Important not critical: 18%
Limited importance: 0%

Clinicians
Critically important: 75%
Important not critical: 25%
Limited importance: 0%

Service-planners/Policymakers
Critically important: 86%
Important not critical: 14%
Limited importance: 0%

Reasons For Scores

Please note that the number in brackets shows the percentage of participants that gave that reason.

<u>Reasons for including:</u>
Participation in activities is important for overall health (4%).
Enables positive risk taking (4%).

<u>Reasons for excluding:</u>
Not relevant to all interventions only those where the goal of the intervention is to reduce fear of falling and associated avoidance of activities (2%).

Anxiety

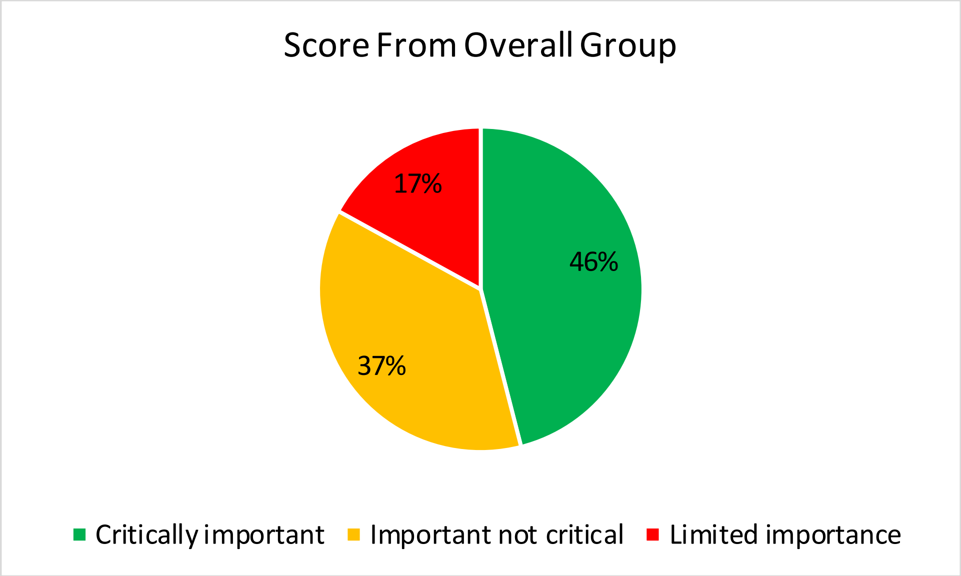

Scores From Each Stakeholder Group

Patients
Critically important: 67%
Important not critical: 33%
Limited importance: 0%

Researchers
Critically important: 18%
Important not critical: 53%
Limited importance: 29%

Clinicians
Critically important: 42%
Important not critical: 42%
Limited importance: 16%

Service-planners/Policymakers
Critically important: 86%
Important not critical: 0%
Limited importance: 14%

Reasons For Scores

Please note that the number in brackets shows the percentage of participants that gave that reason.

Reasons for including:
Contributes to fear of falling (4%).
May prevent patients from engaging in activities (2%).
Related to falls (2%).
Limits balance reactions and progression (2%).

Reasons for excluding:
Not relevant to all interventions only those where the goal is to prevent falls by improving anxiety (2%).

Balance confidence

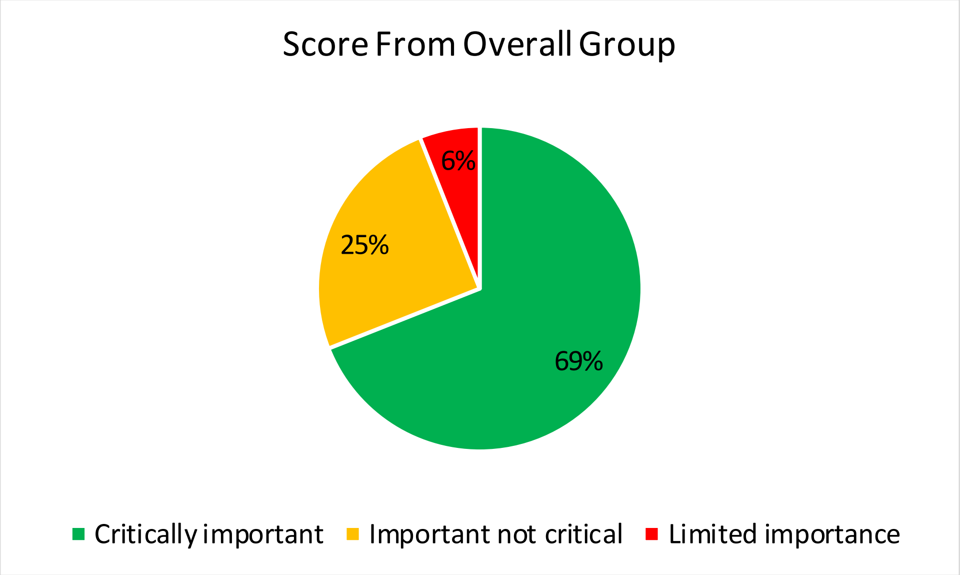

Scores From Each Stakeholder Group

Patients
Critically important: 84%
Important not critical: 8%
Limited importance: 8%

Researchers
Critically important: 53%
Important not critical: 41%
Limited importance: 6%

Clinicians
Critically important: 67%
Important not critical: 25%
Limited importance: 8%

Service-planners/Policymakers
Critically important: 86%
Important not critical: 14%
Limited importance: 0%

Reasons For Scores

Please note that the number in brackets shows the percentage of participants that gave that reason.

Reasons for including:
Could reduce fear of falling (2%).

Reasons for excluding:
Confidence may decrease as awareness improves, and this awareness may be the mechanism by which falls are reduced – confidence does not have a linear association with falls (6%).
Overlaps with falls self-efficacy (2%).
Over-confidence can result in falls (2%).

Bone density

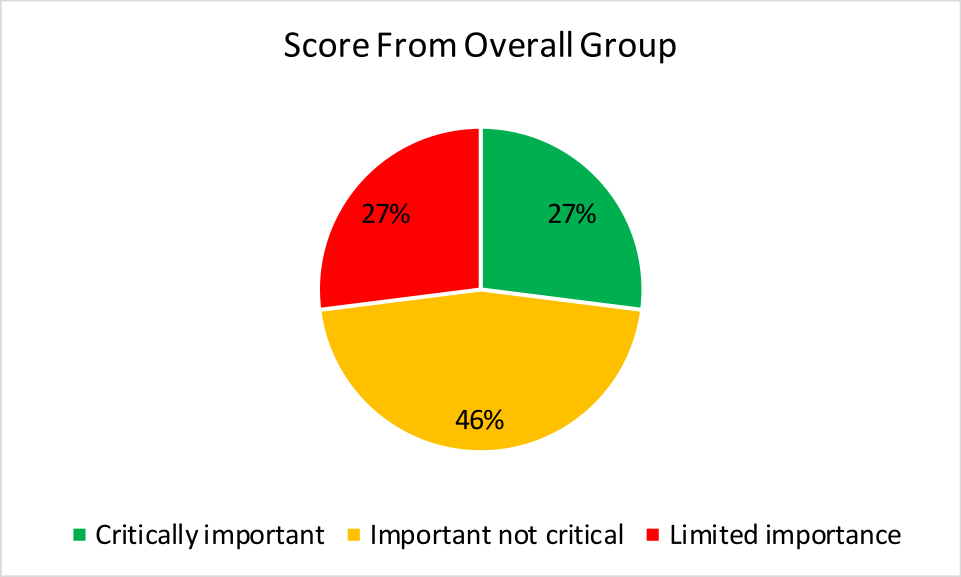

Scores From Each Stakeholder Group

Patients
Critically important: 50%
Important not critical: 25%
Limited importance: 25%

Researchers
Critically important: 6%
Important not critical: 41%
Limited importance: 53%

Clinicians
Critically important: 17%
Important not critical: 75%
Limited importance: 8%

Service-planners/Policymakers
Critically important: 57%
Important not critical: 43%
Limited importance: 0%

Reasons For Scores

Please note that the number in brackets shows the percentage of participants that gave that reason.

Reasons for including:
Osteoporosis presents greater risk for a fall-related fracture (8%).
Bone density provides the structural frame from which stability is enabled and movement is possible (2%).

Reasons for excluding:
Do not expect bone density to change with a falls prevention intervention (6%).
Logistics/funding available to allow for bone density assessment (6%) and longer timeline required to show improvement in bone density at reassessment (2%) would need to be considered.
Bone density is relevant to the consequences of falls, it does not affect their frequency (4%).
Unaware of high-quality evidence to support bone density as a falls risk factor for all three conditions (2%).
Not relevant to all interventions only those targeting a reduction in fractures post-fall (2%).
Not related to the primary goal of the intervention (2%).

Bradykinesia

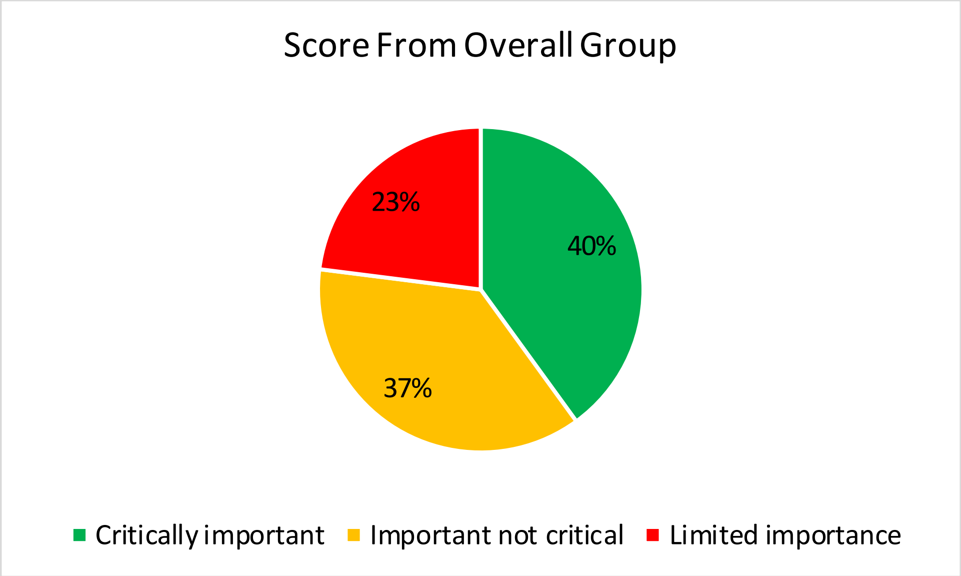

Scores From Each Stakeholder Group

Patients
Critically important: 50%
Important not critical: 33%
Limited importance: 17%

Researchers
Critically important: 12%
Important not critical: 41%
Limited importance: 47%

Clinicians
Critically important: 50%
Important not critical: 50%
Limited importance: 0%

Service-planners/Policymakers
Critically important: 72%
Important not critical: 14%
Limited importance: 14%

Reasons For Scores

Please note that the number in brackets shows the percentage of participants that gave that reason.

Reasons for including:
Impacts reaction time (2%).

Reasons for excluding:
Do not expect bradykinesia to change with a falls prevention intervention (4%).
Not shown to be a falls risk factor (4%).
Not relevant to all interventions only those that aim to prevent falls by improving bradykinesia (4%).
Slowing down and concentrating is a strategy so slowing down may be a positive result for some patients (2%).
Largely influenced by drug management (2%).
Size, intensity and complexity of movement more important than speed (2%).
Better to show the impact on function rather than as a symptom (2%).
Reversibility becomes more difficult as disease progresses (2%).

Cadence

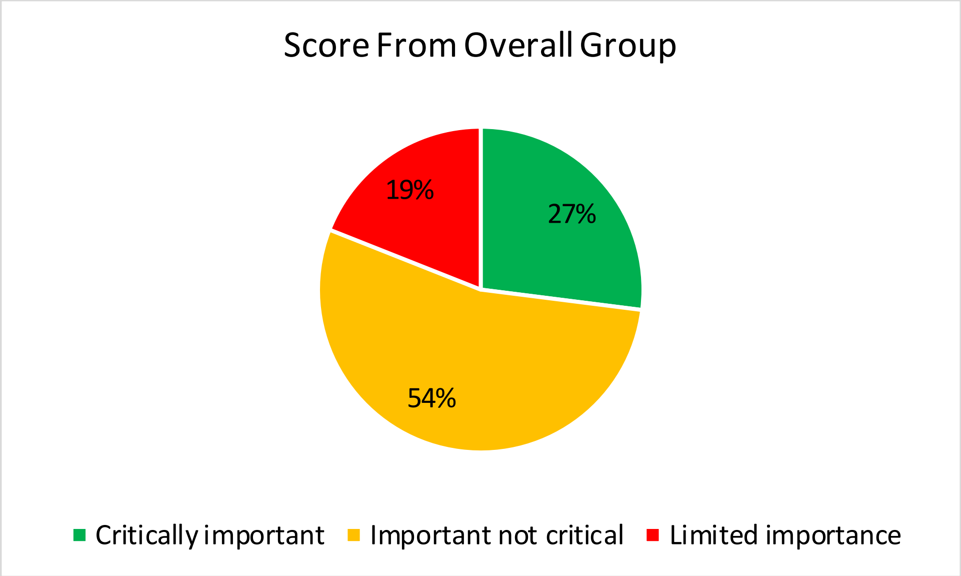

Scores From Each Stakeholder Group

Patients
Critically important: 25%
Important not critical: 75%
Limited importance: 0%

Researchers
Critically important: 12%
Important not critical: 41%
Limited importance: 47%

Clinicians
Critically important: 33%
Important not critical: 59%
Limited importance: 8%

Service-planners/Policymakers
Critically important: 57%
Important not critical: 43%
Limited importance: 0%

Reasons For Scores

Please note that the number in brackets shows the percentage of participants that gave that reason.

Reasons for including:
For assessment of balance (2%).

Reasons for excluding:
Excessive cadence, such as in Parkinson's, can lead to falls (2%).
Walking slower may be a positive outcome for some patients (2%).
Covered by walking speed (2%).
Not related to the primary goal of the intervention (2%).

Impact on carer

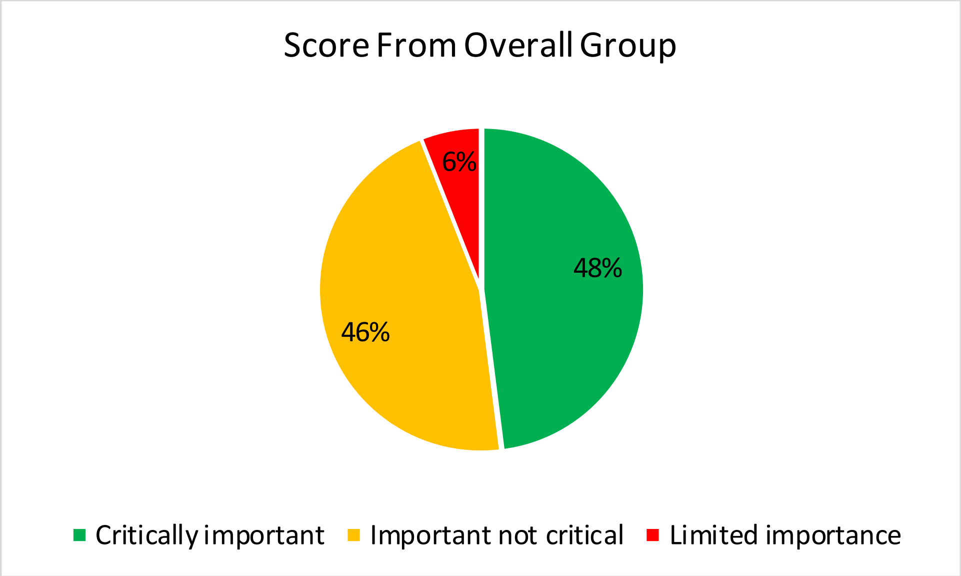

Scores From Each Stakeholder Group

Patients
Critically important: 42%
Important not critical: 50%
Limited importance: 8%

Researchers
Critically important: 35%
Important not critical: 53%
Limited importance: 12%

Clinicians
Critically important: 58%
Important not critical: 42%
Limited importance: 0%

Service-planners/Policymakers
Critically important: 71%
Important not critical: 29%
Limited importance: 0%

Reasons For Scores

Please note that the number in brackets shows the percentage of participants that gave that reason.

Reasons for including:
To ensure sufficient mutual supports are available (2%).
Carers provide unique perspectives and information giving a clearer picture of the impact of an intervention (2%).

Reasons for excluding:
Not applicable to all patients as many do not have a carer (8%).
Impact on carer will give insights but not those linked directly to falls risk outcomes (2%).
Not all carers are impacted the same way (depending on factors such as relationship to patient/if they are full or part-time carers) and so should not be compared (2%).

Cognition

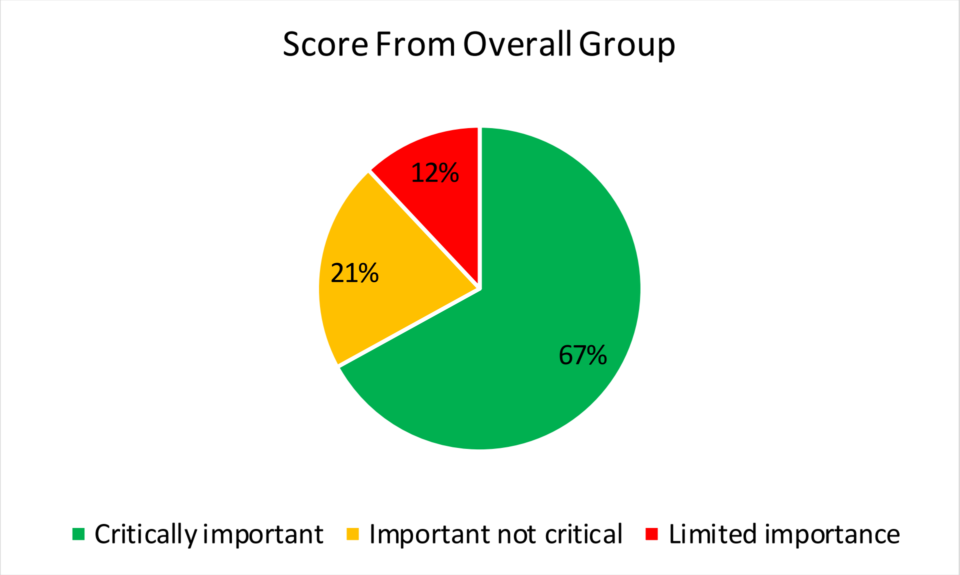

Scores From Each Stakeholder Group

Patients
Critically important: 83%
Important not critical: 17%
Limited importance: 0%

Researchers
Critically important: 35%
Important not critical: 41%
Limited importance: 24%

Clinicians
Critically important: 75%
Important not critical: 8%
Limited importance: 17%

Service-planners/Policymakers
Critically important: 100%
Important not critical: 0%
Limited importance: 0%

Reasons For Scores

Please note that the number in brackets shows the percentage of participants that gave that reason.

Reasons for including:
Increases risk of falling (4%).
Cognitive impairment affects awareness and processing, and, therefore, safety (4%).

Reasons for excluding:
Do not expect cognition to change with a falls prevention intervention (8%).
Difficult to show change in cognition (2%).
Not relevant to all interventions only those specifically designed to target cognition (2%).
Should not be measured as an outcome but as indicator of the most appropriate type of treatment (2%).

Cost-effectiveness

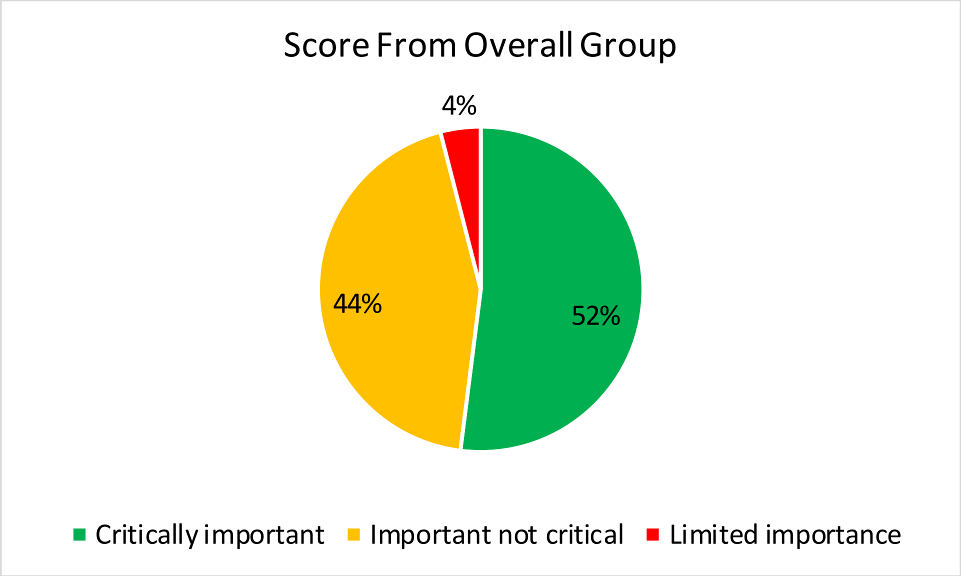

Scores From Each Stakeholder Group

Patients
Critically important: 50%
Important not critical: 33%
Limited importance: 17%

Researchers
Critically important: 71%
Important not critical: 29%
Limited importance: 0%

Clinicians
Critically important: 17%
Important not critical: 83%
Limited importance: 0%

Service-planners/Policymakers
Critically important: 71%
Important not critical: 29%
Limited importance: 0%

Reasons For Scores

Please note that the number in brackets shows the percentage of participants that gave that reason.

Reasons for including:
Important for accessibility and inclusivity of intervention (6%).
Important for adoption within funded healthcare settings (4%).
Resources are finite (2%).

Reasons for excluding:
Any cost of intervention will be worth it given the possible consequences (medical and personal) of a fall (2%).

Depression

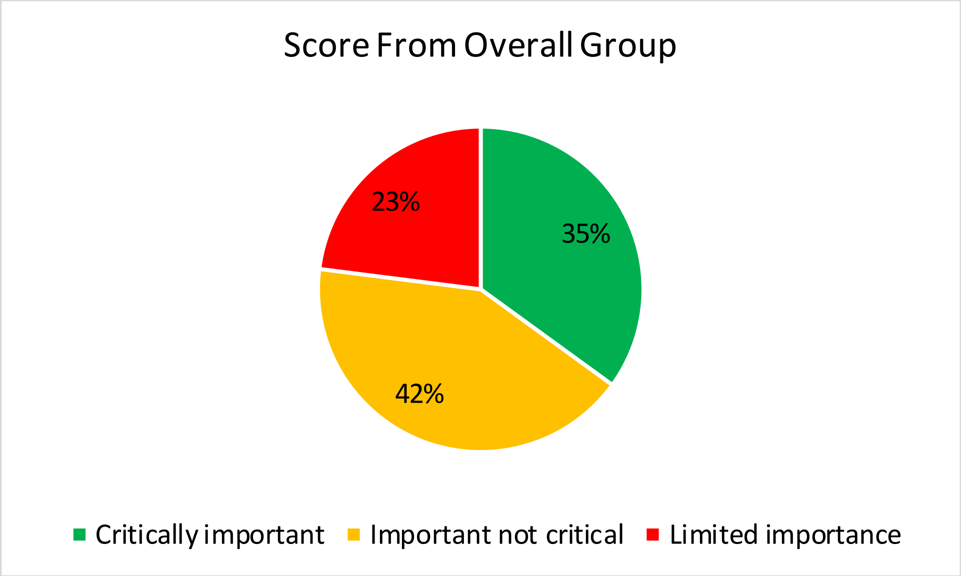

Scores From Each Stakeholder Group

Patients
Critically important: 50%
Important not critical: 33%
Limited importance: 17%

Researchers
Critically important: 24%
Important not critical: 41%
Limited importance: 35%

Clinicians
Critically important: 16%
Important not critical: 67%
Limited importance: 17%

Service-planners/Policymakers
Critically important: 72%
Important not critical: 14%
Limited importance: 14%

Reasons For Scores

Please note that the number in brackets shows the percentage of participants that gave that reason.

Reasons for including:
May prevent patients from engaging in activities (2%).
Could be linked to motivation (2%).
Limits carryover into daily life/function (2%).

Reasons for excluding:
Only relevant if related to the intervention goals/mechanisms of effectiveness (2%).

Disease impact

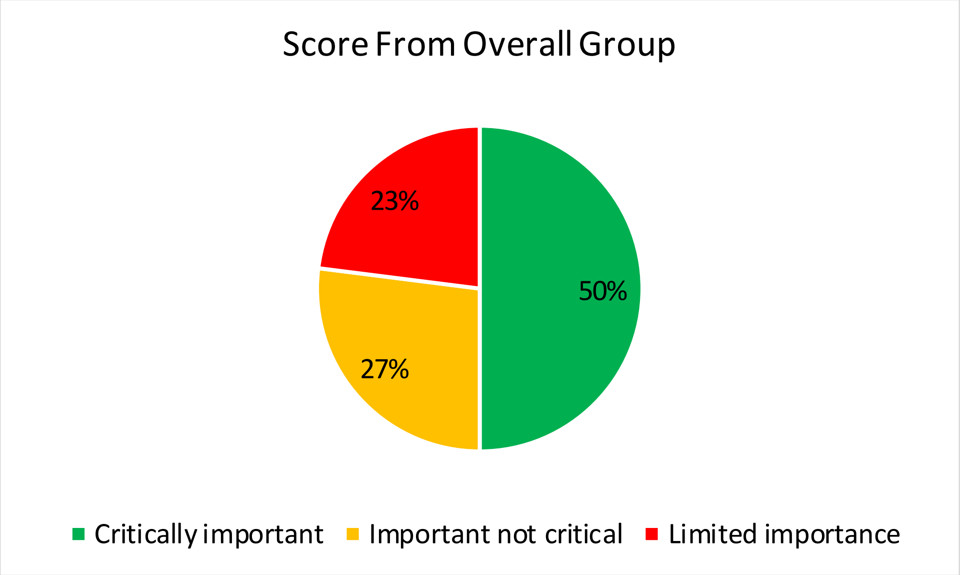

Scores From Each Stakeholder Group

Patients
Critically important: 75%
Important not critical: 25%
Limited importance: 0%

Researchers
Critically important: 23%
Important not critical: 18%
Limited importance: 59%

Clinicians
Critically important: 42%
Important not critical: 50%
Limited importance: 8%

Service-planners/Policymakers
Critically important: 86%
Important not critical: 14%
Limited importance: 0%

Reasons For Scores

Please note that the number in brackets shows the percentage of participants that gave that reason.

Reasons for including:
Has impact on quality of life (4%).
Patient feedback has suggested that it is more relevant to the patient than actual falls (2%).

Reasons for excluding:
Not related to the primary goal of the intervention (2%).
Hard to separate from other comorbidities (2%).
Factors other than falls can influence disease impact (2%).

Disease severity

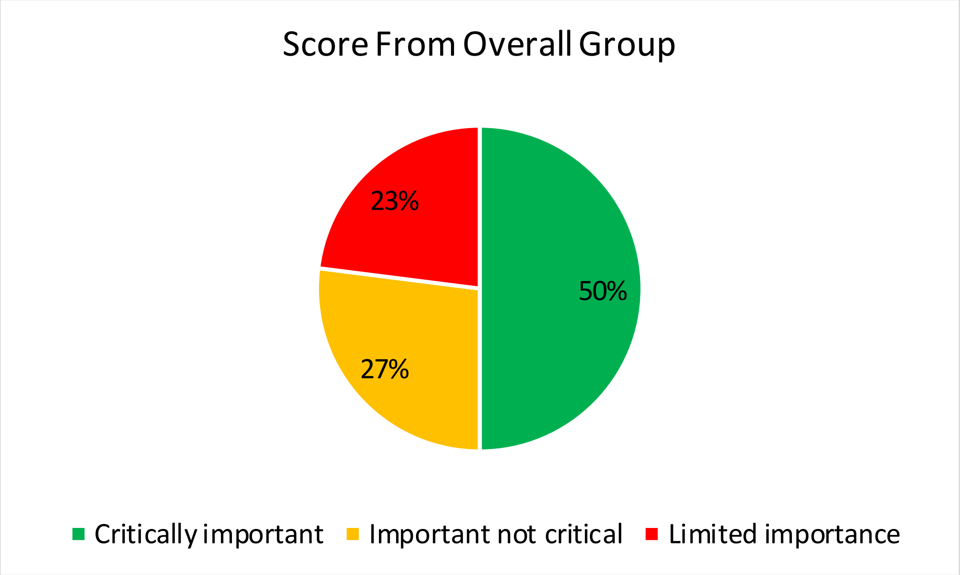

Scores From Each Stakeholder Group

Patients
Critically important: 75%
Important not critical: 25%
Limited importance: 0%

Researchers
Critically important: 23%
Important not critical: 18%
Limited importance: 59%

Clinicians
Critically important: 42%
Important not critical: 50%
Limited importance: 8%

Service-planners/Policymakers
Critically important: 86%
Important not critical: 14%
Limited importance: 0%

Reasons For Scores

Please note that the number in brackets shows the percentage of participants that gave that reason.

Reasons for including:
Increased falls risk with more severe disease (2%).

Reasons for excluding:
Do not expect a falls prevention intervention to change the disease itself (6%).
Not related to the primary goal of the intervention (2%).
Should not be included as an outcome but rather as a descriptor of the sample (2%).
Better to use functional outcomes (2%).

Dizziness

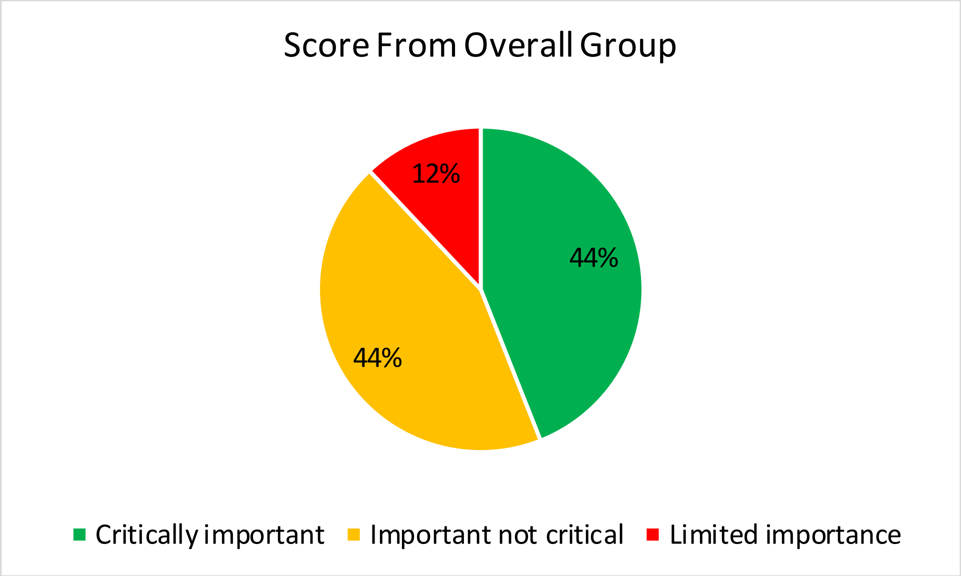

Scores From Each Stakeholder Group

Patients
Critically important: 75%
Important not critical: 17%
Limited importance: 8%

Researchers
Critically important: 18%
Important not critical: 59%
Limited importance: 23%

Clinicians
Critically important: 34%
Important not critical: 58%
Limited importance: 8%

Service-planners/Policymakers
Critically important: 71%
Important not critical: 29%
Limited importance: 0%

Reasons For Scores

Please note that the number in brackets shows the percentage of participants that gave that reason.

<u>Reasons for including:</u>
Could increase falls risk (6%).

<u>Reasons for excluding:</u>
Not relevant to all interventions only those that aim to prevent falls by improving dizziness (4%).
Do not expect a falls prevention intervention to improve dizziness (4%).
Not sure if research has shown dizziness to be a falls risk factor for all three conditions (2%).
Not all patients suffer from dizziness (2%).
Dizziness can be a result of other factors and may not be a result of neurological diagnosis (2%).

Dual-tasking ability

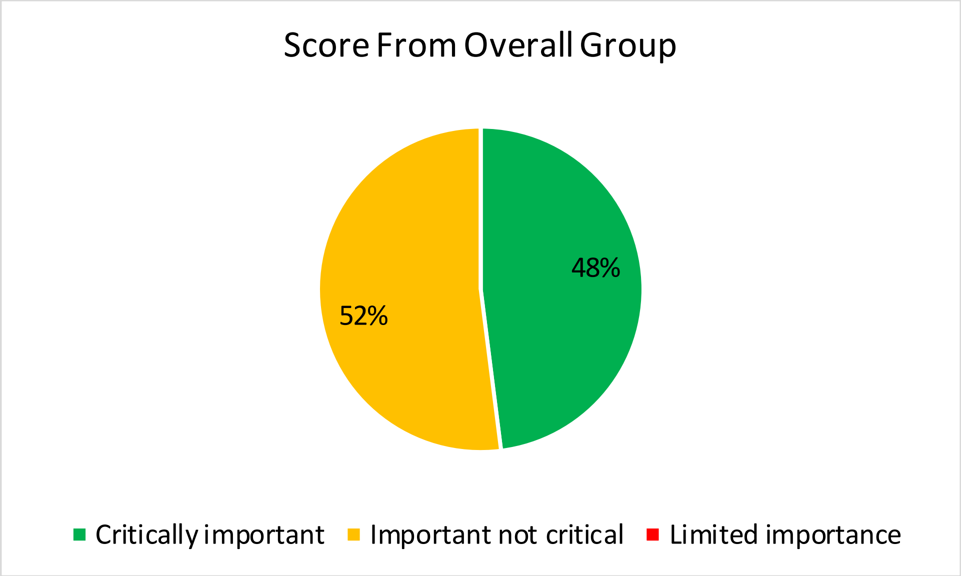

Scores From Each Stakeholder Group

Patients
Critically important: 67%
Important not critical: 33%
Limited importance: 0%

Researchers
Critically important: 24%
Important not critical: 76%
Limited importance: 0%

Clinicians
Critically important: 58%
Important not critical: 42%
Limited importance: 0%

Service-planners/Policymakers
Critically important: 57%
Important not critical: 43%
Limited importance: 0%

Reasons For Scores

Please note that the number in brackets shows the percentage of participants that gave that reason.

Reasons for including:
Dual-tasking occurs regularly in daily life (4%).
Inability to dual-task may result in loss of balance (2%).
Impacts independence, and safety with activities of daily living and walking (2%).

Reasons for excluding:
Not consistently identified as a falls risk factor (2%).
This outcome depends on the function, agency and expectations of each patient (2%).
Do not expect a falls prevention intervention to improve dual-tasking ability but rather to provide strategies to avoid it (2%).
Not relevant to all interventions only those that aim to prevent falls by improving cognitive function (2%).

Dynamic balance

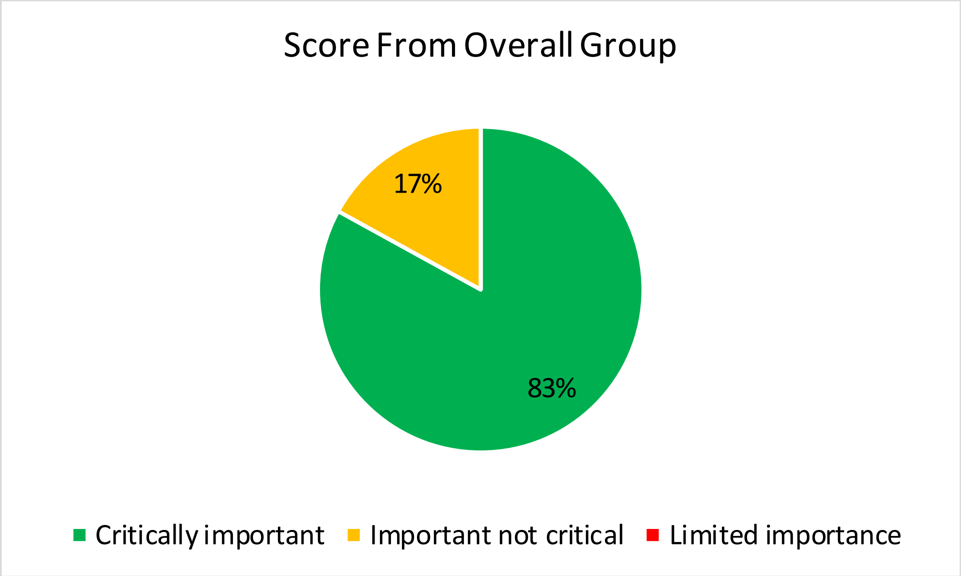

Scores From Each Stakeholder Group

Patients
Critically important: 100%
Important not critical: 0%
Limited importance: 0%

Researchers
Critically important: 71%
Important not critical: 29%
Limited importance: 0%

Clinicians
Critically important: 83%
Important not critical: 17%
Limited importance: 0%

Service-planners/Policymakers
Critically important: 86%
Important not critical: 14%
Limited importance: 0%

Reasons For Scores

Please note that the number in brackets shows the percentage of participants that gave that reason.

Reasons for including:
Risk factor for falls (6%).
Essential for safe completion of activities of daily living (6%).

Reasons for excluding:
Not relevant to all interventions only those where they goal is to prevent falls by improving balance control (2%).

Endurance

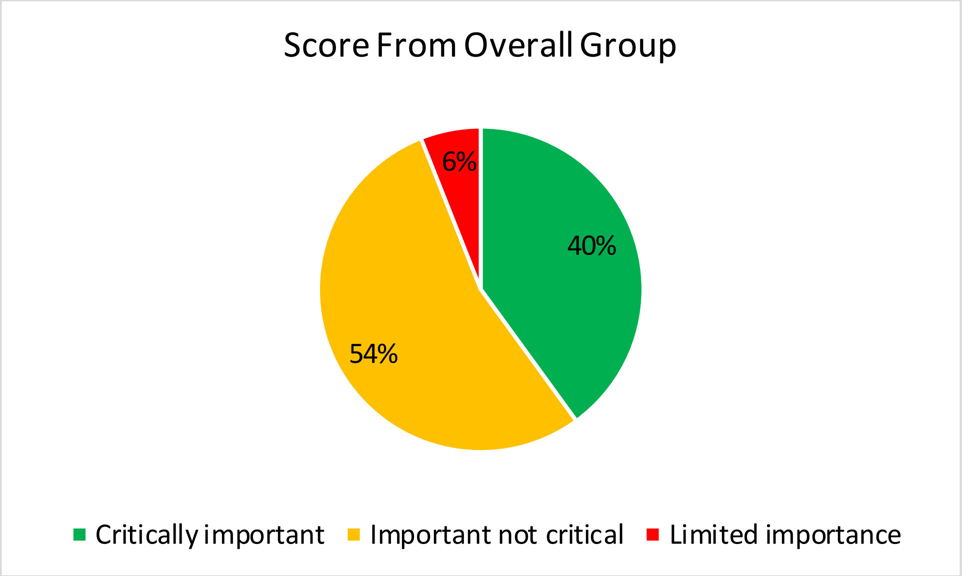

Scores From Each Stakeholder Group

Patients
Critically important: 50%
Important not critical: 50%
Limited importance: 0%

Researchers
Critically important: 23%
Important not critical: 59%
Limited importance: 18%

Clinicians
Critically important: 33%
Important not critical: 67%
Limited importance: 0%

Service-planners/Policymakers
Critically important: 71%
Important not critical: 29%
Limited importance: 0%

Reasons For Scores

Please note that the number in brackets shows the percentage of participants that gave that reason.

Reasons for including:
May increase risk of falling (4%).
Endurance is an important component of balance (2%).
Endurance allows patients to maintain social participation (2%).

Reasons for excluding:
This outcome depends on the function, agency and expectations of each patient (2%).
Not related to the primary goal of the intervention (2%).

Number of fall-related fractures

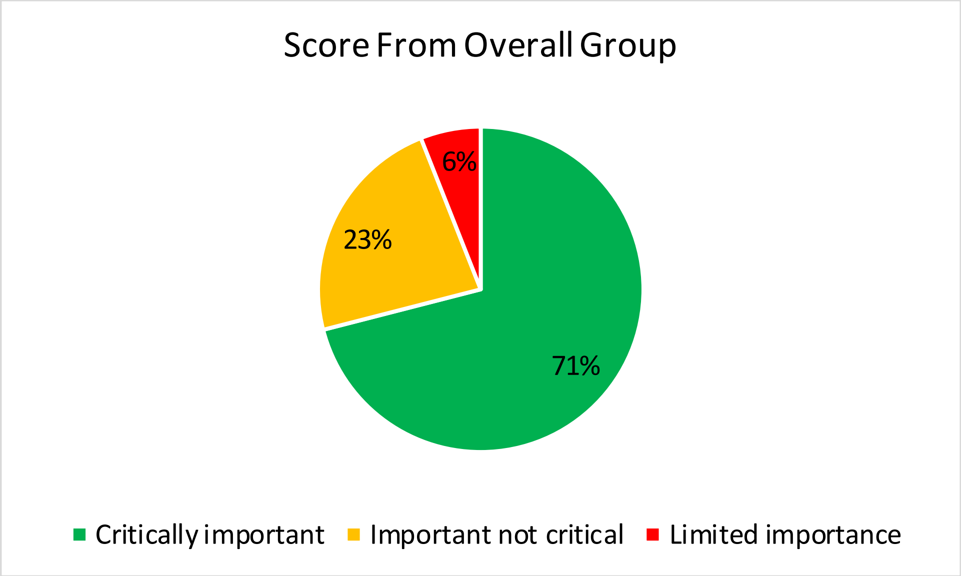

Scores From Each Stakeholder Group

Patients
Critically important: 42%
Important not critical: 33%
Limited importance: 25%

Researchers
Critically important: 82%
Important not critical: 18%
Limited importance: 0%

Clinicians
Critically important: 67%
Important not critical: 33%
Limited importance: 0%

Service-planners/Policymakers
Critically important: 100%
Important not critical: 0%
Limited importance: 0%

Reasons For Scores

Please note that the number in brackets shows the percentage of participants that gave that reason.

Reasons for including:
Fractures a serious injury with a long recovery time (2%).
Important from an independence and care provider standpoint (2%).
Data may prove useful for meta-analyses (2%).
Reducing the impact of treatment of falls on hospital spending is important (2%).

Reasons for excluding:
Fracture rates are low so most studies will not be powered to provide information on this outcome (4%).

Number of fallers

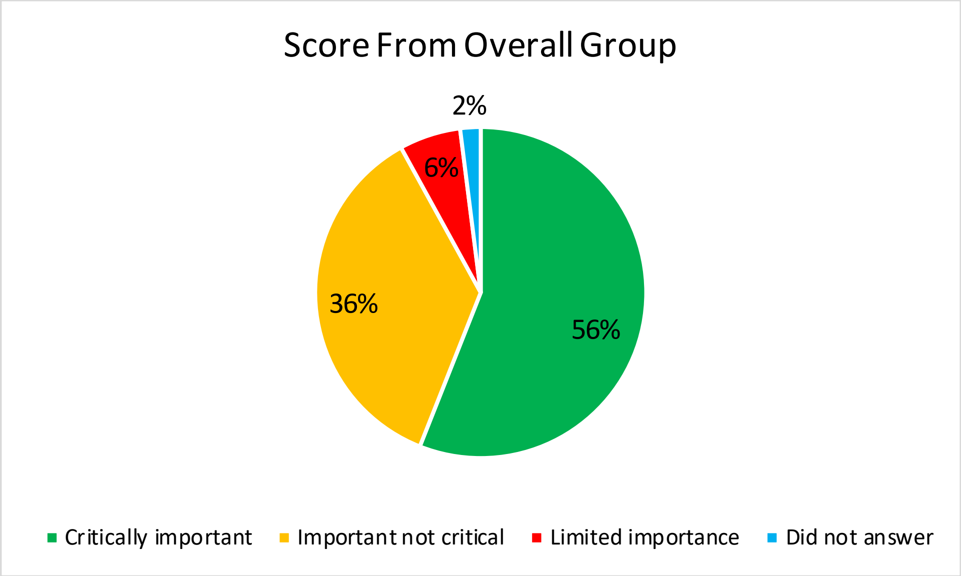

Scores From Each Stakeholder Group

Patients
Critically important: 50%
Important not critical: 34%
Limited importance: 8%
Did not answer: 8%

Researchers
Critically important: 70%
Important not critical: 18%
Limited importance: 12%

Clinicians
Critically important: 50%
Important not critical: 50%
Limited importance: 0%

Service-planners/Policymakers
Critically important: 43%
Important not critical: 57%
Limited importance: 0%

Reasons For Scores

Please note that the number in brackets shows the percentage of participants that gave that reason.

Reasons for excluding:
Depends on your baseline – if mostly non fallers then it could be a useful measure but if the cohort is mostly recurrent fallers then the outcome is not important as they are unlikely to stop falling entirely (2%).
Fall rates capture this better (2%).

Falls rate

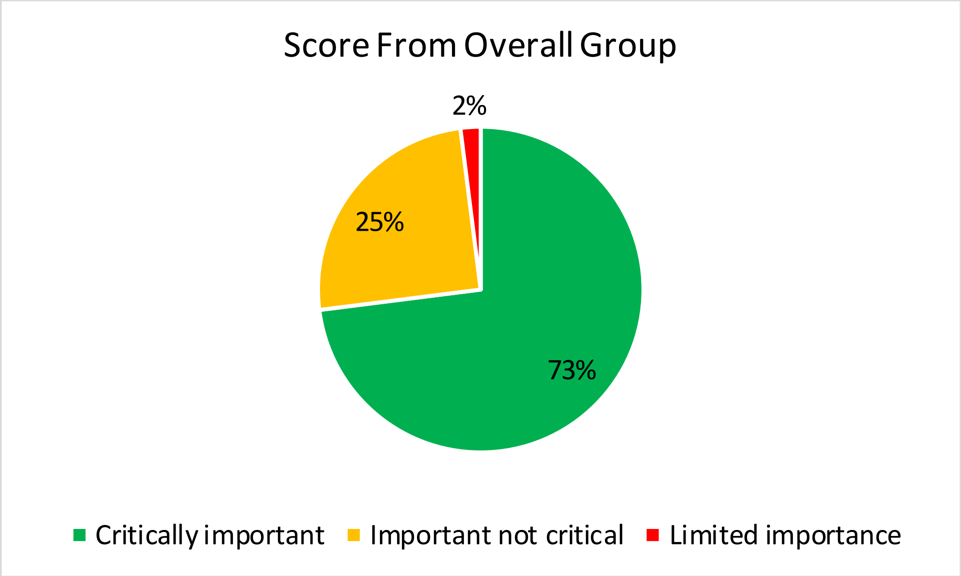

Scores From Each Stakeholder Group

Patients
Critically important: 59%
Important not critical: 33%
Limited importance: 8%

Researchers
Critically important: 94%
Important not critical: 6%
Limited importance: 0%

Clinicians
Critically important: 50%
Important not critical: 50%
Limited importance: 0%

Service-planners/Policymakers
Critically important: 86%
Important not critical: 14%
Limited importance: 0%

Number of falls resulting in healthcare utilisation

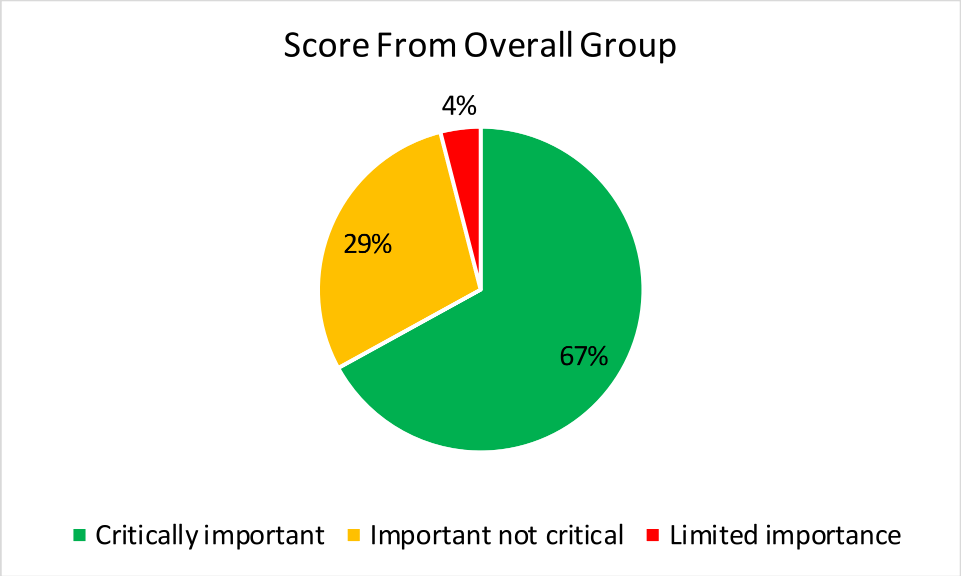

Scores From Each Stakeholder Group

Patients
Critically important: 50%
Important not critical: 33%
Limited importance: 17%

Researchers
Critically important: 76%
Important not critical: 24%
Limited importance: 0%

Clinicians
Critically important: 58%
Important not critical: 42%
Limited importance: 0%

Service-planners/Policymakers
Critically important: 86%
Important not critical: 14%
Limited importance: 0%

Reasons For Scores

Please note that the number in brackets shows the percentage of participants that gave that reason.

Reasons for including:
Relates to cost-effectiveness (8%).
Might indicate the severity of the injury (4%).
Data may prove useful for meta-analyses (2%).
Falls with harm lead to reduction in function and possible one-year mortality (2%).

Reasons for excluding:
Injuries that require medical attention are rare (4%) so most studies will not be powered to provide information on this outcome (2%).

Falls risk

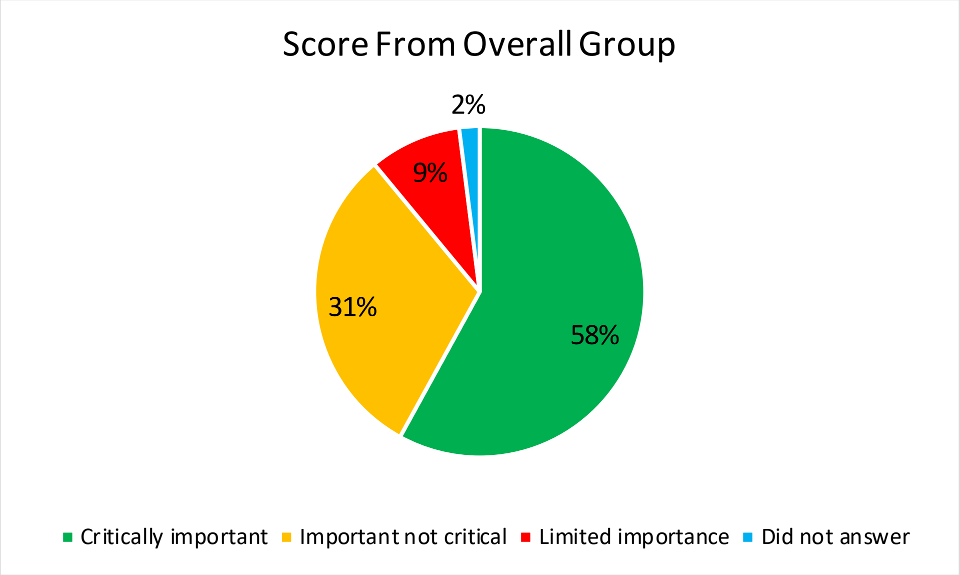

Scores From Each Stakeholder Group

Patients
Critically important: 58%
Important not critical: 42%
Limited importance: 0%

Researchers
Critically important: 41%
Important not critical: 35%
Limited importance: 18%
Did not answer: 6%

Clinicians
Critically important: 59%
Important not critical: 33%
Limited importance: 8%

Service-planners/Policymakers
Critically important: 100%
Important not critical: 0%
Limited importance: 0%

Reasons For Scores

Please note that the number in brackets shows the percentage of participants that gave that reason.

Of note, some participants who scored this outcome as critically important highlighted potential issues with this outcome including that it is difficult to measure (4%) and that its importance is dependent on how risk is quantified (4%).

Reasons for excluding:
Unclear how risk of falling is defined (6%).
Reducing risk but not the actual number of falls or injurious falls is not a successful intervention (4%).
Unclear how risk of falling is measured (2%).
Research data suggests that most falls risk measures are relatively unreliable (2%).
This is already captured with outcomes on falls in daily life (2%).

Falls self-efficacy

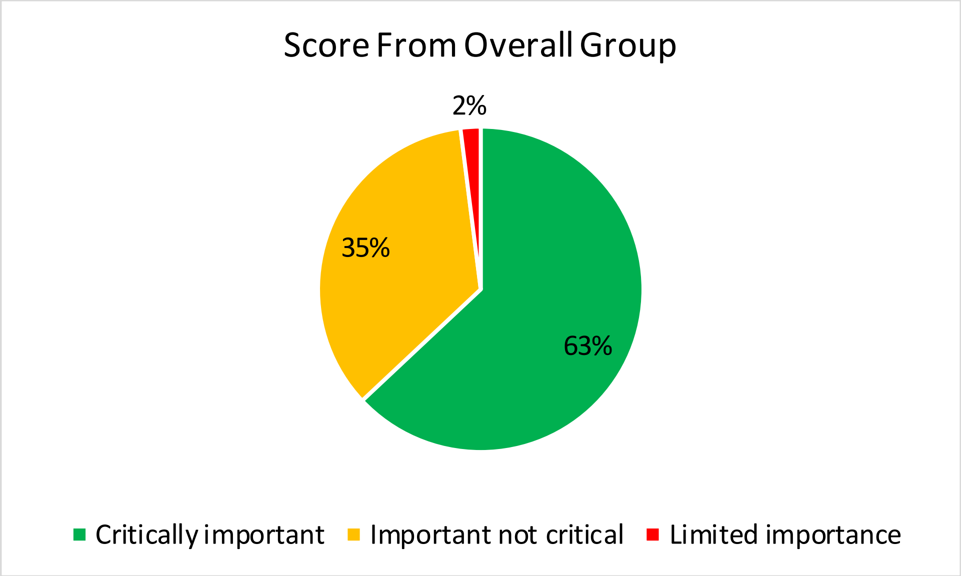

Scores From Each Stakeholder Group

Patients
Critically important: 59%
Important not critical: 33%
Limited importance: 8%

Researchers
Critically important: 76%
Important not critical: 24%
Limited importance: 0%

Clinicians
Critically important: 42%
Important not critical: 58%
Limited importance: 0%

Service-planners/Policymakers
Critically important: 71%
Important not critical: 29%
Limited importance: 0%

Reasons For Scores

Please note that the number in brackets shows the percentage of participants that gave that reason.

Reasons for including:
Could reduce fear of falling (2%).
Enables positive risk taking (2%).

Reasons for excluding:
Subjective measures can be inaccurate (2%).
Does not have a linear association with falls (2%).

Fatigue impact

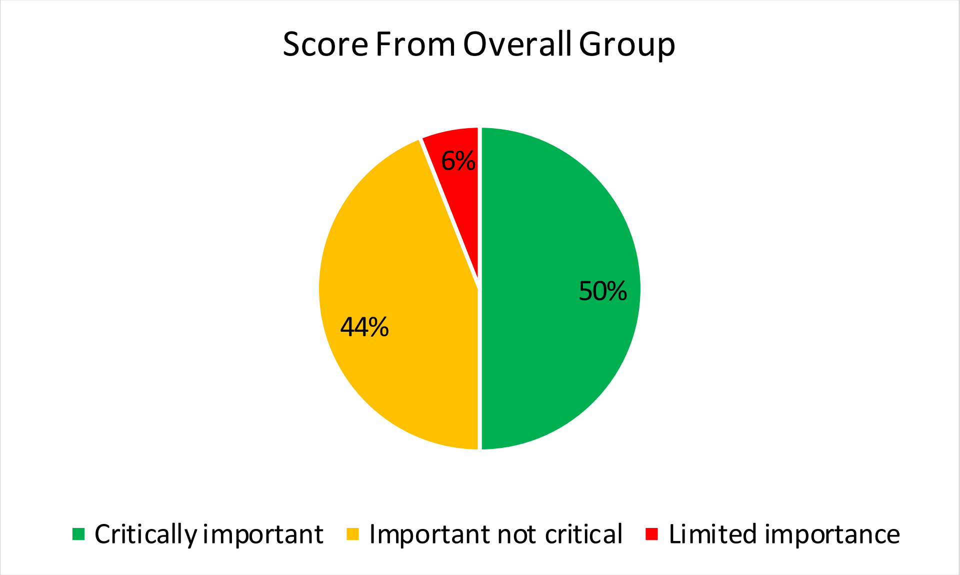

Scores From Each Stakeholder Group

Patients
Critically important: 83%
Important not critical: 17%
Limited importance: 0%

Researchers
Critically important: 29%
Important not critical: 53%
Limited importance: 18%

Clinicians
Critically important: 67%
Important not critical: 33%
Limited importance: 0%

Service-planners/Policymakers
Critically important: 71%
Important not critical: 29%
Limited importance: 0%

Reasons For Scores

Please note that the number in brackets shows the percentage of participants that gave that reason.

Reasons for including:
Impacts ability to do tasks and activities (4%).
May increase risk of falls (2%).

Reasons for excluding:
Do not expect a falls prevention intervention to change impact of fatigue (2%).
Not related to the primary goal of the intervention (2%).

Fatigue severity

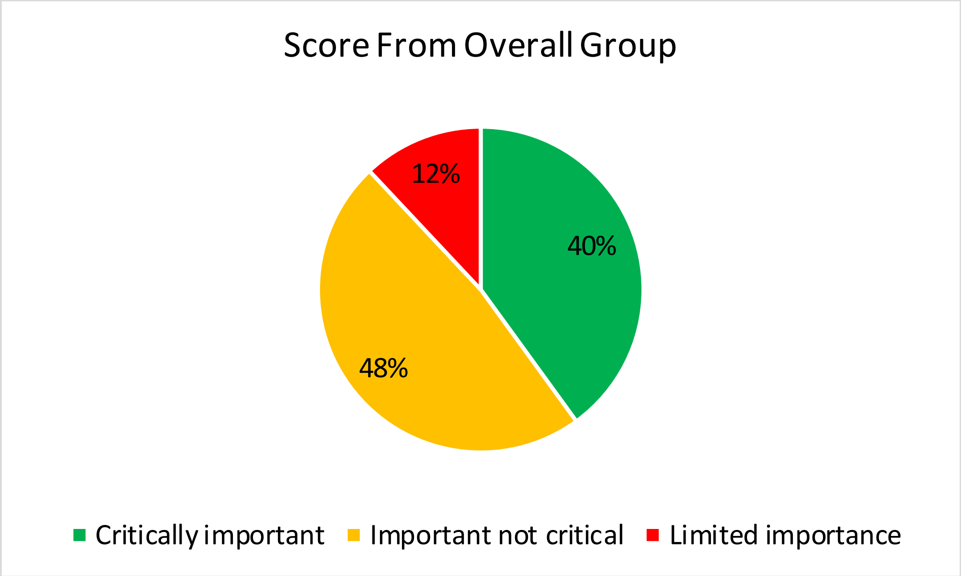

Scores From Each Stakeholder Group

Patients
Critically important: 75%
Important not critical: 25%
Limited importance: 0%

Researchers
Critically important: 12%
Important not critical: 59%
Limited importance: 29%

Clinicians
Critically important: 25%
Important not critical: 67%
Limited importance: 8%

Service-planners/Policymakers
Critically important: 71%
Important not critical: 29%
Limited importance: 0%

Reasons For Scores

Please note that the number in brackets shows the percentage of participants that gave that reason.

Reasons for including:
May increase risk of falls (4%).

Reasons for excluding:
Do not expect a falls prevention intervention to change fatigue severity (4%).
Not relevant to all interventions only those where the goal of the intervention is to prevent falls by reducing fatigue (2%).
Not related to the primary goal of the intervention (2%).

Fitness

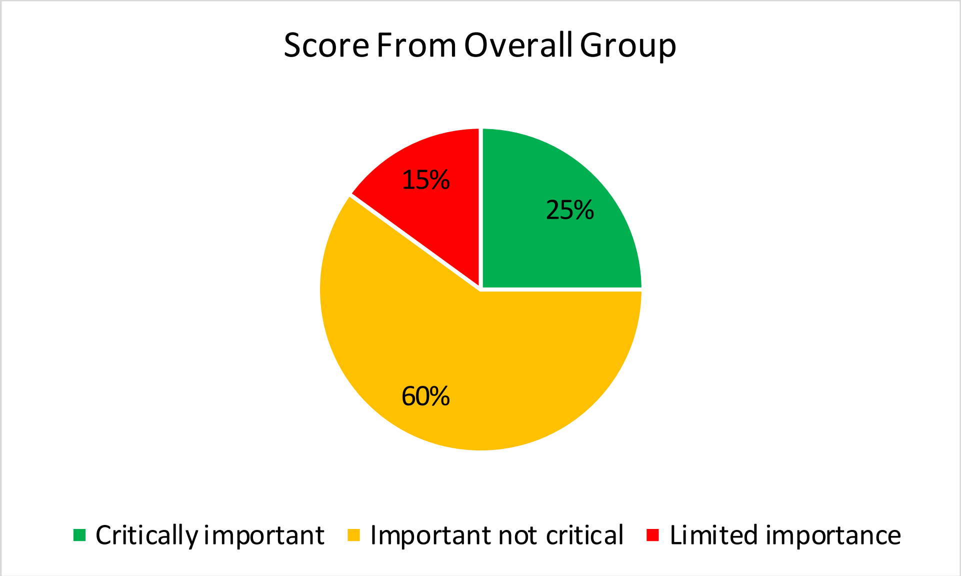

Scores From Each Stakeholder Group

Patients
Critically important: 67%
Important not critical: 33%
Limited importance: 0%

Researchers
Critically important: 12%
Important not critical: 59%
Limited importance: 29%

Clinicians
Critically important: 8%
Important not critical: 75%
Limited importance: 17%

Service-planners/Policymakers
Critically important: 14%
Important not critical: 86%
Limited importance: 0%

Reasons For Scores

Please note that the number in brackets shows the percentage of participants that gave that reason.

Reasons for including:
Important for falls prevention (4%).
May influence disease progression (2%).

Reasons for excluding:
Not shown to be a falls risk factor is research (2%).

Flexibility

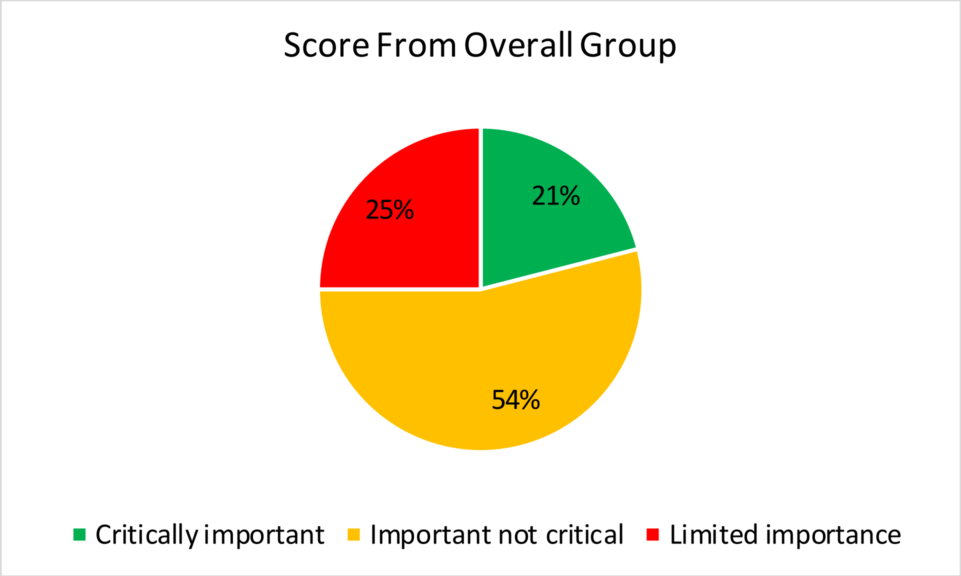

Scores From Each Stakeholder Group

Patients
Critically important: 50%
Important not critical: 50%
Limited importance: 0%

Researchers
Critically important: 6%
Important not critical: 47%
Limited importance: 47%

Clinicians
Critically important: 8%
Important not critical: 59%
Limited importance: 33%

Service-planners/Policymakers
Critically important: 29%
Important not critical: 71%
Limited importance: 0%

Reasons For Scores

Please note that the number in brackets shows the percentage of participants that gave that reason.

<u>Reasons for including:</u>
Important for strategies to prevent falls (6%).
Important for maintaining balance (2%).

<u>Reasons for excluding:</u>
Not related to the primary goal of the intervention (2%).
Strength would be more important (2%).

Fear of falling

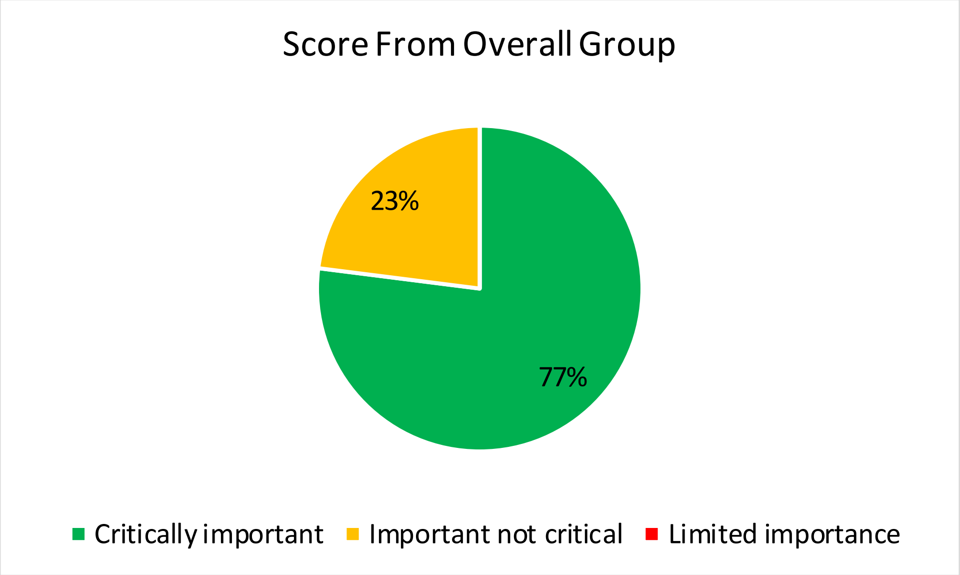

Scores From Each Stakeholder Group

Patients
Critically important: 67%
Important not critical: 33%
Limited importance: 0%

Researchers
Critically important: 76%
Important not critical: 24%
Limited importance: 0%

Clinicians
Critically important: 83%
Important not critical: 17%
Limited importance: 0%

Service-planners/Policymakers
Critically important: 86%
Important not critical: 14%
Limited importance: 0%

Reasons For Scores

Please note that the number in brackets shows the percentage of participants that gave that reason.

Reasons for including:
May increase risk of falls (6%).
Often results in patients reducing their activity (4%).
Enables positive risk taking (2%).
Fear of falling limits balance reactions and progression (2%).

Of note, one participant who scored this outcome as critically important discussed how fear of falling may increase for some, but in parallel with self-management strategies, and so should not be evaluated in isolation.

Reasons for excluding:
Does not have a linear association with falls (2%).

Freezing of gait

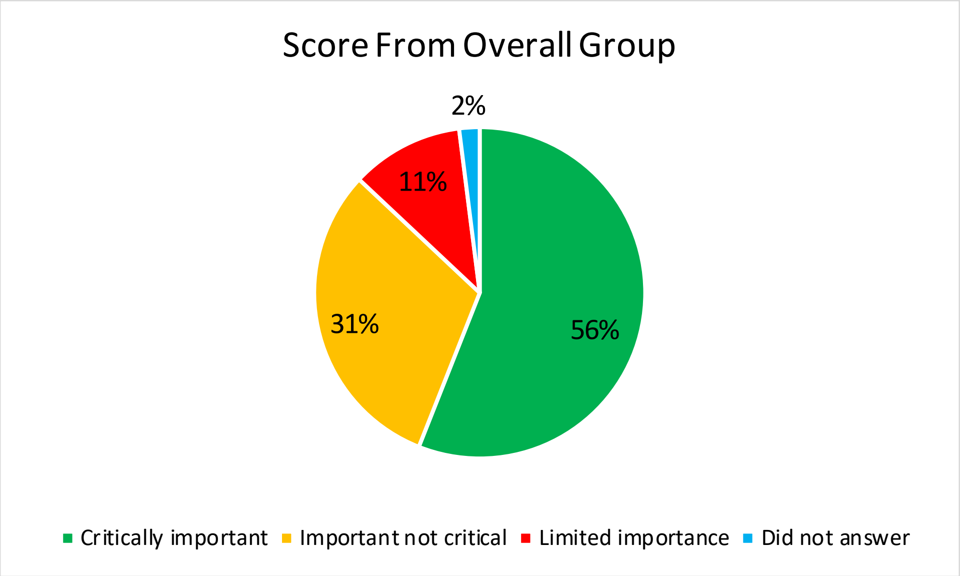

Scores From Each Stakeholder Group

Patients
Critically important: 59%
Important not critical: 25%
Limited importance: 8%
Did not answer: 8%

Researchers
Critically important: 35%
Important not critical: 41%
Limited importance: 24%

Clinicians
Critically important: 67%
Important not critical: 33%
Limited importance: 0%

Service-planners/Policymakers
Critically important: 86%
Important not critical: 14%
Limited importance: 0%

Reasons For Scores

Please note that the number in brackets shows the percentage of participants that gave that reason.

Reasons for including:
Could increase falls risk (13%).

Reasons for excluding:
Not relevant to all interventions only those that aim to prevent falls by improving freezing of gait (4%).
Do not expect freezing of gait to change with a falls prevention intervention (4%).
Better to show the impact on function rather than as a symptom (2%).
May be more appropriate as a descriptor of the sample rather than an outcome (2%).

Knowledge of how to get up from the floor after a fall

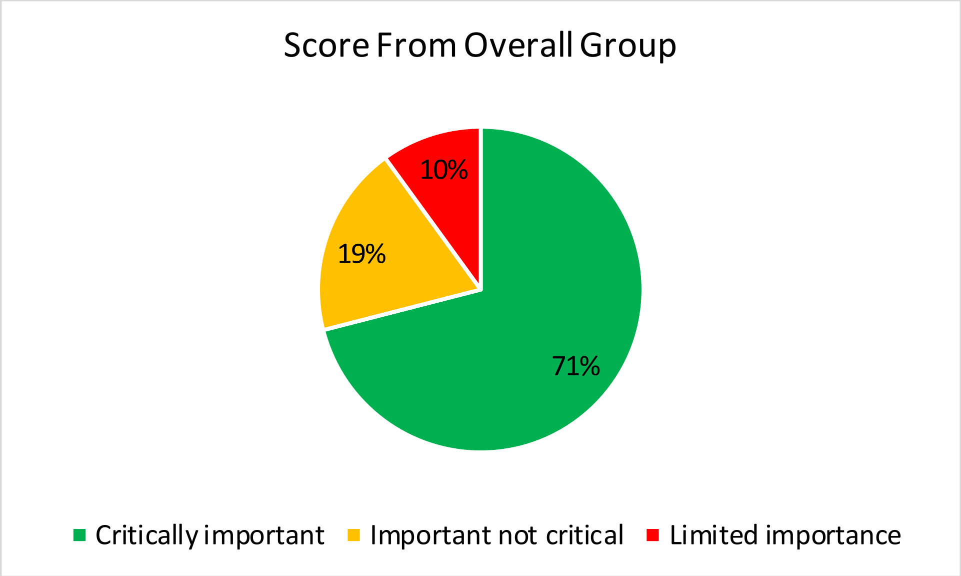

Scores From Each Stakeholder Group

Patients
Critically important: 84%
Important not critical: 8%
Limited importance: 8%

Researchers
Critically important: 52%
Important not critical: 24%
Limited importance: 24%

Clinicians
Critically important: 67%
Important not critical: 33%
Limited importance: 0%

Service-planners/Policymakers
Critically important: 100%
Important not critical: 0%
Limited importance: 0%

Reasons For Scores

Please note that the number in brackets shows the percentage of participants that gave that reason.

Reasons for including:
Lying on floor may cause further complications (2%).
Enables positive risk taking (2%).
In some research, has come out as number one factor for patients (2%).
Evidence suggests that ability to get up from floor reduces fear of falling (2%).
May promote independence (2%).

Reasons for excluding:
Not relevant to all interventions only those where the intervention includes elements about getting up safely after a fall (4%).
Strategy not feasible for all patients as it depends on disease severity and baseline function (2%).
Fall management rather than prevention (2%).

Knowledge of how to fall

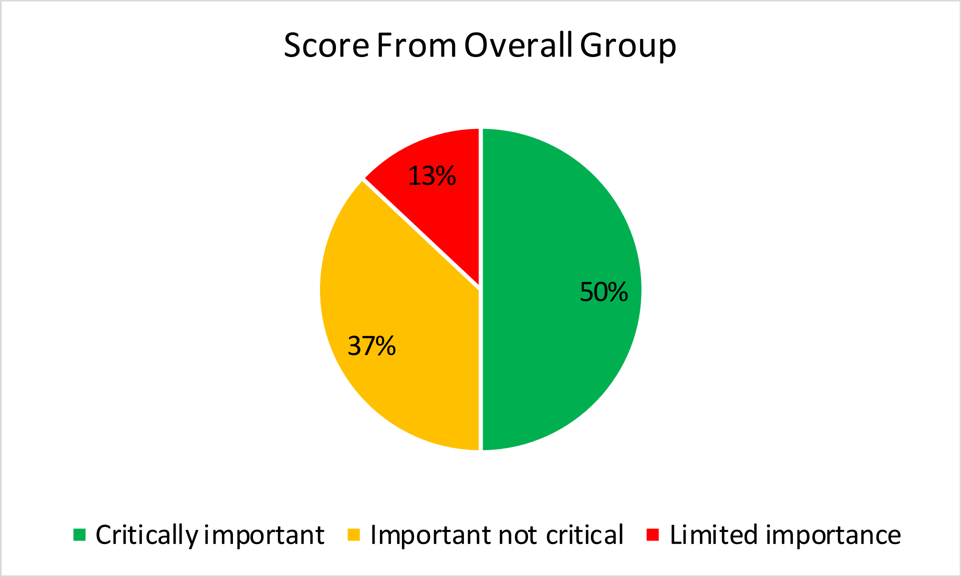

Scores From Each Stakeholder Group

Patients
Critically important: 75%
Important not critical: 17%
Limited importance: 8%

Researchers
Critically important: 35%
Important not critical: 35%
Limited importance: 30%

Clinicians
Critically important: 33%
Important not critical: 67%
Limited importance: 0%

Service-planners/Policymakers
Critically important: 71%
Important not critical: 29%
Limited importance: 0%

Reasons For Scores

Please note that the number in brackets shows the percentage of participants that gave that reason.

Reasons for including:
Enables positive risk taking (2%).
In some research, has come out as number one factor for patients (2%).

Of note, some participants who scored this outcome as critically important highlighted potential issues with this outcome including a lack of evidence supporting this concept (2%) and difficulty with measuring this outcome (2%).

Reasons for excluding:
Not relevant to all interventions only those where the intervention includes elements about how to fall (2%).
Not something all patients want to know (2%).
Fall management rather than prevention (2%).

Ability to independently perform activities of daily living

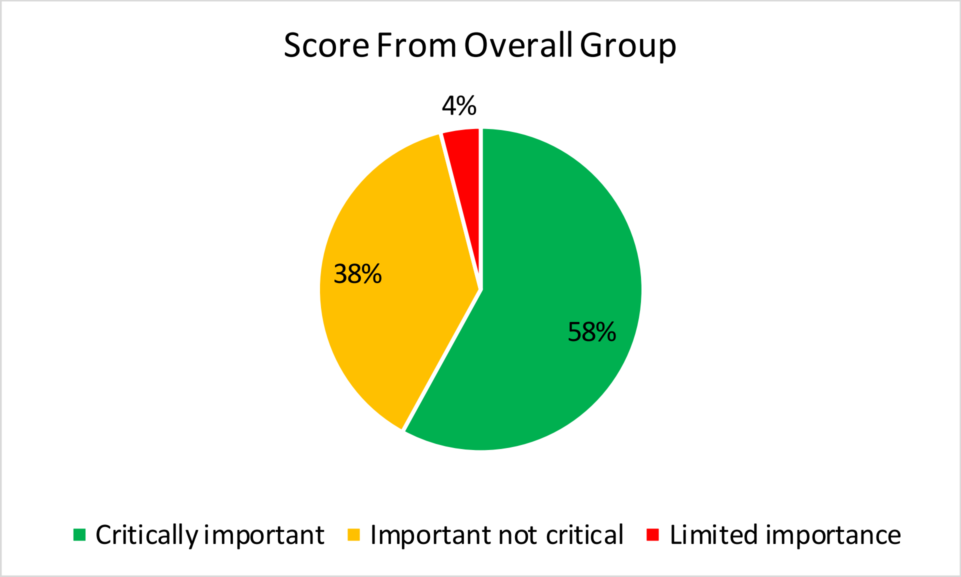

Scores From Each Stakeholder Group

Patients
Critically important: 75%
Important not critical: 25%
Limited importance: 0%

Researchers
Critically important: 47%
Important not critical: 47%
Limited importance: 6%

Clinicians
Critically important: 50%
Important not critical: 42%
Limited importance: 8%

Service-planners/Policymakers
Critically important: 71%
Important not critical: 29%
Limited importance: 0%

Reasons For Scores

Please note that the number in brackets shows the percentage of participants that gave that reason.

Reasons for including:
Often is a key goal for patients (4%).
Enhances self-awareness and motivation (2%).
Important to evaluate from a safety standpoint (2%).

Of note, some participants who scored this outcome as critically important highlighted that independence could only be facilitated to the point that it is reasonably safe (4%).

Reasons for excluding:
May not be physically possible for the patient to complete all activities independently (6%).
Asking for appropriate support may be an effective falls prevention strategy (2%).
Overlap with objective assessment of activities of daily living (2%).
Individuals may not recognise deficits and may be at risk of falling (2%).
Not related to the primary goal of the intervention (2%).

Number of injurious falls

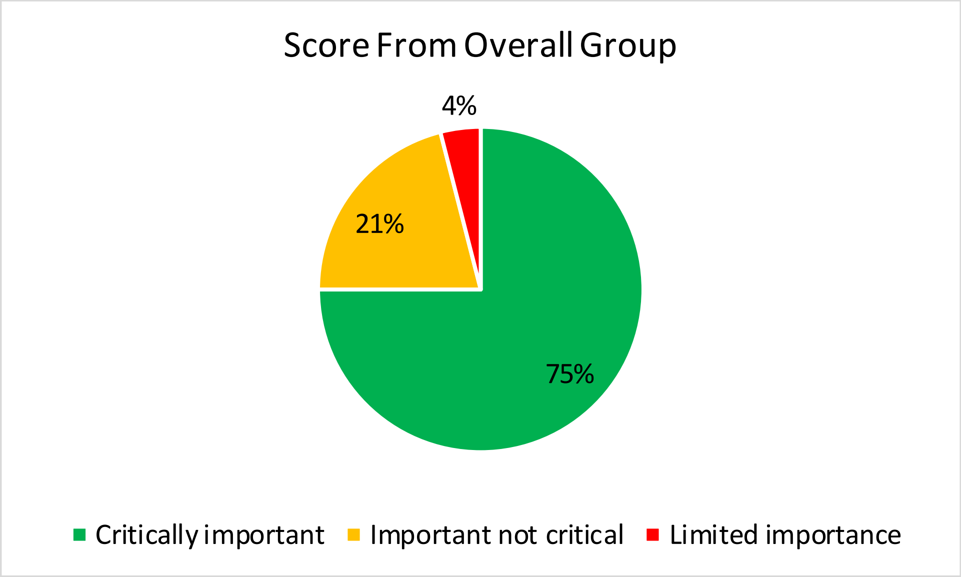

Scores From Each Stakeholder Group

Patients
Critically important: 50%
Important not critical: 33%
Limited importance: 17%

Researchers
Critically important: 94%
Important not critical: 6%
Limited importance: 0%

Clinicians
Critically important: 67%
Important not critical: 33%
Limited importance: 0%

Service-planners/Policymakers
Critically important: 86%
Important not critical: 14%
Limited importance: 0%

Reasons For Scores

Please note that the number in brackets shows the percentage of participants that gave that reason.

Reasons for including:
Injuries result in costs to the healthcare system (4%)
Data may prove useful for meta-analyses (2%).
Falls with harm lead to reduction in function and possible one-year mortality (2%).

Reasons for excluding:
Injuries are rare so most studies will not be powered to provide information on this outcome (2%).

Level of physical activity

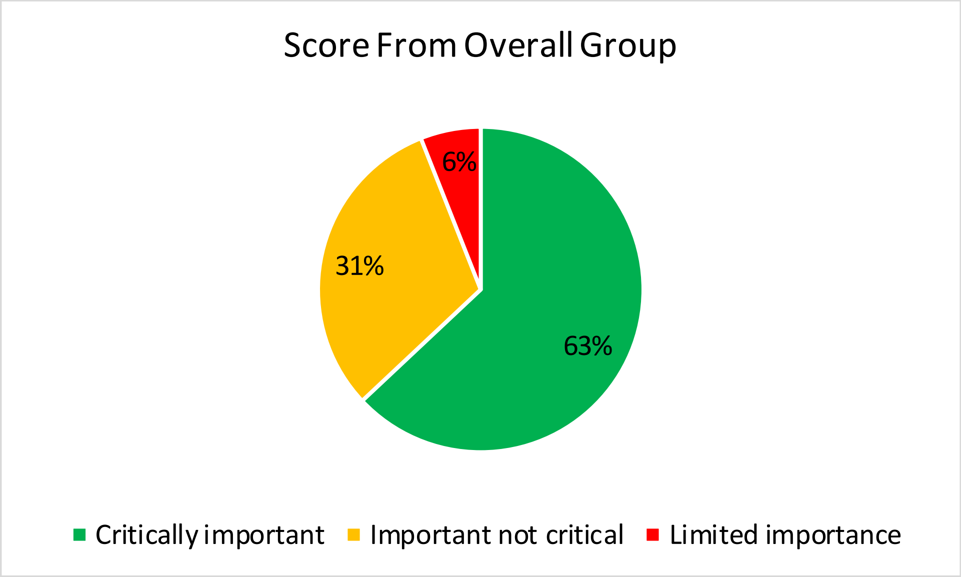

Scores From Each Stakeholder Group

Patients
Critically important: 83%
Important not critical: 17%
Limited importance: 0%

Researchers
Critically important: 53%
Important not critical: 29%
Limited importance: 18%

Clinicians
Critically important: 33%
Important not critical: 67%
Limited importance: 0%

Service-planners/Policymakers
Critically important: 100%
Important not critical: 0%
Limited importance: 0%

Reasons For Scores

Please note that the number in brackets shows the percentage of participants that gave that reason.

<u>Reasons for including:</u>
Important for falls prevention (4%).
Can help to understand fall results in relation to exposure to times when a person is likely to fall (4%).
Linked to participation (2%).

<u>Reasons for excluding:</u>
Not relevant to all interventions only those where the intervention is expected to increase physical activity by reducing fear of falling/reducing activities avoided due to fear of falling (2%).

Lower limb strength

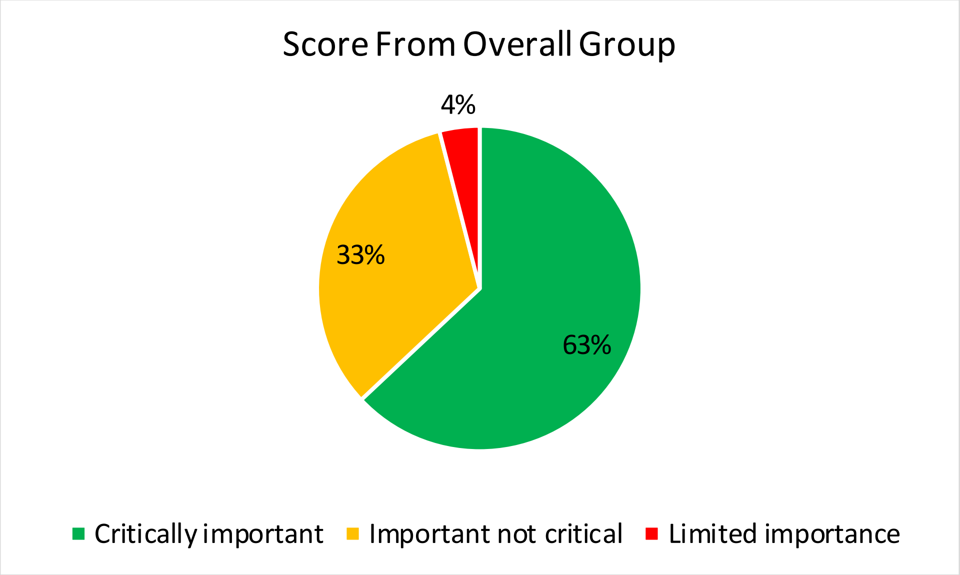

Scores From Each Stakeholder Group

Patients
Critically important: 75%
Important not critical: 25%
Limited importance: 0%

Researchers
Critically important: 47%
Important not critical: 41%
Limited importance: 12%

Clinicians
Critically important: 50%
Important not critical: 50%
Limited importance: 0%

Service-planners/Policymakers
Critically important: 100%
Important not critical: 0%
Limited importance: 0%

Reasons For Scores

Please note that the number in brackets shows the percentage of participants that gave that reason.

Reasons for including:
Important for maintaining balance (2%).
Enables positive risk taking (2%).
Necessary to be able to get up from the floor (2%).
Important for falls prevention (2%).

Reasons for excluding:
Not relevant to all interventions only those where the goal is to prevent falls by improving lower limb strength (2%).
Unsure of evidence that lower limb strength is a falls risk factor for people with each of the three conditions (2%).

Number of falls resulting in a long lie

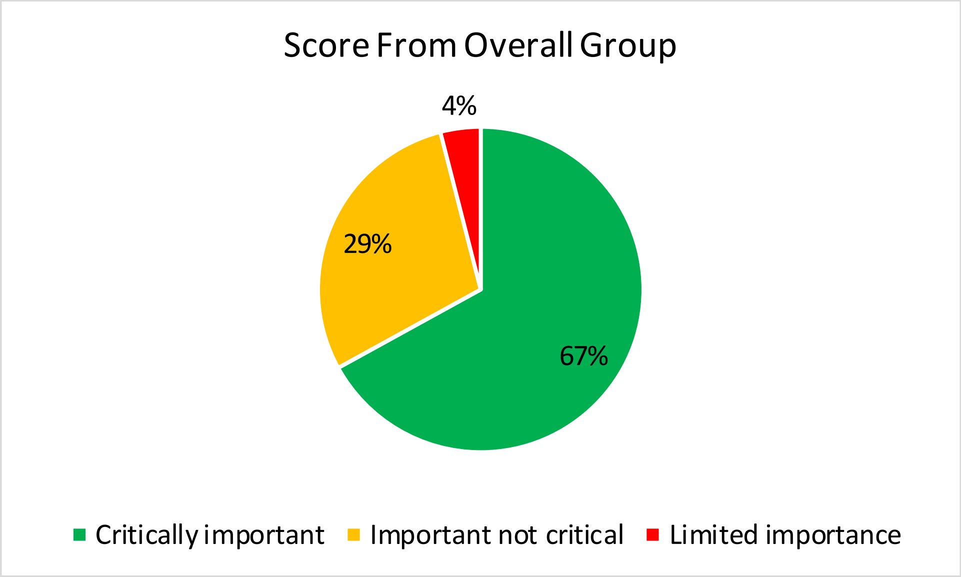

Scores From Each Stakeholder Group

Patients
Critically important: 66%
Important not critical: 17%
Limited importance: 17%

Researchers
Critically important: 59%
Important not critical: 41%
Limited importance: 0%

Clinicians
Critically important: 58%
Important not critical: 42%
Limited importance: 0%

Service-planners/Policymakers
Critically important: 100%
Important not critical: 0%
Limited importance: 0%

Reasons For Scores

Please note that the number in brackets shows the percentage of participants that gave that reason.

Reasons for including:
Can have serious injury and psychological consequences (2%).
Associated with increased morbidity (2%).
May demonstrate difficulty with independent living and possible increased care needs (2%).

Reasons for excluding:
Quite rare events (4%).
Not relevant to all interventions only those where the intervention would improve ability to get up after a fall (2%).

Number of near falls

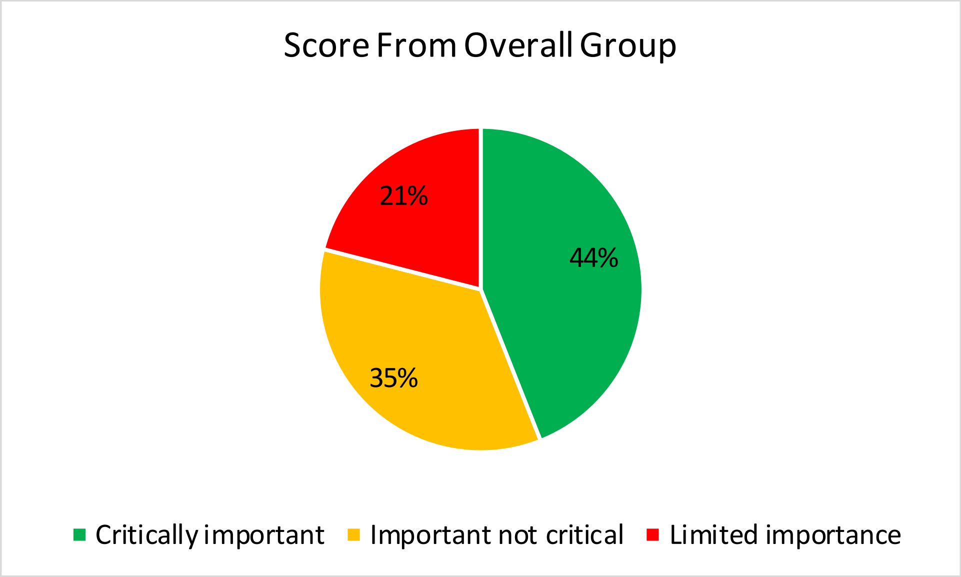

Scores From Each Stakeholder Group

Patients
Critically important: 67%
Important not critical: 25%
Limited importance: 8%

Researchers
Critically important: 41%
Important not critical: 18%
Limited importance: 41%

Clinicians
Critically important: 17%
Important not critical: 66%
Limited importance: 17%

Service-planners/Policymakers
Critically important: 57%
Important not critical: 43%
Limited importance: 0%

Reasons For Scores

Please note that the number in brackets shows the percentage of participants that gave that reason.

Reasons for including:
Demonstrate a patient's balance ability/capacity (4%).
It could have been a fall (2%).

Reasons for excluding:
Records of near falls are unreliable (8%).
Unclear if it should be viewed as a positive outcome indicating balance recovery/falls averted or a negative outcome indicating risk (6%).
Difficult to define (6%).

Total number of falls

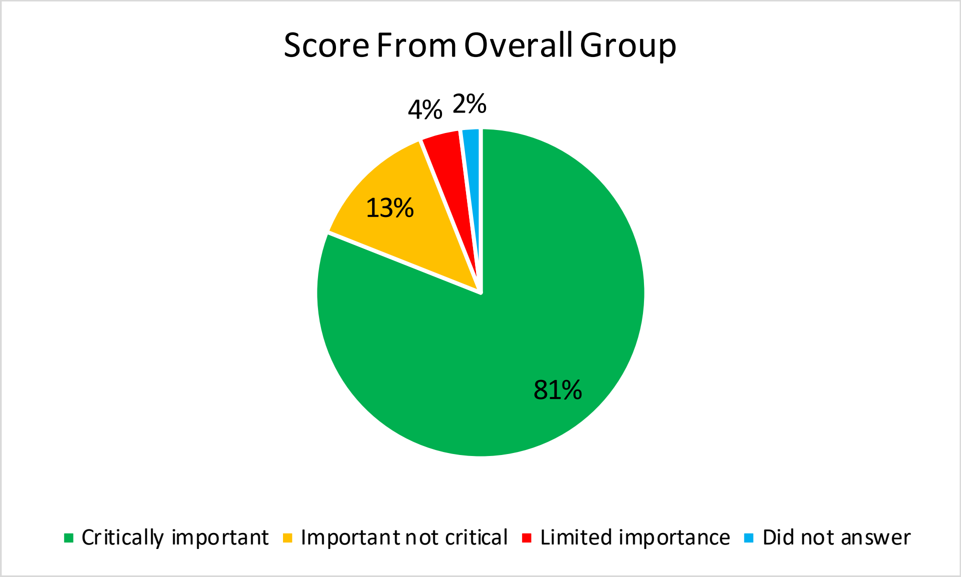

Scores From Each Stakeholder Group

Patients
Critically important: 84%
Important not critical: 0%
Limited importance: 8%

Researchers
Critically important: 82%
Important not critical: 12%
Limited importance: 6%

Clinicians
Critically important: 75%
Important not critical: 25%
Limited importance: 0%

Service-planners/Policymakers
Critically important: 86%
Important not critical: 14%
Limited importance: 0%

Reasons For Scores

Please note that the number in brackets shows the percentage of participants that gave that reason.

<u>Reasons for excluding:</u>
Difficult to accurately record (4%) especially if based on retrospective self-report (2%).
It might only take one fall to be fatal (2%).

Objectively assessed ability to perform activities of daily living

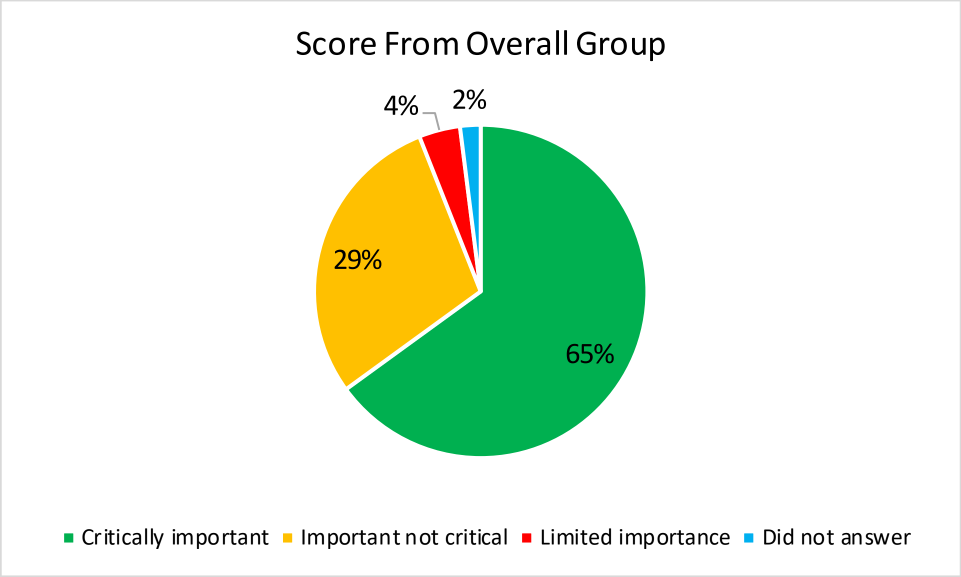

Scores From Each Stakeholder Group

Patients
Critically important: 75%
Important not critical: 17%
Limited importance: 0%
Did not answer: 8%

Researchers
Critically important: 29%
Important not critical: 65%
Limited importance: 6%

Clinicians
Critically important: 50%
Important not critical: 42%
Limited importance: 8%

Service-planners/Policymakers
Critically important: 86%
Important not critical: 14%
Limited importance: 0%

Reasons For Scores

Please note that the number in brackets shows the percentage of participants that gave that reason.

Reasons for including:
Objective assessment is more reliable (6%).
Falls can occur during routine (unavoidable) activities of daily living (2%).
Important that falls are not reduced by a patient simply limiting activity/participation (2%).

Reasons for excluding:
Difficult outcome to measure (6%) – physical activity is an easier outcome to assess and would incorporate activities of daily living into movement count (2%).
Patient's perception of their ability to perform activities of daily living is a more important outcome (4%).
It is activity dependent – if related to gait or balance it will be important but not if related to upper limb activities (2%).
Not related to the primary goal of the intervention (2%).

Objectively assessed mobility

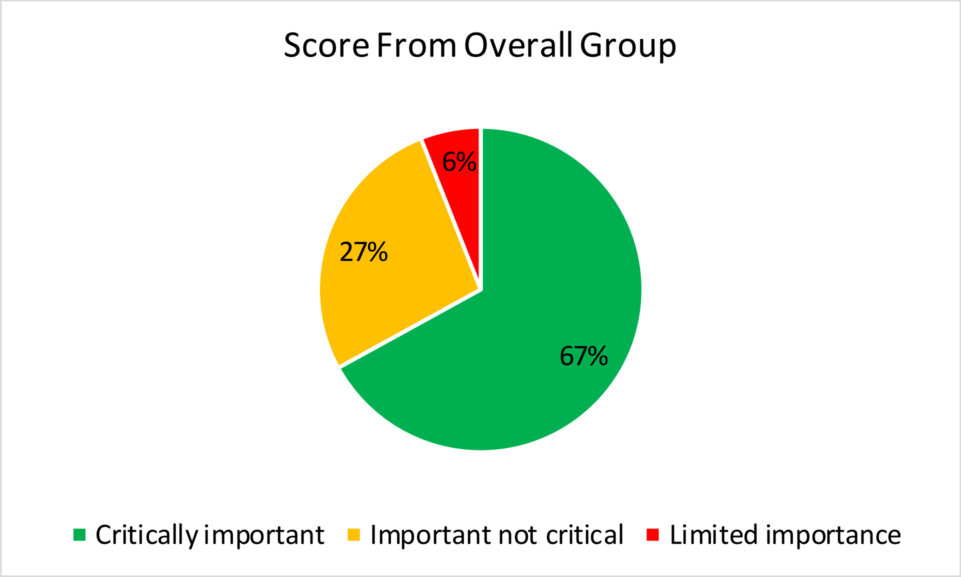

Scores From Each Stakeholder Group

Patients
Critically important: 92%
Important not critical: 8%
Limited importance: 0%

Researchers
Critically important: 47%
Important not critical: 35%
Limited importance: 18%

Clinicians
Critically important: 58%
Important not critical: 42%
Limited importance: 0%

Service-planners/Policymakers
Critically important: 86%
Important not critical: 14%
Limited importance: 0%

Reasons For Scores

Please note that the number in brackets shows the percentage of participants that gave that reason.

<u>Reasons for including:</u>
Efficient gait pattern minimises falls risk (2%).

<u>Reasons for excluding:</u>
Not related to the primary goal of the intervention (2%).

Pain

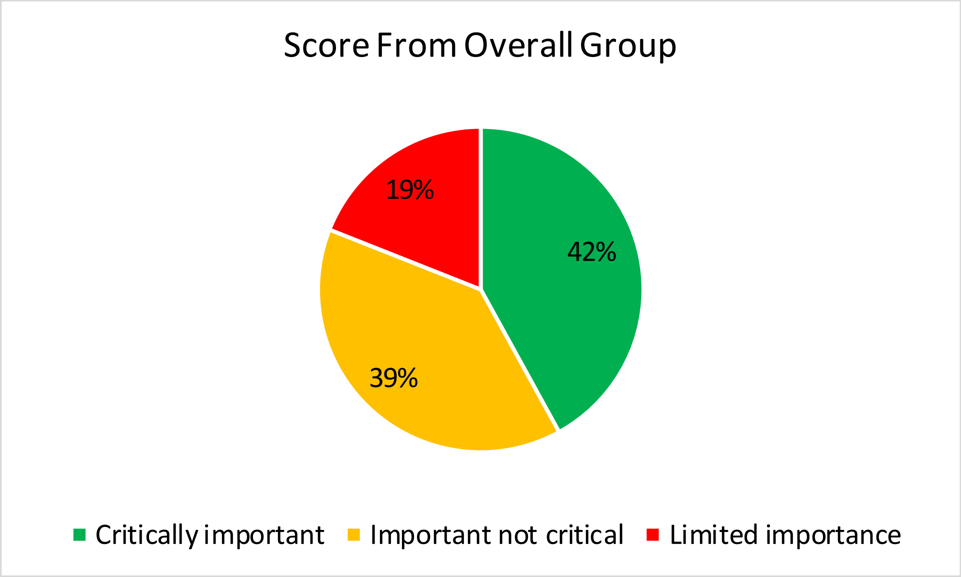

Scores From Each Stakeholder Group

Patients
Critically important: 67%
Important not critical: 33%
Limited importance: 0%

Researchers
Critically important: 18%
Important not critical: 29%
Limited importance: 53%

Clinicians
Critically important: 25%
Important not critical: 75%
Limited importance: 0%

Service-planners/Policymakers
Critically important: 86%
Important not critical: 14%
Limited importance: 0%

Reasons For Scores

Please note that the number in brackets shows the percentage of participants that gave that reason.

<u>Reasons for including:</u>
Could contribute to falling (2%).

<u>Reasons for excluding:</u>
Not relevant to the primary aim of the intervention – falls prevention (4%).
May not be relevant to all patients (4%) but should be considered if pain impacts on fear of falling/mobility (2%).

Peer support

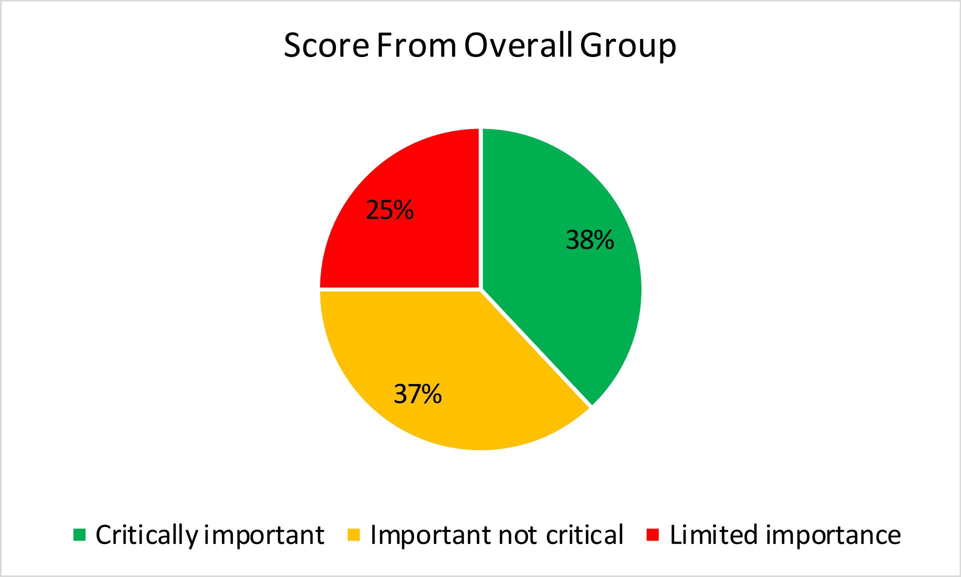

Scores From Each Stakeholder Group

Patients
Critically important: 67%
Important not critical: 25%
Limited importance: 8%

Researchers
Critically important: 12%
Important not critical: 23%
Limited importance: 65%

Clinicians
Critically important: 25%
Important not critical: 75%
Limited importance: 0%

Service-planners/Policymakers
Critically important: 71%
Important not critical: 29%
Limited importance: 0%

Reasons For Scores

Please note that the number in brackets shows the percentage of participants that gave that reason.

<u>Reasons for including:</u>
Keeps interventions sustainable (2%).
Peer support may increase outcomes (2%).

<u>Reasons for excluding:</u>
Peer support should be considered a process-oriented factor rather than an outcome (4%).
Not relevant to the primary aim of the intervention – falls prevention (2%).
Some people may not have peer support (2%).

Perceived control of falls

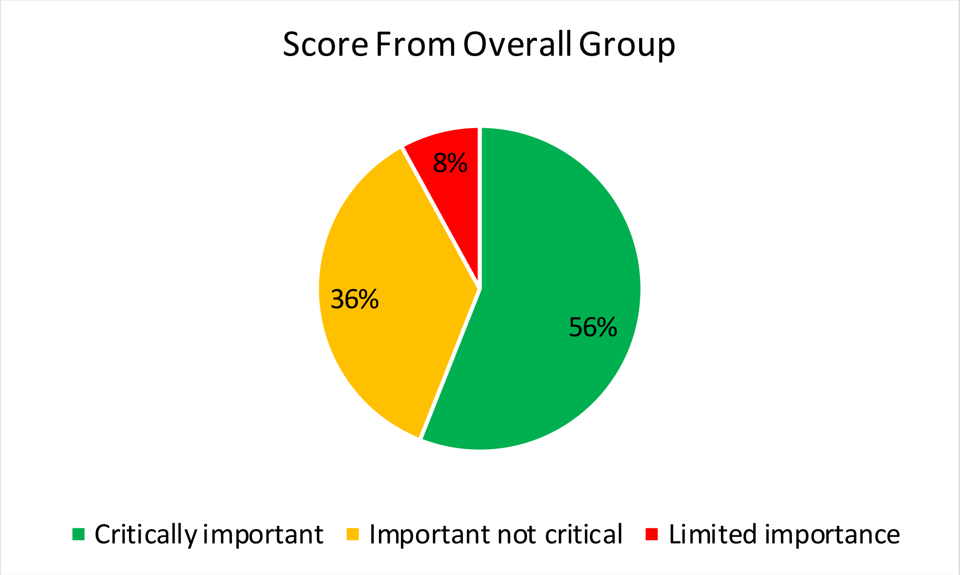

Scores From Each Stakeholder Group

Patients
Critically important: 58%
Important not critical: 34%
Limited importance: 8%

Researchers
Critically important: 59%
Important not critical: 23%
Limited importance: 18%

Clinicians
Critically important: 42%
Important not critical: 58%
Limited importance: 0%

Service-planners/Policymakers
Critically important: 71%
Important not critical: 29%
Limited importance: 0%

Reasons For Scores

Please note that the number in brackets shows the percentage of participants that gave that reason.

Reasons for including:
Enables positive risk taking (2%).
Could reduce fear of falling (2%).

Reasons for excluding:
This is a vague, poorly-defined outcome (4%).
Subjective measures can be inaccurate (2%).

Self-perceived impact on carer/family

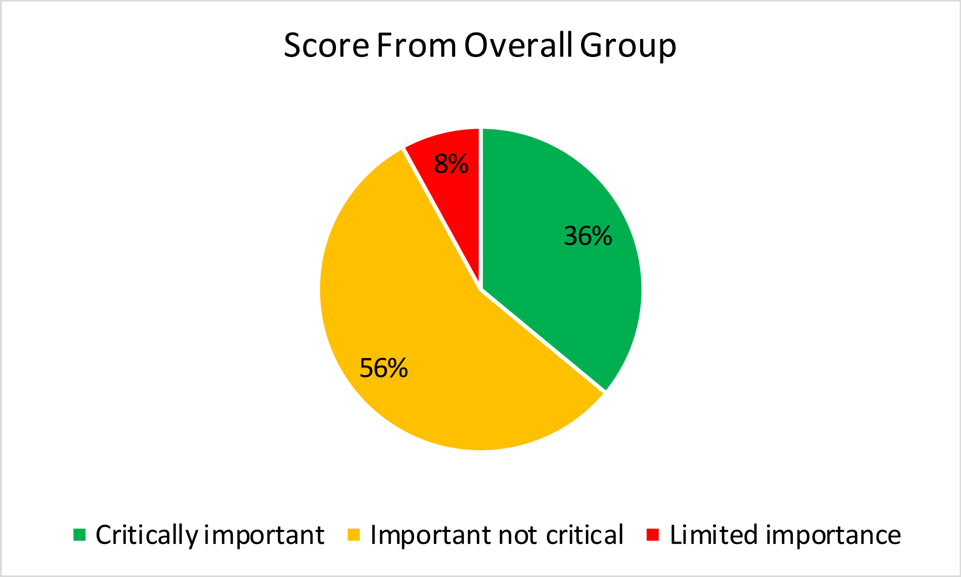

Scores From Each Stakeholder Group

Patients
Critically important: 67%
Important not critical: 33%
Limited importance: 0%

Researchers
Critically important: 12%
Important not critical: 70%
Limited importance: 18%

Clinicians
Critically important: 25%
Important not critical: 67%
Limited importance: 8%

Service-planners/Policymakers
Critically important: 57%
Important not critical: 43%
Limited importance: 0%

Reasons For Scores

Please note that the number in brackets shows the percentage of participants that gave that reason.

Reasons for including:
To ensure sufficient mutual supports are available (2%).

Reasons for excluding:
Not related to the primary goal of the intervention (2%).
Families may not be aware of the risk of falls (2%).
This may be more of a motivating factor than an outcome (2%).

Quality of life

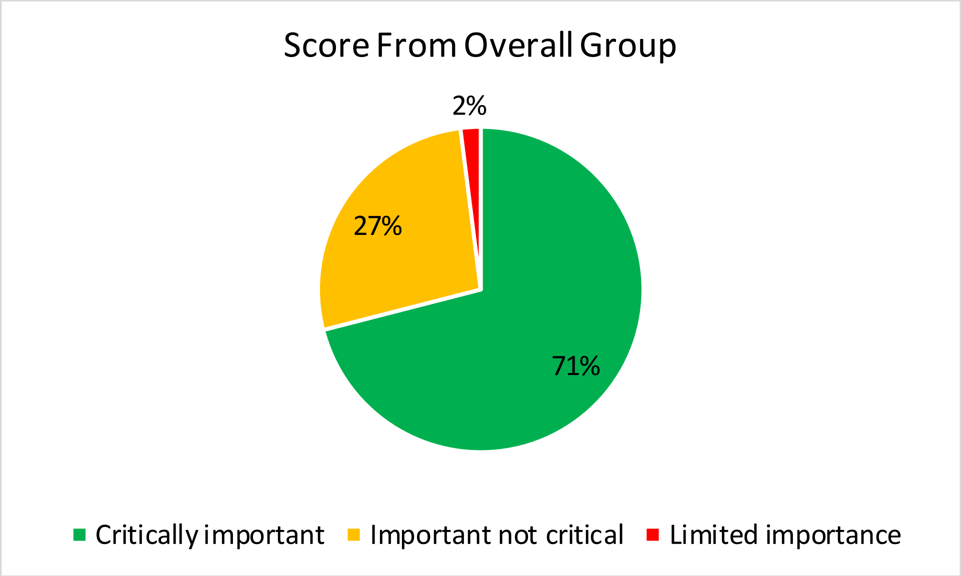

Scores From Each Stakeholder Group

Patients
Critically important: 100%
Important not critical: 0%
Limited importance: 0%

Researchers
Critically important: 53%
Important not critical: 41%
Limited importance: 6%

Clinicians
Critically important: 58%
Important not critical: 42%
Limited importance: 0%

Service-planners/Policymakers
Critically important: 86%
Important not critical: 14%
Limited importance: 0%

Number of recurrent fallers

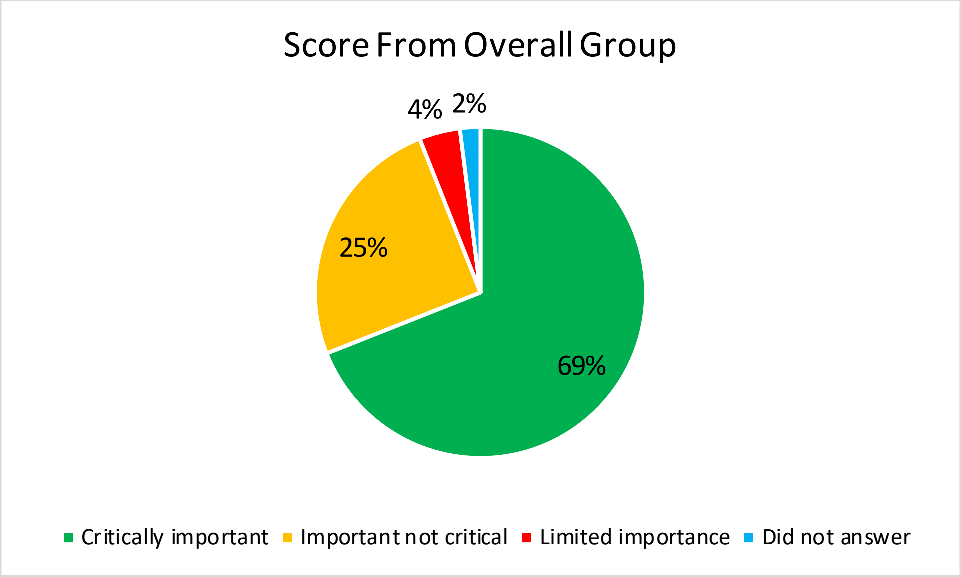

Scores From Each Stakeholder Group

Patients
Critically important: 50%
Important not critical: 34%
Limited importance: 8%
Did not answer: 8%

Researchers
Critically important: 82%
Important not critical: 12%
Limited importance: 6%

Clinicians
Critically important: 58%
Important not critical: 42%
Limited importance: 0%

Service-planners/Policymakers
Critically important: 86%
Important not critical: 14%
Limited importance: 0%

Reasons For Scores

Please note that the number in brackets shows the percentage of participants that gave that reason.

Reasons for excluding:
Fall rates capture this better/should be more sensitive (4%).

Self-efficacy

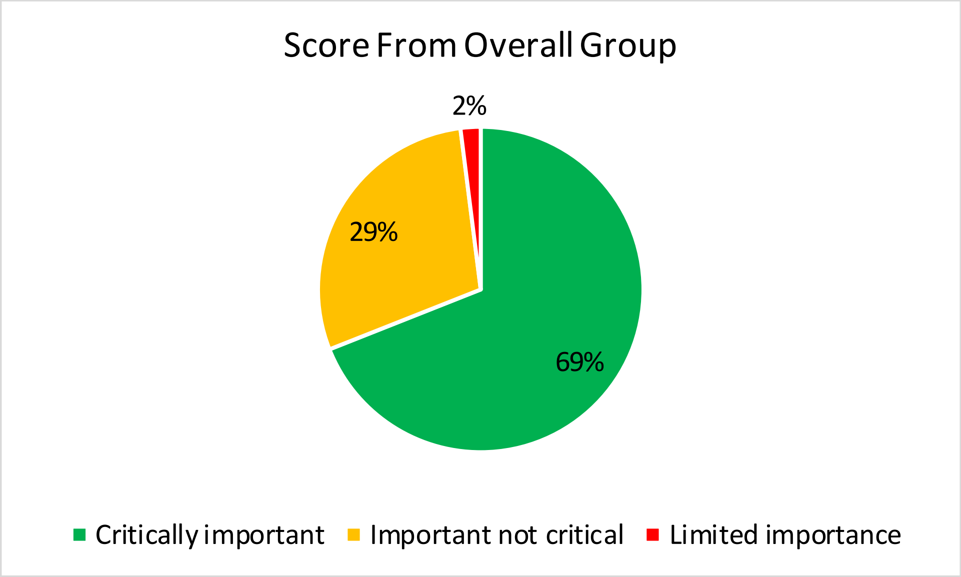

Scores From Each Stakeholder Group

Patients
Critically important: 83%
Important not critical: 17%
Limited importance: 0%

Researchers
Critically important: 53%
Important not critical: 41%
Limited importance: 6%

Clinicians
Critically important: 67%
Important not critical: 33%
Limited importance: 0%

Service-planners/Policymakers
Critically important: 86%
Important not critical: 14%
Limited importance: 0%

Reasons For Scores

Please note that the number in brackets shows the percentage of participants that gave that reason.

<u>Reasons for including:</u>
Likely to influence engagement with intervention/completion of falls prevention strategies (10%).
Enhances motivation (4%), self-awareness (2%), and self-confidence (2%).
Necessary for change (2%).

<u>Reasons for excluding:</u>
If judgement is affected, individuals can be reckless (2%).
Concept of outcome is vague (2%).
Outcome is too subjective (2%).
Not related to the primary goal of the intervention (2%).

Falls self-management skills

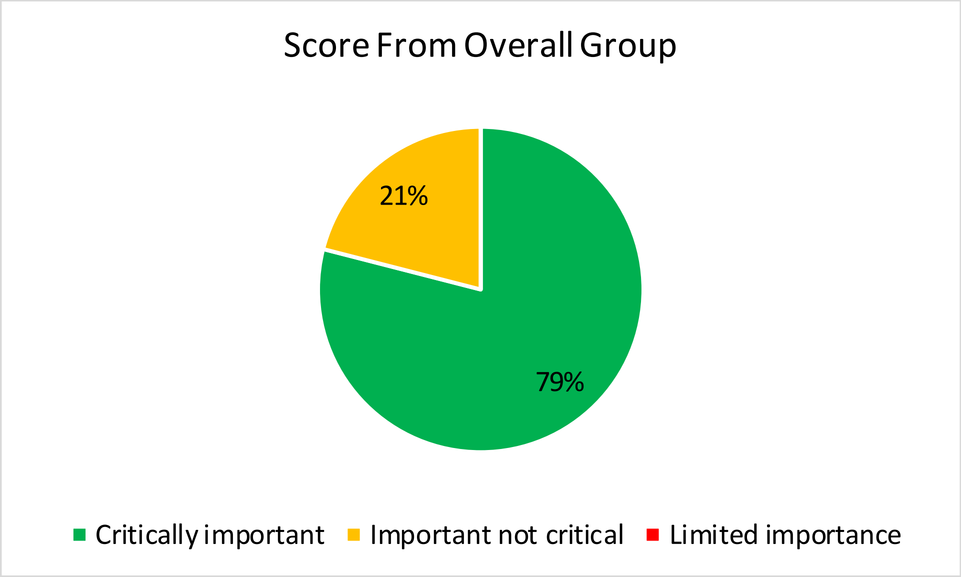

Scores From Each Stakeholder Group

Patients
Critically important: 92%
Important not critical: 8%
Limited importance: 0%

Researchers
Critically important: 76%
Important not critical: 24%
Limited importance: 0%

Clinicians
Critically important: 58%
Important not critical: 42%
Limited importance: 0%

Service-planners/Policymakers
Critically important: 100%
Important not critical: 0%
Limited importance: 0%

Reasons For Scores

Please note that the number in brackets shows the percentage of participants that gave that reason.

Reasons for including:
Encourages independence (6%).
Education is useful in preventing falls (2%).

Reasons for excluding:
Outcome is not relevant to all interventions only those where the goal is to prevent falls through self-management skills (2%).

Self-reported ability to perform activities of daily living

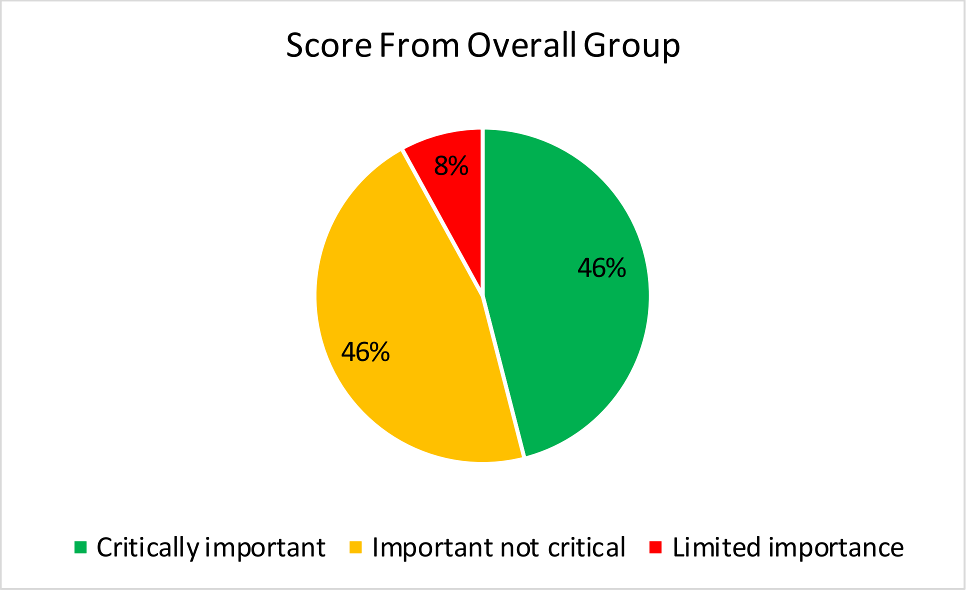

Scores From Each Stakeholder Group

Patients
Critically important: 58%
Important not critical: 42%
Limited importance: 0%

Researchers
Critically important: 41%
Important not critical: 53%
Limited importance: 6%

Clinicians
Critically important: 50%
Important not critical: 25%
Limited importance: 25%

Service-planners/Policymakers
Critically important: 29%
Important not critical: 71%
Limited importance: 0%

Reasons For Scores

Please note that the number in brackets shows the percentage of participants that gave that reason.

<u>Reasons for including:</u>
Important to balance self-reported and objective outcomes (4%).
Falls can occur during routine (unavoidable) activities of daily living (2%).
Enhances self-awareness and motivation (2%).
Person-reported outcomes are key indicators in health policy (2%).
Fear of falling can limit activities of daily living (2%).

<u>Reasons for excluding:</u>
Can be inaccurate as scores can be affected by factors such as insight or cognition (15%).
Not related to the primary goal of the intervention (2%).
Patients can find it hard to rate (2%).
It is activity dependent – if related to gait or balance it will be important but not if related to upper limb activities (2%).

Self-reported mobility

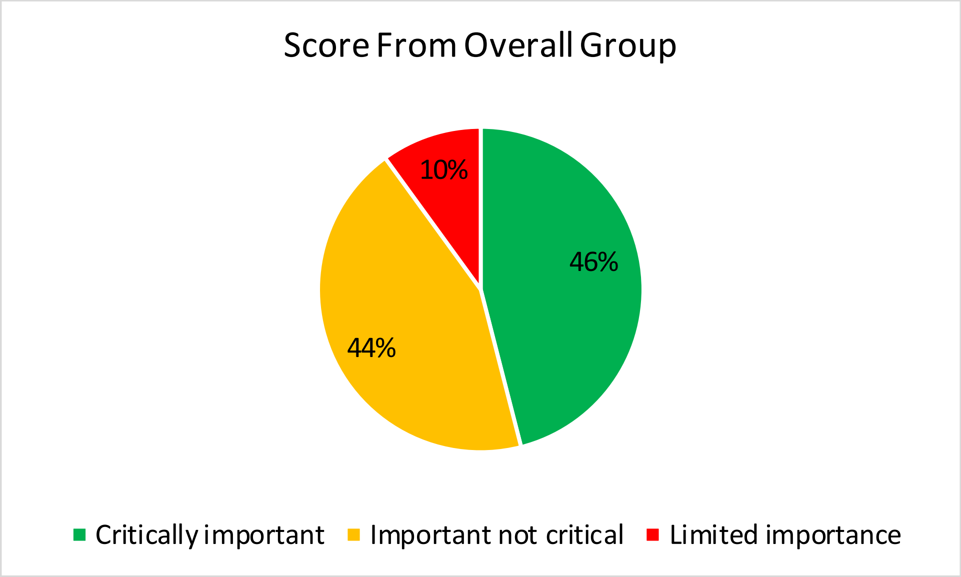

Scores From Each Stakeholder Group

Patients
Critically important: 58%
Important not critical: 42%
Limited importance: 0%

Researchers
Critically important: 35%
Important not critical: 41%
Limited importance: 24%

Clinicians
Critically important: 42%
Important not critical: 50%
Limited importance: 8%

Service-planners/Policymakers
Critically important: 57%
Important not critical: 43%
Limited importance: 0%

Reasons For Scores

Please note that the number in brackets shows the percentage of participants that gave that reason.

Reasons for excluding:
Not related to the primary goal of the intervention (2%).
Patient may not recognise deficits (2%).

Sleep quality

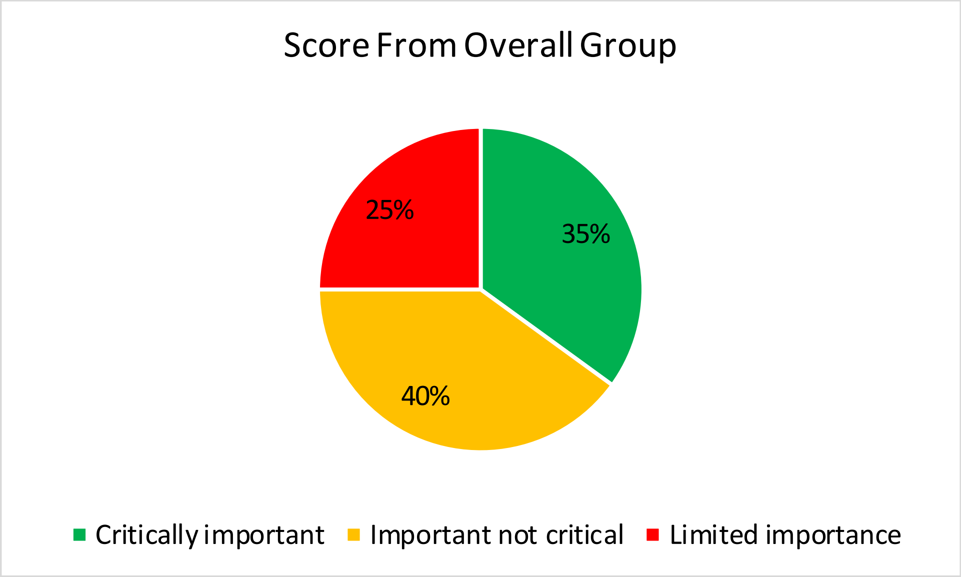

Scores From Each Stakeholder Group

Patients
Critically important: 83%
Important not critical: 17%
Limited importance: 0%

Researchers
Critically important: 6%
Important not critical: 41%
Limited importance: 53%

Clinicians
Critically important: 17%
Important not critical: 66%
Limited importance: 17%

Service-planners/Policymakers
Critically important: 57%
Important not critical: 29%
Limited importance: 14%

Reasons For Scores

Please note that the number in brackets shows the percentage of participants that gave that reason.

Reasons for including:
Tiredness impacts activity (4%).
Tiredness could contribute to falls (2%).

Reasons for excluding:
Not related to the primary goal of the intervention (2%).

Ability to engage in social activities

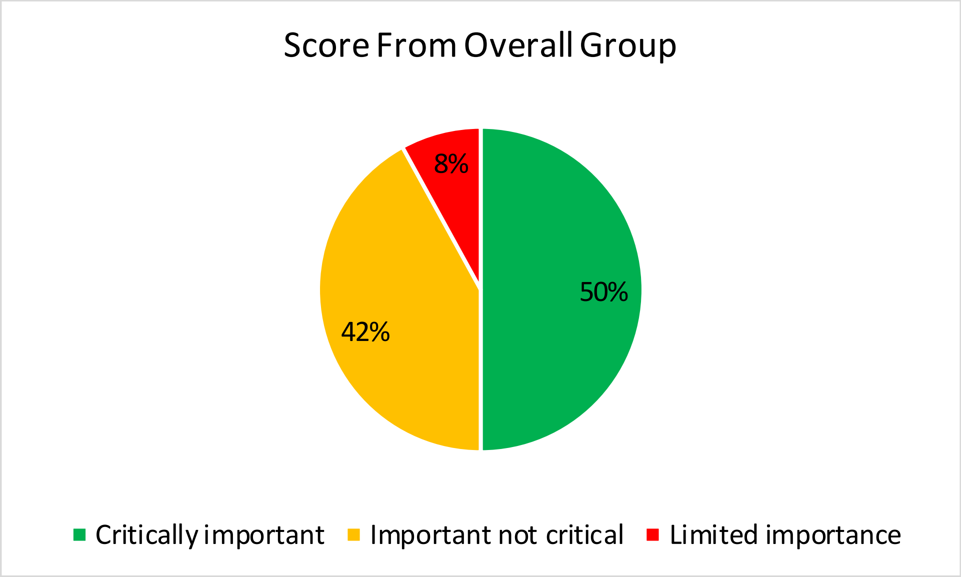

Scores From Each Stakeholder Group

Patients
Critically important: 59%
Important not critical: 33%
Limited importance: 8%

Researchers
Critically important: 47%
Important not critical: 41%
Limited importance: 12%

Clinicians
Critically important: 42%
Important not critical: 50%
Limited importance: 8%

Service-planners/Policymakers
Critically important: 57%
Important not critical: 43%
Limited importance: 0%

Reasons For Scores

Please note that the number in brackets shows the percentage of participants that gave that reason.

Reasons for including:
Aligns with patient goals (6%).
Important for functional, physical and cognitive enhancement (2%).
Reduce adverse effects of loneliness and isolation (2%).
Important to translate reduction in falls and fear of falling to participation (2%).

Reasons for excluding:
Individuals may not engage in social activities for reasons not related to falls/condition
Only if safe to return to social engagement (6%).
Depends on level of social engagement of each individual as it may be more of a priority for some than others (2%).
Not related to the primary goal of the intervention (2%).
It is activity dependent as not all activities present a risk of falling (2%).

Static balance

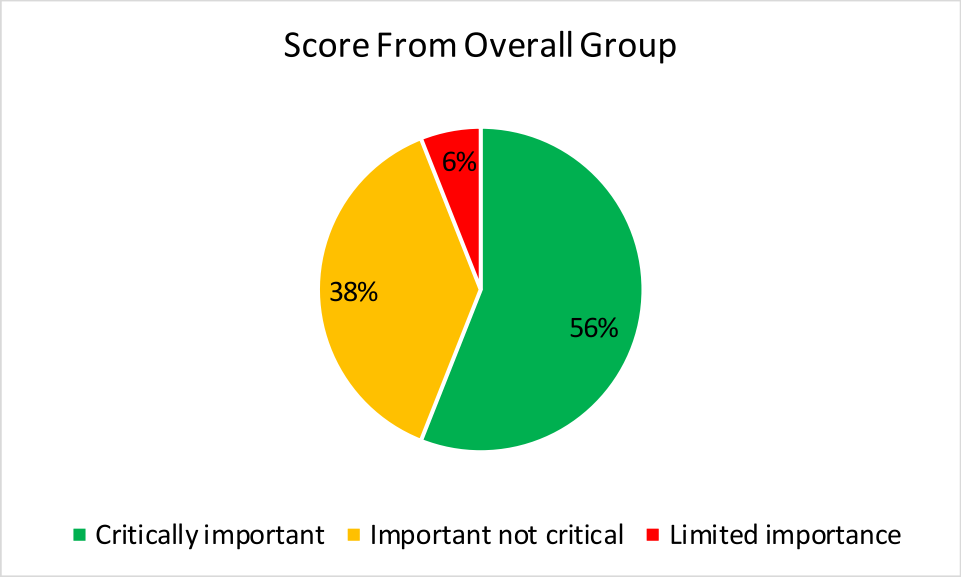

Scores From Each Stakeholder Group

Patients
Critically important: 92%
Important not critical: 8%
Limited importance: 0%

Researchers
Critically important: 23%
Important not critical: 59%
Limited importance: 18%

Clinicians
Critically important: 58%
Important not critical: 42%
Limited importance: 0%

Service-planners/Policymakers
Critically important: 71%
Important not critical: 29%
Limited importance: 0%

Reasons For Scores

Please note that the number in brackets shows the percentage of participants that gave that reason.

<u>Reasons for including:</u>
Essential for safe completion of activities of daily living (4%).
Important for falls prevention (2%).
Can be built upon for functional task retraining (2%).

<u>Reasons for excluding:</u>
Not as important as dynamic balance as falls mostly occur with movement (8%).
Shown to have low correlation with falls risk (2%).
Not relevant to all interventions only those where they goal is to prevent falls by improving balance control (2%).

Stride length

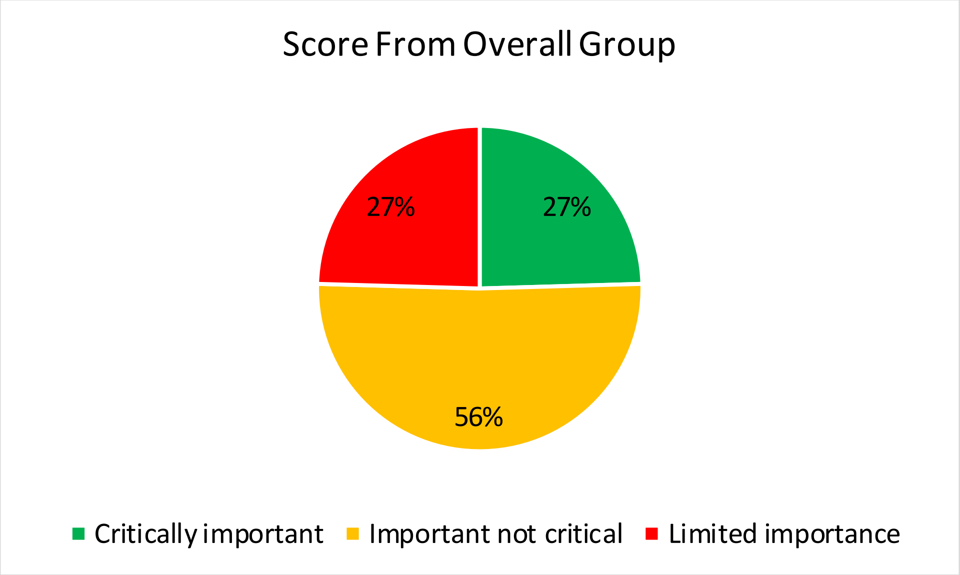

Scores From Each Stakeholder Group

Patients
Critically important: 33%
Important not critical: 67%
Limited importance: 0%

Researchers
Critically important: 12%
Important not critical: 47%
Limited importance: 41%

Clinicians
Critically important: 33%
Important not critical: 59%
Limited importance: 8%

Service-planners/Policymakers
Critically important: 43%
Important not critical: 57%
Limited importance: 0%

Reasons For Scores

Please note that the number in brackets shows the percentage of participants that gave that reason.

Reasons for including:
Important in maintaining balance (2%).

Reasons for excluding:
Not aware of any evidence to measure stride length (2%).
Reduced stride length may be a positive outcome for some patients (2%).
Covered by walking speed (2%).
Not related to the primary goal of the intervention (2%).

Time to first post-intervention fall

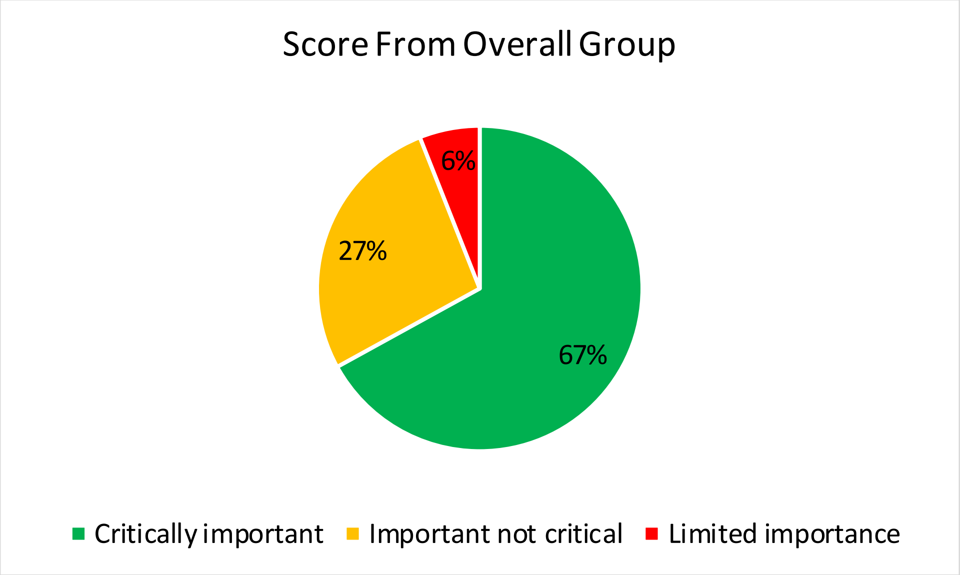

Scores From Each Stakeholder Group

Patients
Critically important: 67%
Important not critical: 25%
Limited importance: 8%

Researchers
Critically important: 71%
Important not critical: 29%
Limited importance: 0%

Clinicians
Critically important: 50%
Important not critical: 42%
Limited importance: 8%

Service-planners/Policymakers
Critically important: 86%
Important not critical: 0%
Limited importance: 14%

Reasons For Scores

Please note that the number in brackets shows the percentage of participants that gave that reason.

Reasons for including:
May indicate how long the treatment effect would last (4%).

Reasons for excluding:
Inferior method of analysis (2%).

Understanding of personal falls risk factors

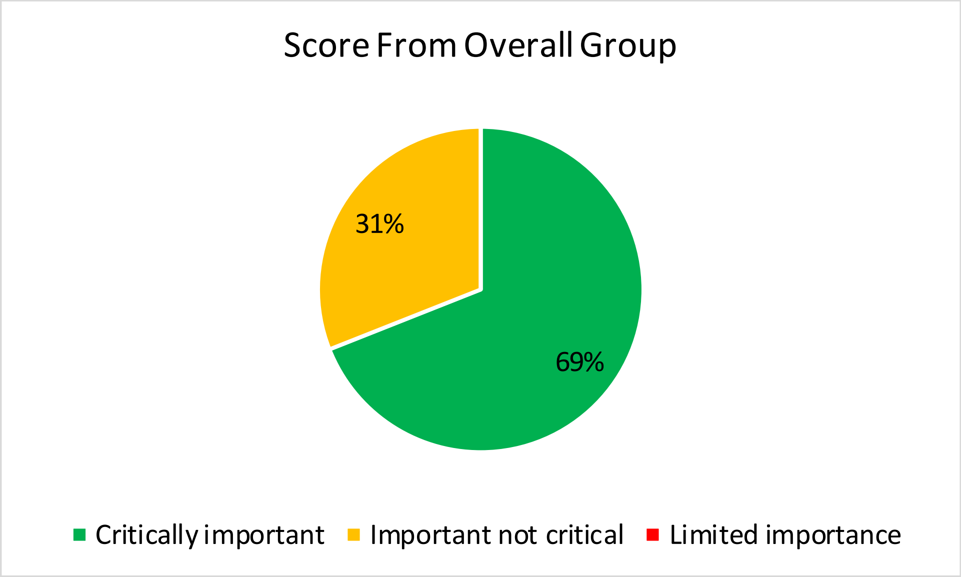

Scores From Each Stakeholder Group

Patients
Critically important: 92%
Important not critical: 8%
Limited importance: 0%

Researchers
Critically important: 59%
Important not critical: 41%
Limited importance: 0%

Clinicians
Critically important: 50%
Important not critical: 50%
Limited importance: 0%

Service-planners/Policymakers
Critically important: 86%
Important not critical: 14%
Limited importance: 0%

Reasons For Scores

Please note that the number in brackets shows the percentage of participants that gave that reason.

<u>Reasons for including:</u>
Assesses understanding and recall of education (6%).
Understanding risk can help patients reduce it (2%).
Enhances self-management (2%).

<u>Reasons for excluding:</u>
Outcome is not relevant to all interventions only those where the goal is to prevent falls by managing personal falls risk factors (2%).

Walking distance

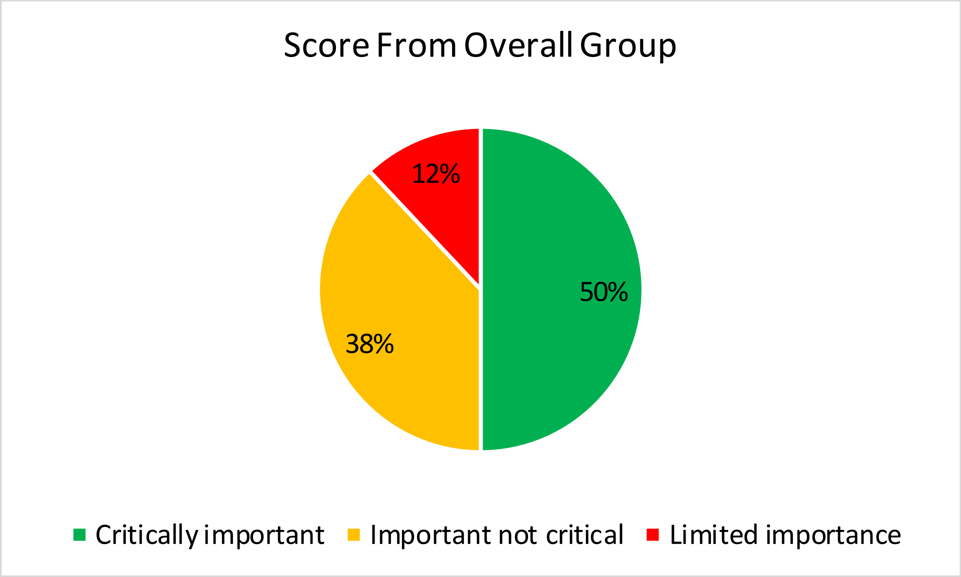

Scores From Each Stakeholder Group

Patients
Critically important: 83%
Important not critical: 17%
Limited importance: 0%

Researchers
Critically important: 30%
Important not critical: 35%
Limited importance: 35%

Clinicians
Critically important: 25%
Important not critical: 75%
Limited importance: 0%

Service-planners/Policymakers
Critically important: 86%
Important not critical: 14%
Limited importance: 0%

Reasons For Scores

Please note that the number in brackets shows the percentage of participants that gave that reason.

Reasons for including:
Important that patient can walk to a safe resting place (2%).

Reasons for excluding:
Distance covered should equate to distance needed to be covered at home to complete activities of daily living (2%).
Not related to the primary goal of the intervention (2%).

Walking self-efficacy

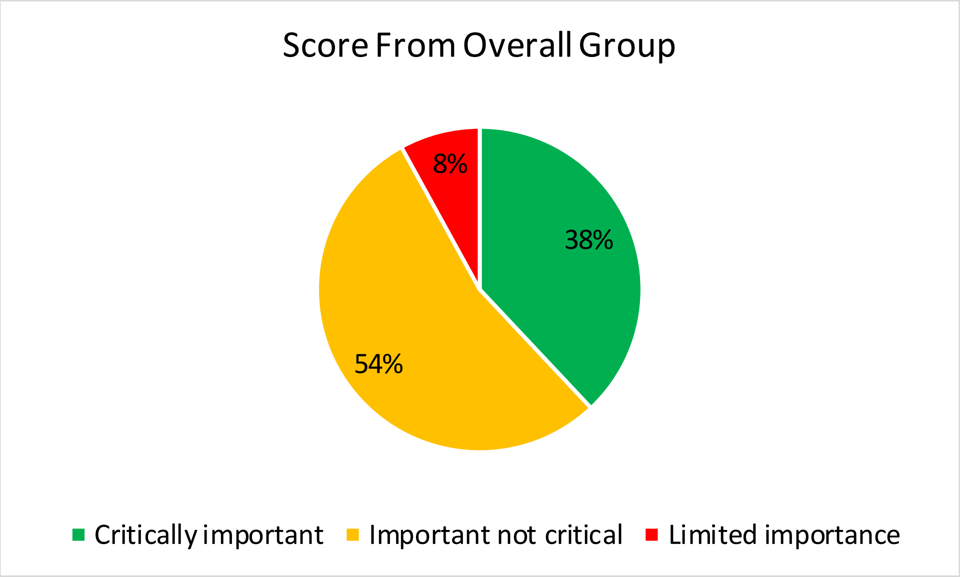

Scores From Each Stakeholder Group

Patients
Critically important: 42%
Important not critical: 58%
Limited importance: 0%

Researchers
Critically important: 29%
Important not critical: 47%
Limited importance: 24%

Clinicians
Critically important: 25%
Important not critical: 75%
Limited importance: 0%

Service-planners/Policymakers
Critically important: 71%
Important not critical: 29%
Limited importance: 0%

Reasons For Scores

Please note that the number in brackets shows the percentage of participants that gave that reason.

Reasons for including:
Enables positive risk taking (2%).

Reasons for excluding:
Not related to the primary goal of the intervention (2%).

Walking speed

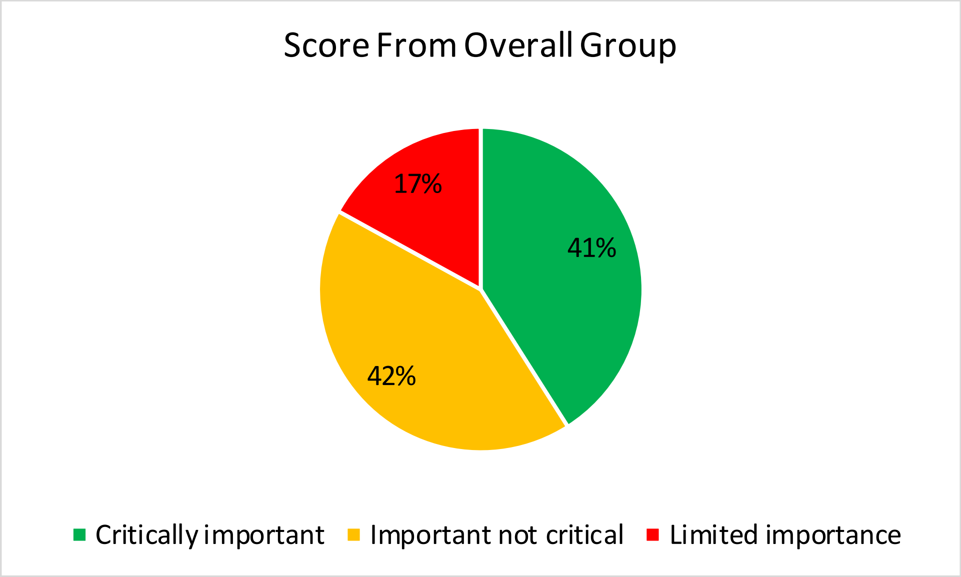

Scores From Each Stakeholder Group

Patients
Critically important: 42%
Important not critical: 50%
Limited importance: 8%

Researchers
Critically important: 30%
Important not critical: 35%
Limited importance: 35%

Clinicians
Critically important: 42%
Important not critical: 50%
Limited importance: 8%

Service-planners/Policymakers
Critically important: 71%
Important not critical: 29%
Limited importance: 0%

Reasons For Scores

Please note that the number in brackets shows the percentage of participants that gave that reason.

Reasons for including:
Assesses risk of falls (2%).
May influence disease progression (2%).

Reasons for excluding:
Reduced walking speed may be a positive outcome for some patients (4%).
Walking speed may not be an indication of falls risk (2%).
Not related to the primary goal of the intervention (2%).
