# Supplementary material for: A core outcome set for evaluating the effectiveness of mixed-diagnosis falls prevention interventions for people with Multiple Sclerosis, Parkinson’s Disease and stroke
Source: PLoS One. 2023 Nov 13;18(11):e0294193. doi: 10.1371/journal.pone.0294193 (PMC10642845; doi:10.1371/journal.pone.0294193)
Supplement: S4 Appendix — (PDF) [file pone.0294193.s004.pdf]

Appendix 4: Summary of survey responses from round two.

Activity curtailment due to fear of falling

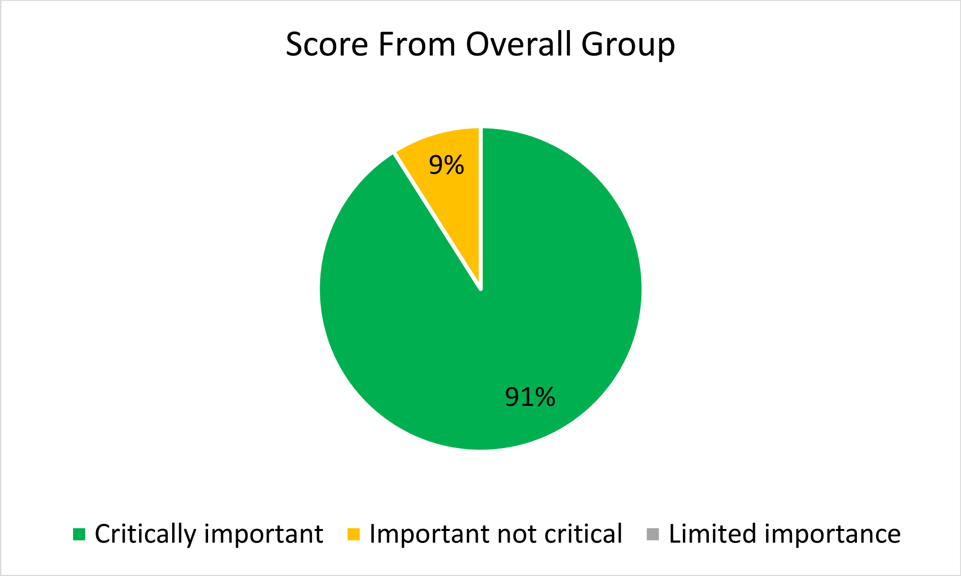

Scores From Each Stakeholder Group

Patients
Critically important: 87%
Important not critical: 13%
Limited importance: 0%

Researchers
Critically important: 82%
Important not critical: 18%
Limited importance: 0%

Clinicians
Critically important: 100%
Important not critical: 0%
Limited importance: 0%

Service-planners/Policymakers
Critically important: 100%
Important not critical: 0%
Limited importance: 0%

Reasons For Scores

Please note that the number in brackets shows the percentage of participants that gave that reason.

Reasons for including:
Influences quality of life (4%).
May enable positive risk taking 2%).
Reducing falls risk by limiting participation in activities does not indicate if an intervention is working (2%).
Necessary to contextualise of an intervention allows an individual to undertake more activities (2%).

Reasons for excluding:
Only relevant to those curtailing activities (2%).
In some participants increasing activity may increase falls risk (2%).

Anxiety

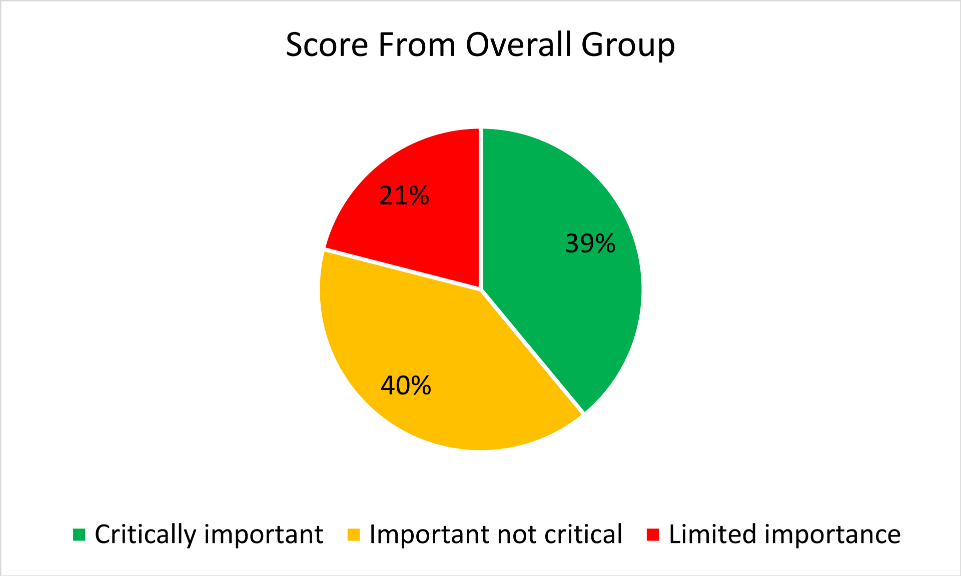

Scores From Each Stakeholder Group

Patients
Critically important: 62%
Important not critical: 38%
Limited importance:0%

Researchers
Critically important: 12%
Important not critical: 53%
Limited importance: 35%

Clinicians
Critically important: 36%
Important not critical: 46%
Limited importance: 18%

Service-planners/Policymakers
Critically important: 86%
Important not critical: 0%
Limited importance: 14%

Reasons For Scores

Please note that the number in brackets shows the percentage of participants that gave that reason.

<u>Reasons for including:</u>
Anxiety may impact balance (2%).
Contributes to fear of falling (2%).

<u>Reasons for excluding:</u>
Not relevant to all interventions only those where the goal is to prevent falls by improving anxiety (2%).
Treating anxiety would require a different intervention – unlikely to improve with a falls prevention intervention (2%).

Balance confidence

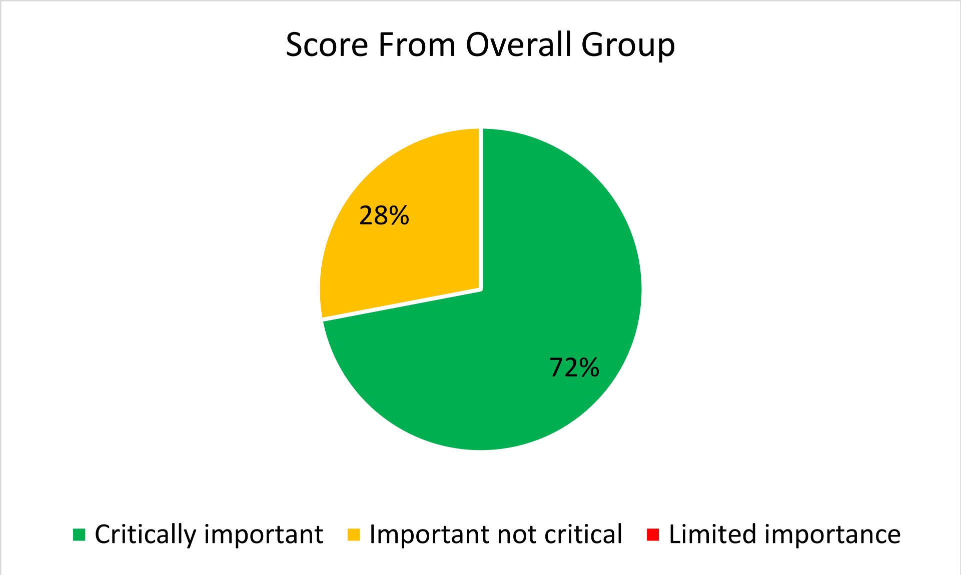

Scores From Each Stakeholder Group

Patients
Critically important: 87%
Important not critical: 13%
Limited importance: 0%

Researchers
Critically important: 59%
Important not critical: 41%
Limited importance: 0%

Clinicians
Critically important: 73%
Important not critical: 27%
Limited importance: 0%

Service-planners/Policymakers
Critically important: 86%
Important not critical: 14%
Limited importance: 0%

Reasons For Scores

Please note that the number in brackets shows the percentage of participants that gave that reason.

Reasons for including:
May reduce fear of falling (4%).

Reasons for excluding:
Overlaps with fear of falling and/or falls self-efficacy (2%).

Bone density

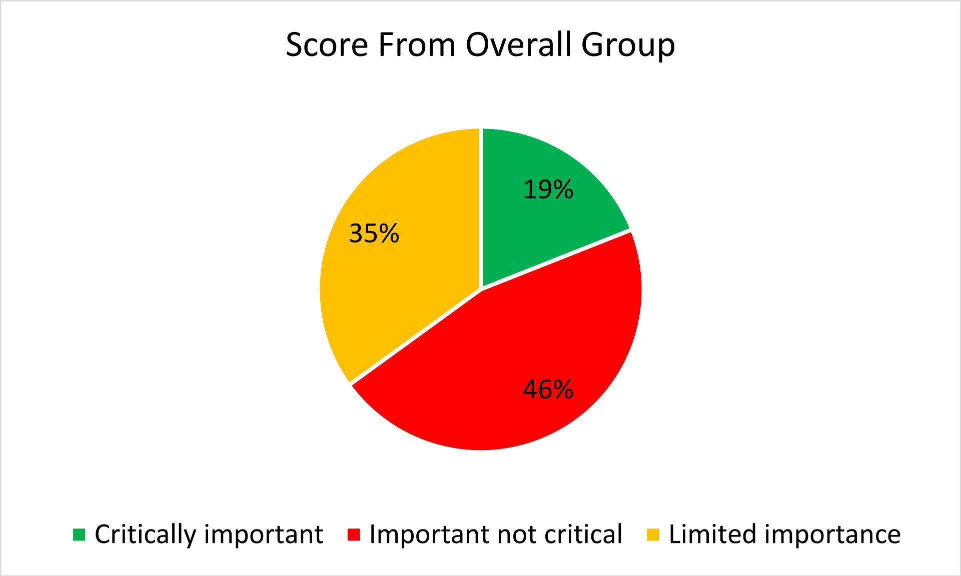

Scores From Each Stakeholder Group

Patients
Critically important: 50%
Important not critical: 13%
Limited importance: 37%

Researchers
Critically important: 0%
Important not critical: 41%
Limited importance: 59%

Clinicians
Critically important: 9%
Important not critical: 73%
Limited importance: 18%

Service-planners/Policymakers
Critically important: 43%
Important not critical: 57%
Limited importance: 0%

Reasons For Scores

Please note that the number in brackets shows the percentage of participants that gave that reason.

Reasons for including:
Falls in people with decreased bone density have worse outcomes (2%).

Reasons for excluding:
Not relevant to all participants (4%).
Potential for change is limited (4%).
Bone density is not related to the frequency of falls (4%).
Large sample size is required to detect change (2%).
Difficult to measure (2%).
Not related to the primary goal of the intervention (2%).

Bradykinesia

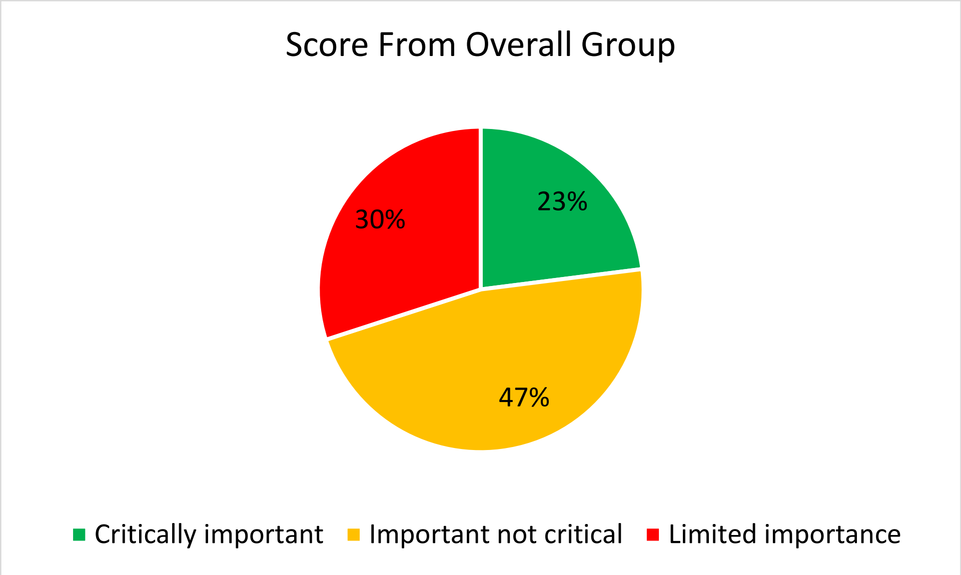

Scores From Each Stakeholder Group

Patients
Critically important: 37%
Important not critical: 38%
Limited importance: 25%

Researchers
Critically important: 6%
Important not critical: 29%
Limited importance: 65%

Clinicians
Critically important: 18%
Important not critical: 82%
Limited importance: 0%

Service-planners/Policymakers
Critically important: 57%
Important not critical: 43%
Limited importance: 0%

Reasons For Scores

Please note that the number in brackets shows the percentage of participants that gave that reason.

Reasons for including:
Slow reactions/responses to obstacles could lead to increased falls (2%).

Reasons for excluding:
More likely to be influenced by pharmacological management (4%).
Not relevant to all interventions only those aiming to prevent falls by improving bradykinesia (2%).
Moving slower could be safer for some individuals (2%).

Cadence

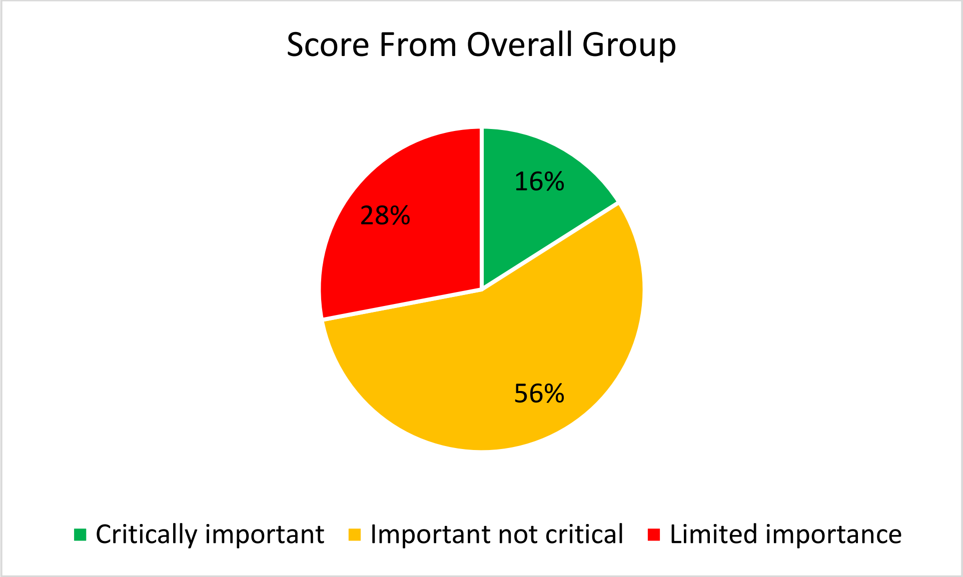

Scores From Each Stakeholder Group

Patients
Critically important: 12%
Important not critical: 75%
Limited importance: 13%

Researchers
Critically important: 6%
Important not critical: 41%
Limited importance: 53%

Clinicians
Critically important: 18%
Important not critical: 64%
Limited importance: 18%

Service-planners/Policymakers
Critically important: 43%
Important not critical: 57%
Limited importance: 0%

Reasons For Scores

Please note that the number in brackets shows the percentage of participants that gave that reason.

Reasons for excluding:
In Parkinson's disease can increase risk of falls if festinating (2%).
Quality of movement rather than speed may be of more significance (2%).
Not related to the primary goal of the intervention (2%).

Impact on carer

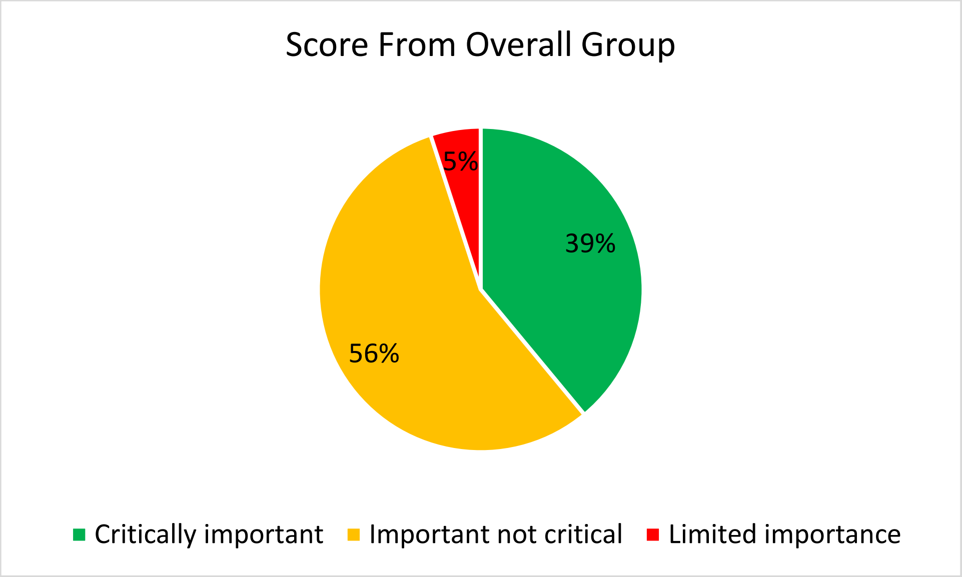

Scores From Each Stakeholder Group

Patients
Critically important: 50%
Important not critical: 37%
Limited importance: 13%

Researchers
Critically important: 23%
Important not critical: 71%
Limited importance: 6%

Clinicians
Critically important: 45%
Important not critical: 55%
Limited importance: 0%

Service-planners/Policymakers
Critically important: 57%
Important not critical: 43%
Limited importance: 0%

Reasons For Scores

Please note that the number in brackets shows the percentage of participants that gave that reason.

Reasons for excluding:
Not everyone has a carer so this should not be an outcome for all interventions (12%).

Cognition

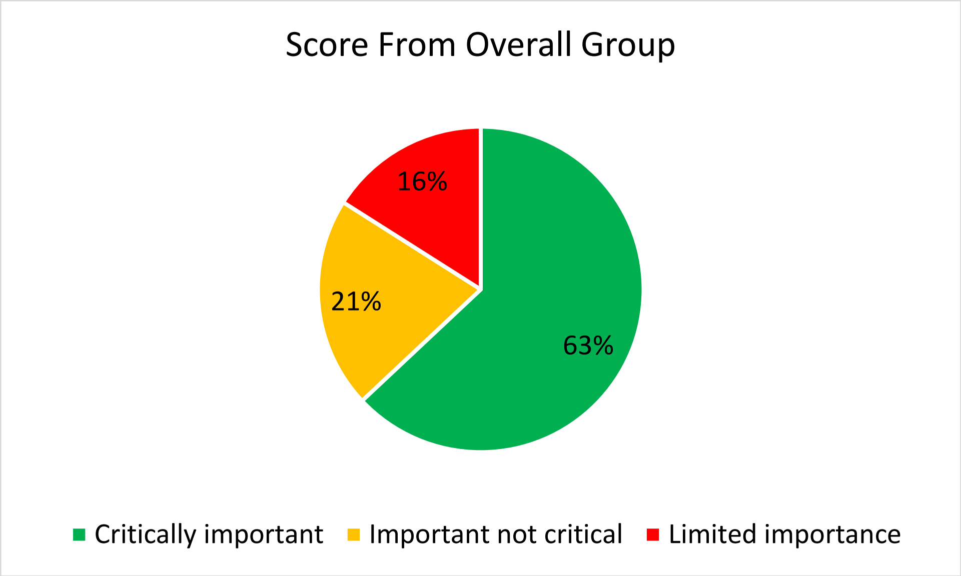

Scores From Each Stakeholder Group

Patients
Critically important: 87%
Important not critical: 13%
Limited importance: 0%

Researchers
Critically important: 35%
Important not critical: 35%
Limited importance: 30%

Clinicians
Critically important: 73%
Important not critical: 18%
Limited importance: 9%

Service-planners/Policymakers
Critically important: 86%
Important not critical: 0%
Limited importance: 14%

Reasons For Scores

Please note that the number in brackets shows the percentage of participants that gave that reason.

Reasons for including:
Cognitive impairment will affect insight into falls risk (4%).
Significant risk factor for falls (2%).
Intervention may improve safety awareness in those with a mild cognitive impairment (2%).

Reasons for excluding:
Unlikely to improve with a falls prevention intervention (14%).
Not relevant to all interventions only those aiming to prevent falls by improving cognition (2%).

Cost-effectiveness

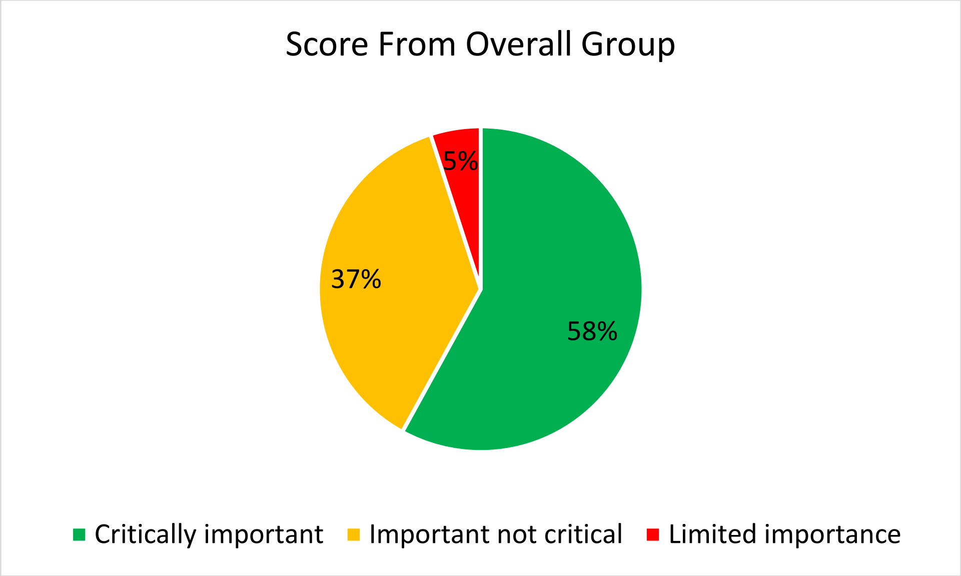

Scores From Each Stakeholder Group

Patients
Critically important: 62%
Important not critical: 25%
Limited importance: 13%

Researchers
Critically important: 76%
Important not critical: 24%
Limited importance: 0%

Clinicians
Critically important: 27%
Important not critical: 73%
Limited importance: 0%

Service-planners/Policymakers
Critically important: 57%
Important not critical: 29%
Limited importance: 14%

Reasons For Scores

Please note that the number in brackets shows the percentage of participants that gave that reason.

Reasons for including:
Important for adoption into healthcare settings (9%).
Important for access (2%).

Reasons for excluding:
Price should not condition the potential improvement in quality of life (2%).

Depression

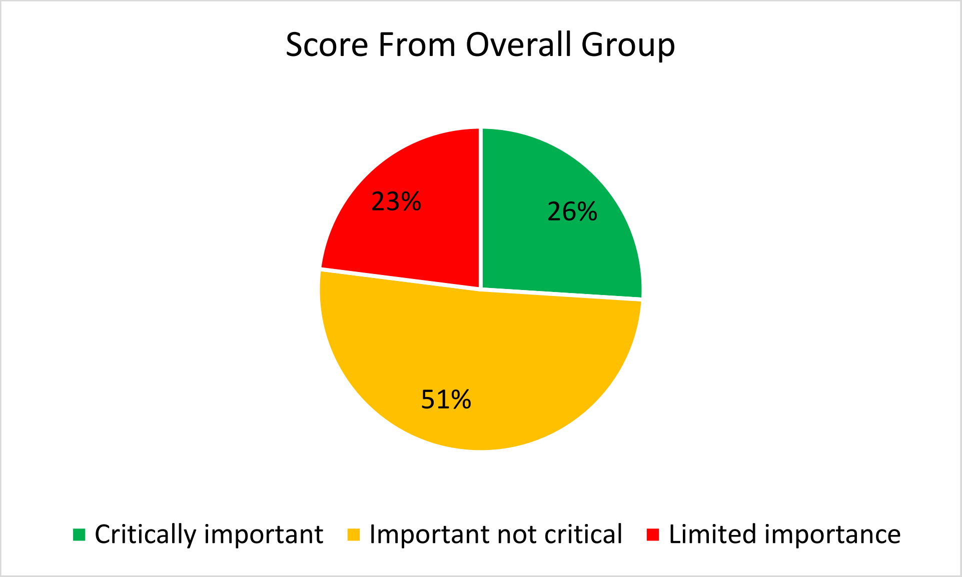

Scores From Each Stakeholder Group

Patients
Critically important: 37%
Important not critical: 50%
Limited importance: 13%

Researchers
Critically important: 18%
Important not critical: 47%
Limited importance: 35%

Clinicians
Critically important: 9%
Important not critical: 73%
Limited importance: 18%

Service-planners/Policymakers
Critically important: 57%
Important not critical: 29%
Limited importance: 14%

Reasons For Scores

Please note that the number in brackets shows the percentage of participants that gave that reason.

Reasons for including:
Falls risk factor for community-dwelling stroke survivors (2%).
Can impact overall physical health (2%).

Reasons for excluding:
Unlikely to improve with a falls prevention intervention (2%).

Disease impact

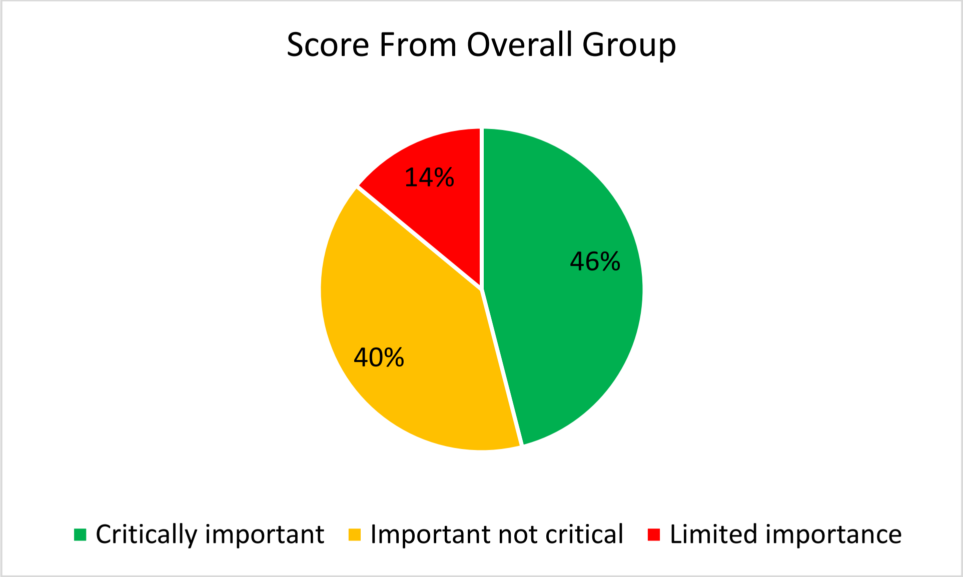

Scores From Each Stakeholder Group

Patients
Critically important: 87%
Important not critical: 0%
Limited importance: 13%

Researchers
Critically important: 29%
Important not critical: 42%
Limited importance: 29%

Clinicians
Critically important: 27%
Important not critical: 73%
Limited importance: 0%

Service-planners/Policymakers
Critically important: 71%
Important not critical: 29%
Limited importance: 0%

Reasons For Scores

Please note that the number in brackets shows the percentage of participants that gave that reason.

<u>Reasons for including:</u>
Disease impact is often linked to methods of cost-effectiveness and so should be measured (2%).

<u>Reasons for excluding:</u>
Not related to the primary goal of the intervention (2%).

Disease severity

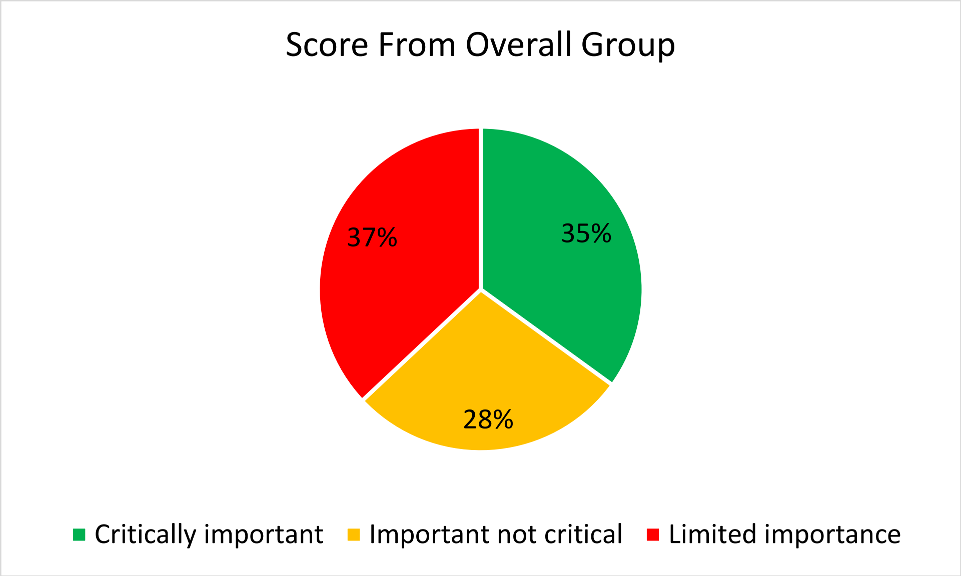

Scores From Each Stakeholder Group

Patients
Critically important: 75%
Important not critical: 25%
Limited importance: 0%

Researchers
Critically important: 12%
Important not critical: 18%
Limited importance: 70%

Clinicians
Critically important: 18%
Important not critical: 55%
Limited importance: 27%

Service-planners/Policymakers
Critically important: 57%
Important not critical: 29%
Limited importance: 14%

Reasons For Scores

Please note that the number in brackets shows the percentage of participants that gave that reason.

<u>Reasons for including:</u>
Advanced disease usually means a greater falls risk (4%).

<u>Reasons for excluding:</u>
Unlikely to improve with a falls prevention intervention (9%).
More of descriptor of participants than an outcome (7%).
Not related to the primary goal of the intervention (2%).
Difficult to measure accurately as many factors, such as social circumstances, can impact this outcome (2%).

Dizziness

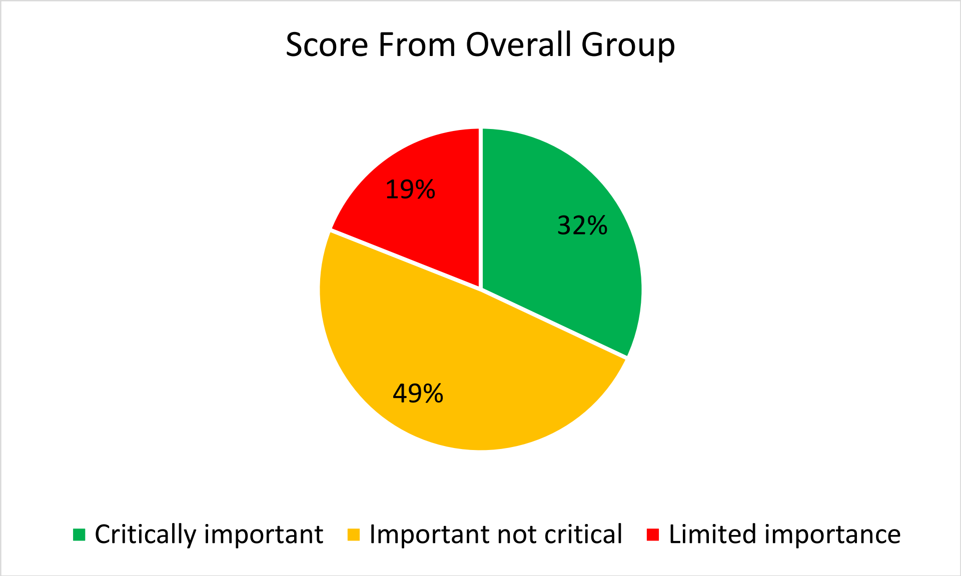

Scores From Each Stakeholder Group

Patients
Critically important: 62%
Important not critical: 25%
Limited importance: 13%

Researchers
Critically important: 12%
Important not critical: 53%
Limited importance: 35%

Clinicians
Critically important: 9%
Important not critical: 82%
Limited importance: 9%

Service-planners/Policymakers
Critically important: 71%
Important not critical: 29%
Limited importance: 0%

Reasons For Scores

Please note that the number in brackets shows the percentage of participants that gave that reason.

Reasons for including:
Increases risk of falls (4%).

Reasons for excluding:
Not relevant to all participants (7%).
Not relevant to all interventions only those aiming to prevent falls by improving dizziness (4%).
Unlikely to improve with a falls prevention intervention (4%).

Dual-tasking ability

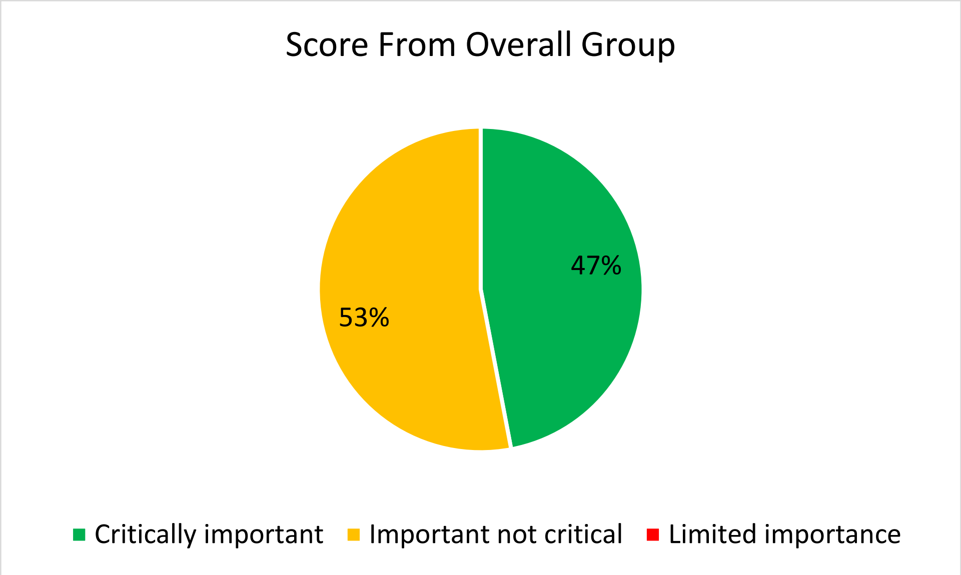

Scores From Each Stakeholder Group

Patients
Critically important: 87%
Important not critical: 13%
Limited importance: 0%

Researchers
Critically important: 76%
Important not critical: 24%
Limited importance: 0%

Clinicians
Critically important:45%
Important not critical: 55%
Limited importance: 0%

Service-planners/Policymakers
Critically important: 57%
Important not critical: 43%
Limited importance: 0%

Reasons For Scores

Please note that the number in brackets shows the percentage of participants that gave that reason.

Reasons for including:
Dual-task strategies are important and may reduce falls risk (4%).

Reasons for excluding:
Not relevant to all interventions only those aiming to prevent falls by improving dual-tasking ability (7%).

Dynamic balance

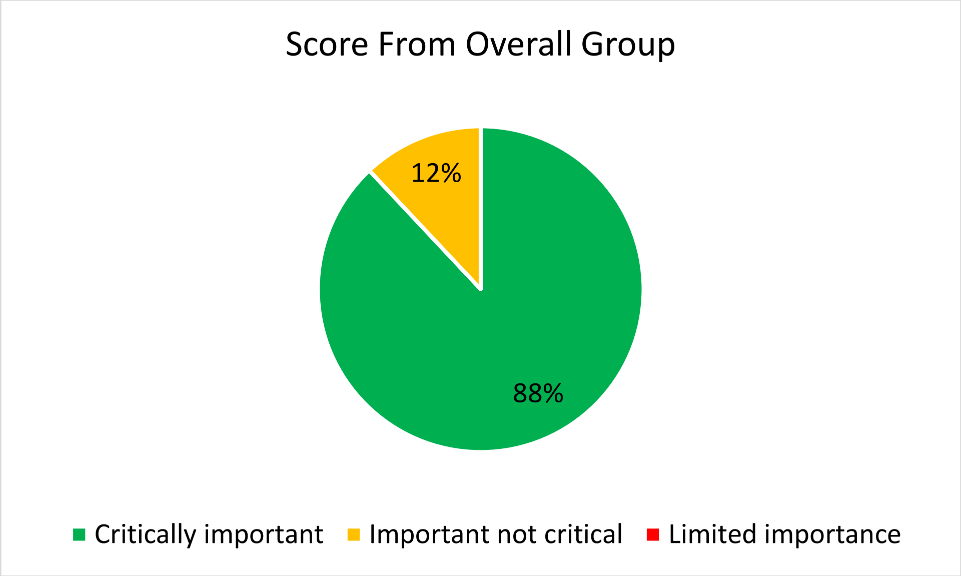

Scores From Each Stakeholder Group

Patients
Critically important: 100%
Important not critical: 0%
Limited importance: 0%

Researchers
Critically important: 82%
Important not critical: 18%
Limited importance: 0%

Clinicians
Critically important: 82%
Important not critical: 18%
Limited importance: 0%

Service-planners/Policymakers
Critically important: 100%
Important not critical: 0%
Limited importance: 0%

Reasons For Scores

Please note that the number in brackets shows the percentage of participants that gave that reason.

Reasons for including:
Most falls occur while an individual is moving (4%).
Biggest factor relating to fear avoidance (2%).

Reasons for excluding:
Not relevant to all interventions only those aiming to prevent falls by improving balance (2%).

Endurance

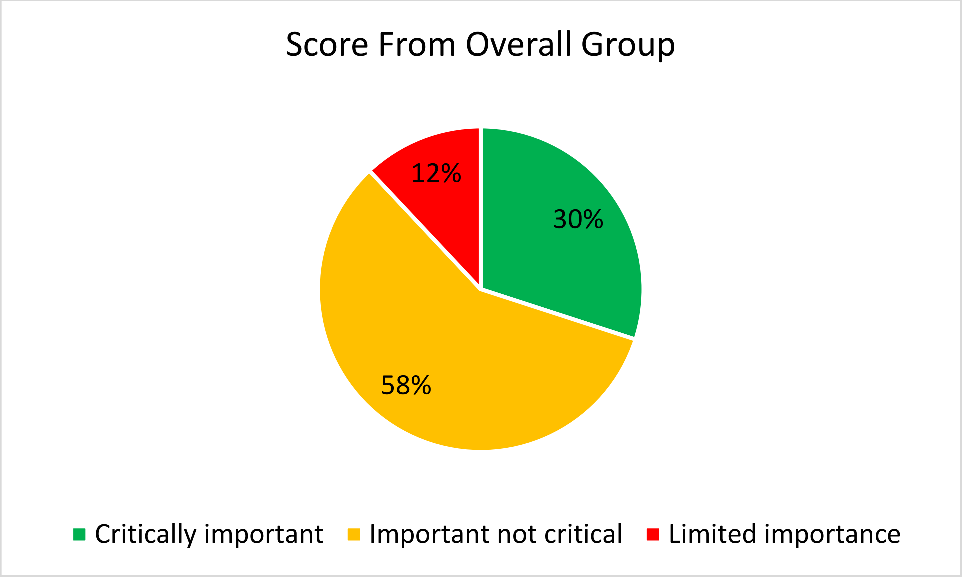

Scores From Each Stakeholder Group

Patients
Critically important: 37%
Important not critical: 50%
Limited importance: 13%

Researchers
Critically important: 6%
Important not critical: 76%
Limited importance: 18%

Clinicians
Critically important: 36%
Important not critical: 55%
Limited importance: 9%

Service-planners/Policymakers
Critically important: 71%
Important not critical: 29%
Limited importance: 0%

Reasons For Scores

Please note that the number in brackets shows the percentage of participants that gave that reason.

Reasons for including:
Allows individuals to participate in social activities (2%).

Reasons for excluding:
Not related to the primary goal of the intervention (4%).

Number of fall-related fractures

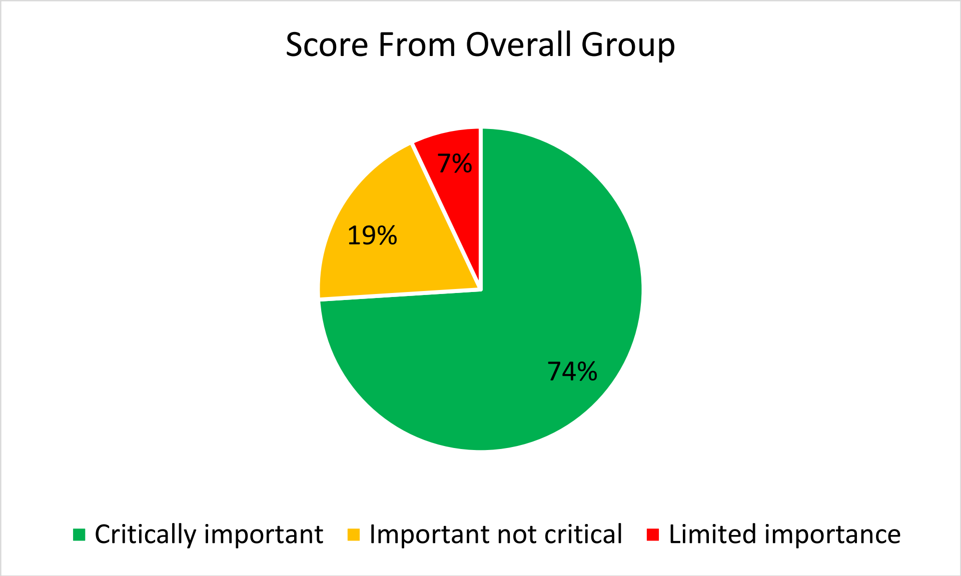

Scores From Each Stakeholder Group

Patients
Critically important: 62%
Important not critical: 13%
Limited importance: 25%

Researchers
Critically important: 76%
Important not critical: 18%
Limited importance: 6%

Clinicians
Critically important: 64%
Important not critical: 36%
Limited importance: 0%

Service-planners/Policymakers
Critically important: 100%
Important not critical: 0%
Limited importance: 0%

Reasons For Scores

Please note that the number in brackets shows the percentage of participants that gave that reason.

Reasons for including:
May assist in funding for falls prevention interventions (2%).
Fractures are often life-changing injuries with high personal and economic costs (2%).

Reasons for excluding:
Rare events and so most studies will be underpowered to show differences (4%).
Possibly captured in healthcare utilisation and so both outcomes may not be needed (2%).
Fractures should not be isolated from other long-term consequences of falls (2%).

Number of fallers

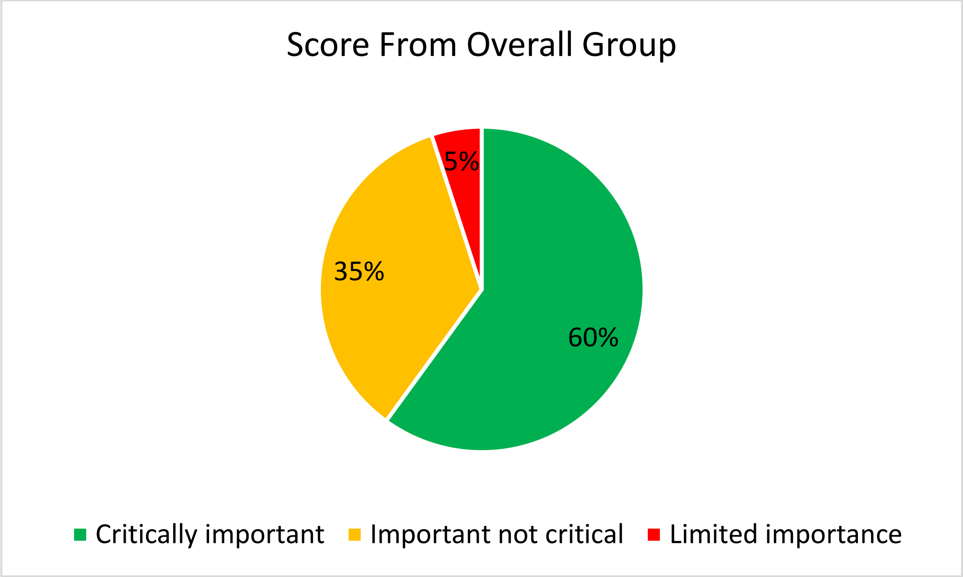

Scores From Each Stakeholder Group

Patients
Critically important: 50%
Important not critical: 37%
Limited importance: 13%

Researchers
Critically important: 76%
Important not critical: 18%
Limited importance: 6%

Clinicians
Critically important: 45%
Important not critical: 55%
Limited importance: 0%

Service-planners/Policymakers
Critically important: 57%
Important not critical: 43%
Limited importance: 0%

Reasons For Scores

Please note that the number in brackets shows the percentage of participants that gave that reason.

Reasons for including:
Direct and critical information to persuade funders when implementing programmes into real world healthcare settings (2%).
Can help to identify if one person who falls frequently is contributing to high numbers of falls (2%).

Reasons for excluding:
Single fall not necessarily meaningful (2%).
Individuals may not stop falling completely (2%).
May not be useful with heterogeneous groups (2%).

Falls rate

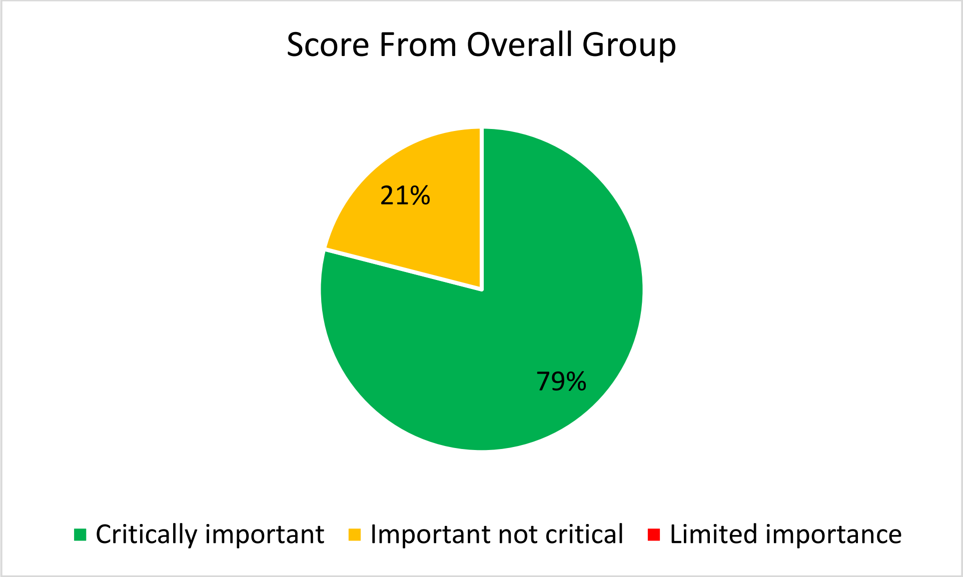

Scores From Each Stakeholder Group

Patients
Critically important: 62%
Important not critical: 38%
Limited importance: 0%

Researchers
Critically important: 100%
Important not critical: 0%
Limited importance: 0%

Clinicians
Critically important: 55%
Important not critical: 45%
Limited importance: 0%

Service-planners/Policymakers
Critically important: 86%
Important not critical: 14%
Limited importance: 0%

Reasons For Scores

Please note that the number in brackets shows the percentage of participants that gave that reason.

<u>Reasons for including:</u>
Critical information to persuade funders when implementing interventions into real-world healthcare settings (4%).
Primary goal of the intervention is to prevent falls (2%).

<u>Reasons for excluding:</u>
Difficult to record accurately (4%).

Number of falls resulting in healthcare utilisation

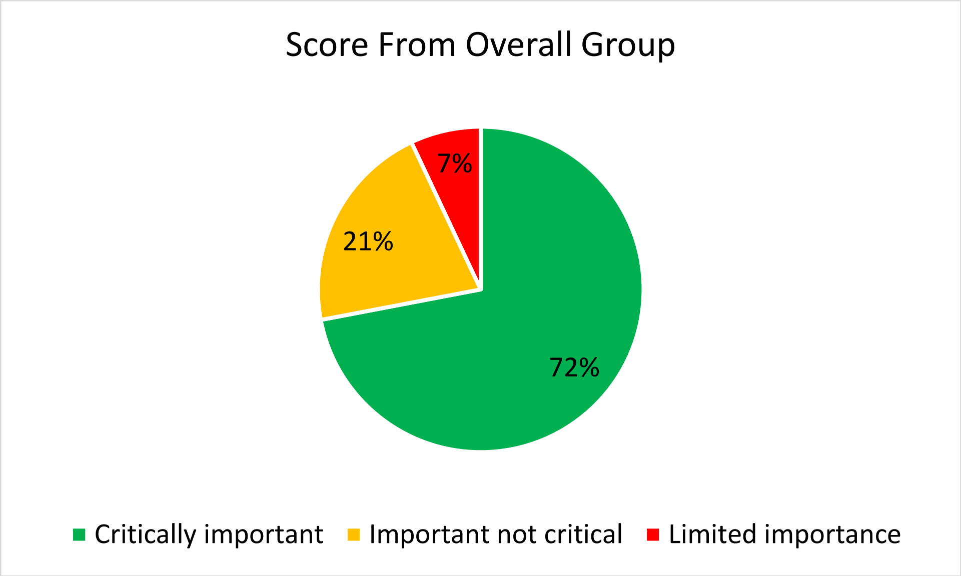

Scores From Each Stakeholder Group

Patients
Critically important: 62%
Important not critical: 13%
Limited importance:25%

Researchers
Critically important: 76%
Important not critical: 18%
Limited importance: 6%

Clinicians
Critically important: 64%
Important not critical: 36%
Limited importance: 0%

Service-planners/Policymakers
Critically important: 86%
Important not critical: 14%
Limited importance: 0%

Reasons For Scores

Please note that the number in brackets shows the percentage of participants that gave that reason.

Reasons for including:
Critical information to persuade funders when implementing interventions into real-world healthcare settings (4%).
Personal and economic cost of a hospital admission is high (2%).

Reasons for excluding:
Rare events and so most studies will be underpowered to show difference (2%).

Falls risk

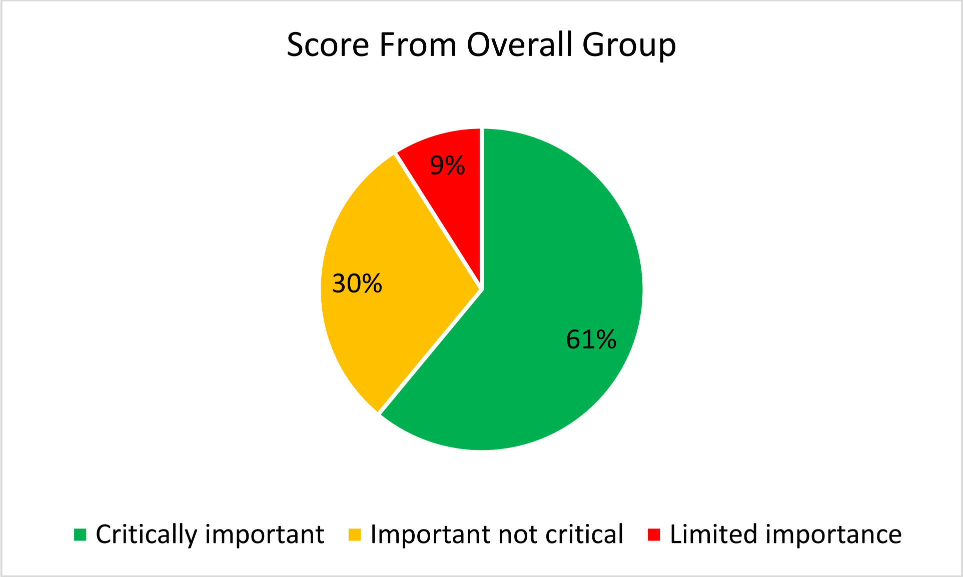

Scores From Each Stakeholder Group

Patients
Critically important: 62%
Important not critical: 25%
Limited importance: 13%

Researchers
Critically important: 41%
Important not critical: 47%
Limited importance: 12%

Clinicians
Critically important: 64%
Important not critical: 27%
Limited importance: 9%

Service-planners/Policymakers
Critically important: 100%
Important not critical: 0%
Limited importance: 0%

Reasons For Scores

Please note that the number in brackets shows the percentage of participants that gave that reason.

Reasons for including:
Reducing falls risk may minimise falls (2%).

Reasons for excluding:
Risk of falling is difficult to define/measure accurately (16%).
An intervention may not address the elements included in a particular definition of falls risk (2%).
Reducing risk but not the actual number of falls or injurious falls is not a successful intervention (2%).

Falls self-efficacy

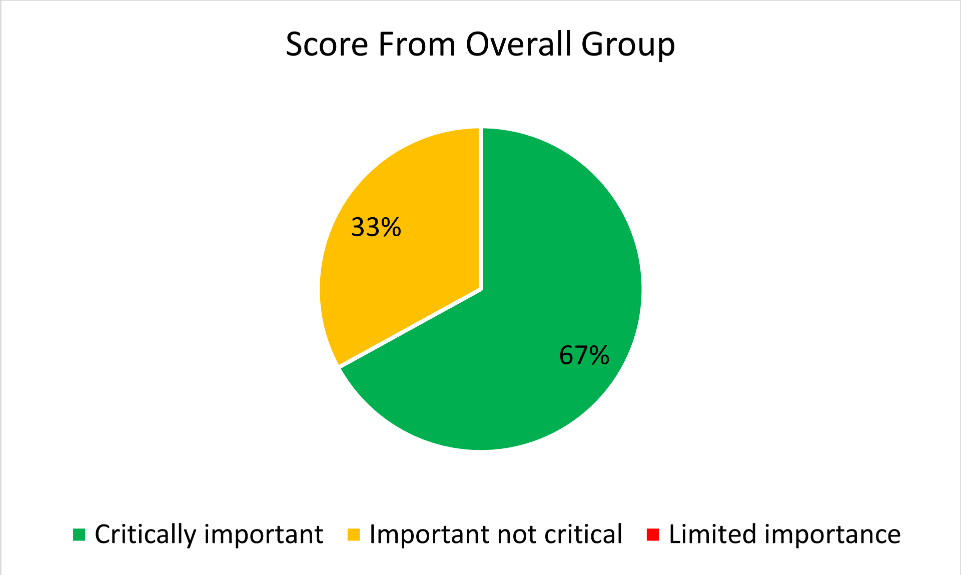

Scores From Each Stakeholder Group

Patients
Critically important: 62%
Important not critical: 38%
Limited importance: 0%

Researchers
Critically important: 76%
Important not critical: 24%
Limited importance: 0%

Clinicians
Critically important: 55%
Important not critical: 45%
Limited importance: 0%

Service-planners/Policymakers
Critically important: 71%
Important not critical: 29%
Limited importance: 0%

Reasons For Scores

Please note that the number in brackets shows the percentage of participants that gave that reason.

Reasons for including:
Could reduce fear of falling (2%).
May impact compliance with intervention (2%).
Confidence is an important component in preventing falls (2%).

Fatigue impact

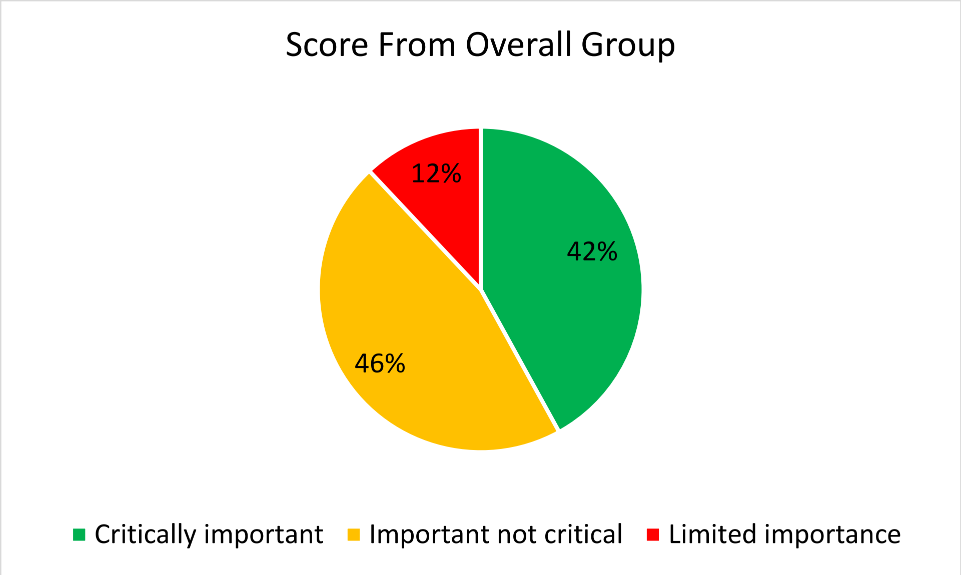

Scores From Each Stakeholder Group

Patients
Critically important: 87%
Important not critical: 0%
Limited importance: 13%

Researchers
Critically important: 76%
Important not critical: 0%
Limited importance: 24%

Clinicians
Critically important: 45%
Important not critical: 55%
Limited importance: 0%

Service-planners/Policymakers
Critically important: 86%
Important not critical: 14%
Limited importance: 0%

Reasons For Scores

Please note that the number in brackets shows the percentage of participants that gave that reason.

Reasons for including:
Fatigue may increase risk of falls (4%).

Reasons for excluding:
Not related to the primary goal of the intervention (9%).

Fatigue severity

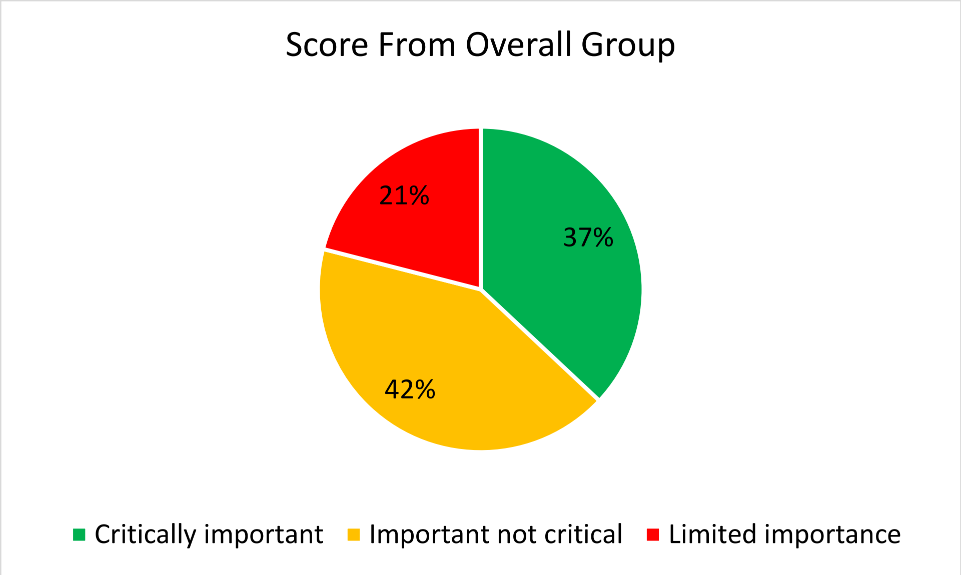

Scores From Each Stakeholder Group

Patients
Critically important: 75%
Important not critical: 0%
Limited importance: 25%

Researchers
Critically important: 6%
Important not critical: 59%
Limited importance: 35%

Clinicians
Critically important: 27%
Important not critical: 64%
Limited importance: 9%

Service-planners/Policymakers
Critically important: 86%
Important not critical: 14%
Limited importance: 0%

Reasons For Scores

Please note that the number in brackets shows the percentage of participants that gave that reason.

Reasons for including:
Fatigue may increase risk of falls (2%).

Reasons for excluding:
Not related to the primary goal of the intervention (9%).

Fitness

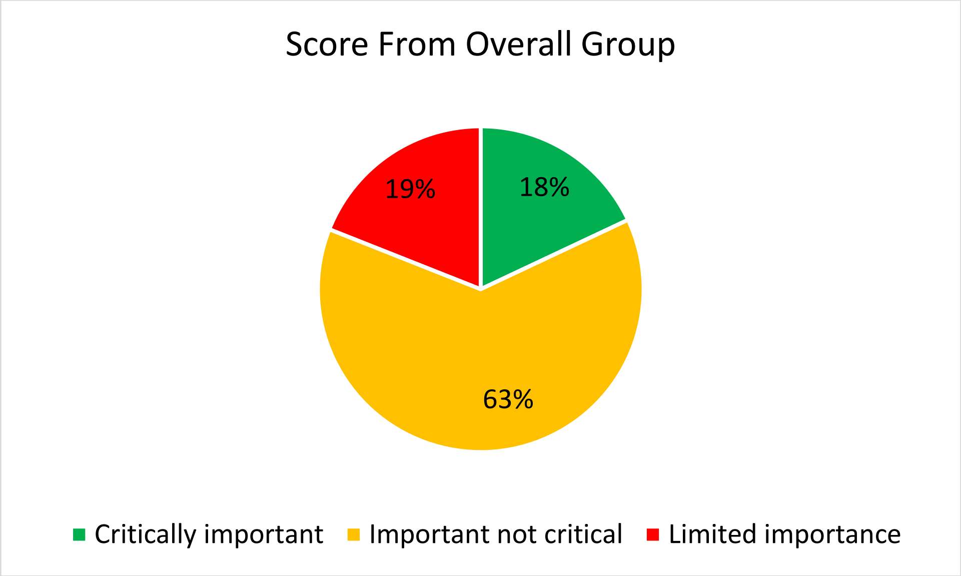

Scores From Each Stakeholder Group

Patients
Critically important: 50%
Important not critical: 50%
Limited importance: 0%

Researchers
Critically important: 6%
Important not critical: 59%
Limited importance: 35%

Clinicians
Critically important: 18%
Important not critical: 64%
Limited importance: 18%

Service-planners/Policymakers
Critically important: 14%
Important not critical: 86%
Limited importance: 0%

Reasons For Scores

Please note that the number in brackets shows the percentage of participants that gave that reason.

Reasons for including:
Important for physical activity and participation (2%).

Reasons for excluding:
Not related to the primary goal of the intervention (4%).

Flexibility

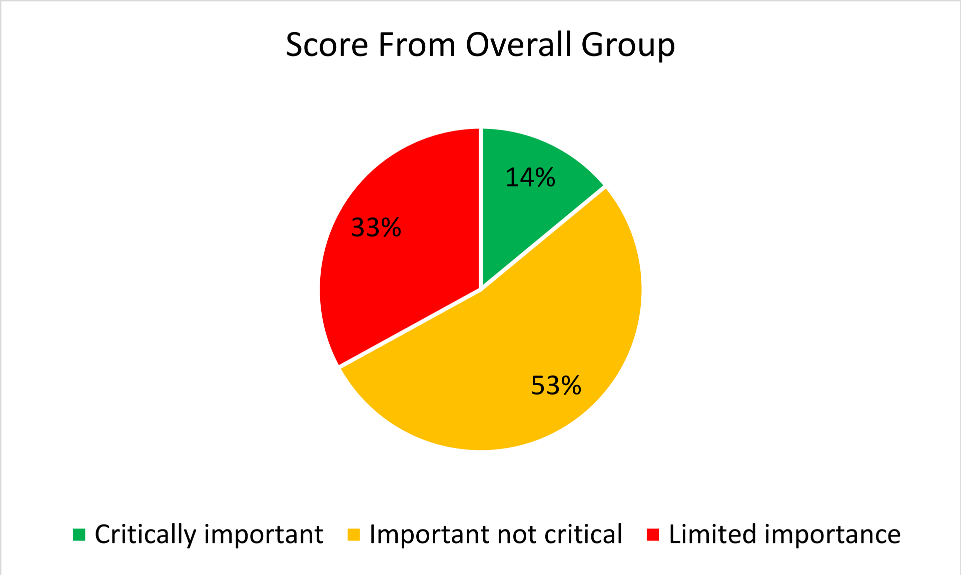

Scores From Each Stakeholder Group

Patients
Critically important: 50%
Important not critical: 50%
Limited importance: 0%

Researchers
Critically important: 0%
Important not critical: 41%
Limited importance: 59%

Clinicians
Critically important: 0%
Important not critical: 64%
Limited importance: 36%

Service-planners/Policymakers
Critically important: 29%
Important not critical: 71%
Limited importance: 0%

Reasons For Scores

Please note that the number in brackets shows the percentage of participants that gave that reason.

Reasons for including:
Important for maintaining balance (2%).

Reasons for excluding:
Not related to the primary goal of the intervention (4%).
Some participants may not improve flexibility due to increased tone (2%).

Fear of falling

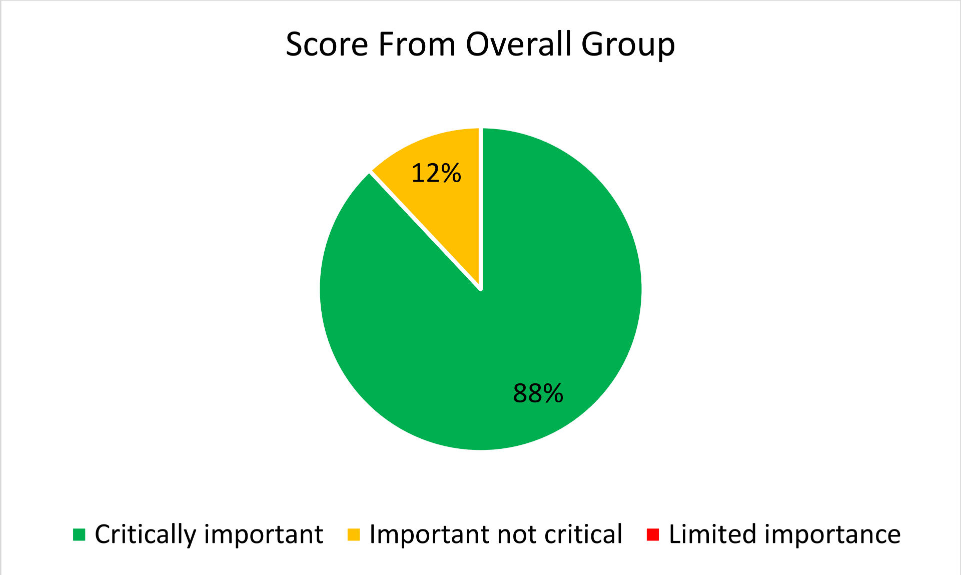

Scores From Each Stakeholder Group

Patients
Critically important: 87%
Important not critical: 13%
Limited importance: 0%

Researchers
Critically important: 76%
Important not critical: 24%
Limited importance: 0%

Clinicians
Critically important: 100%
Important not critical: 0%
Limited importance: 0%

Service-planners/Policymakers
Critically important: 100%
Important not critical: 0%
Limited importance: 0%

Reasons For Scores

Please note that the number in brackets shows the percentage of participants that gave that reason.

Reasons for including:
Very important to patients (2%).
Fear of falling increases risk of falls (2%).
Impacts gait, physical activity and participation (2%).

Freezing of gait

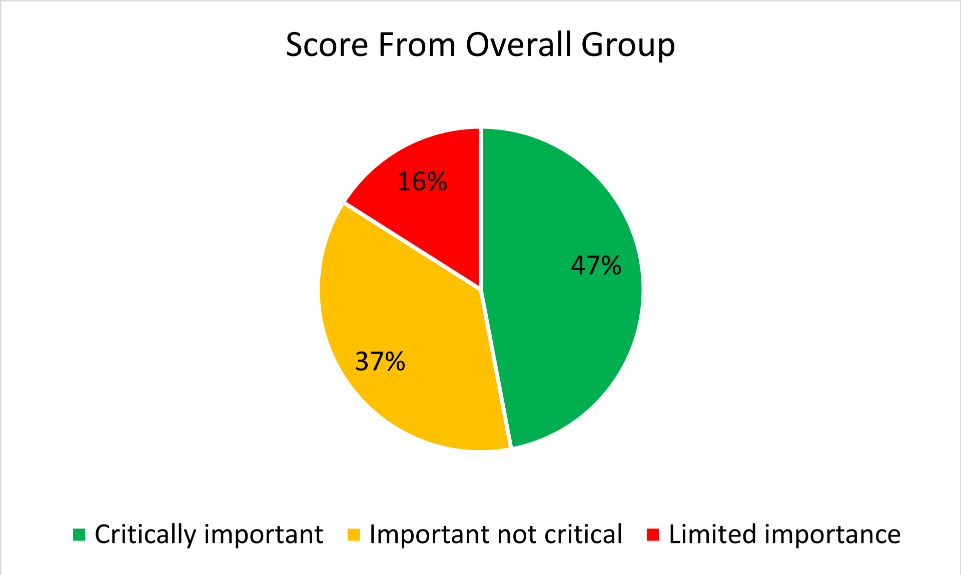

Scores From Each Stakeholder Group

Patients
Critically important: 75%
Important not critical: 12%
Limited importance: 13%

Researchers
Critically important: 24%
Important not critical: 41%
Limited importance: 35%

Clinicians
Critically important: 45%
Important not critical: 55%
Limited importance: 0%

Service-planners/Policymakers
Critically important: 71%
Important not critical: 29%
Limited importance: 0%

Reasons For Scores

Please note that the number in brackets shows the percentage of participants that gave that reason.

Reasons for including:
Strong risk factor for falls in Parkinson's disease (12%).

Reasons for excluding:
More likely to be influenced by pharmacological management (4%).
Unlikely to improve with a falls prevention intervention (2%).
Not relevant to all interventions only those aiming to prevent falls by improving freezing of gait (2%).

Knowledge of how to get up from the floor after a fall

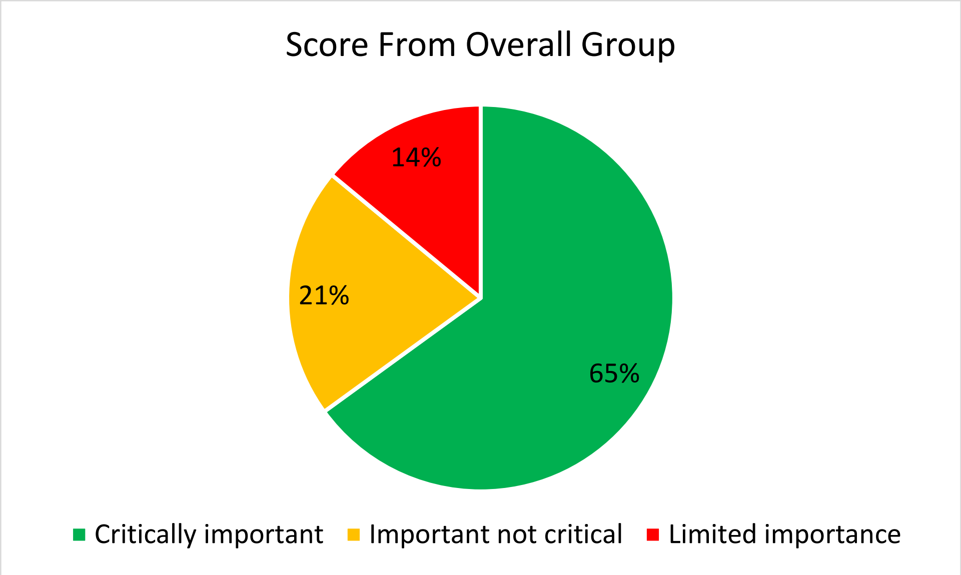

Scores From Each Stakeholder Group

Patients
Critically important: 75%
Important not critical: 13%
Limited importance: 12%

Researchers
Critically important: 41%
Important not critical: 30%
Limited importance: 29%

Clinicians
Critically important: 82%
Important not critical: 18%
Limited importance: 0%

Service-planners/Policymakers
Critically important: 100%
Important not critical: 0%
Limited importance: 0%

Reasons For Scores

Please note that the number in brackets shows the percentage of participants that gave that reason.

Reasons for including:
Will reduce the risk of serious outcomes associated with long lie (4%).
Reported as important by patients in qualitative research (2%).
Enables self-confidence and independence (2%).
Enables positive risk taking (2%).

Reasons for excluding:
Fall management rather than prevention (4%).
Ability rather than knowledge is more important (2%).
Key outcome is number of falls despite the ability to get up or not (2%).

Knowledge of how to fall

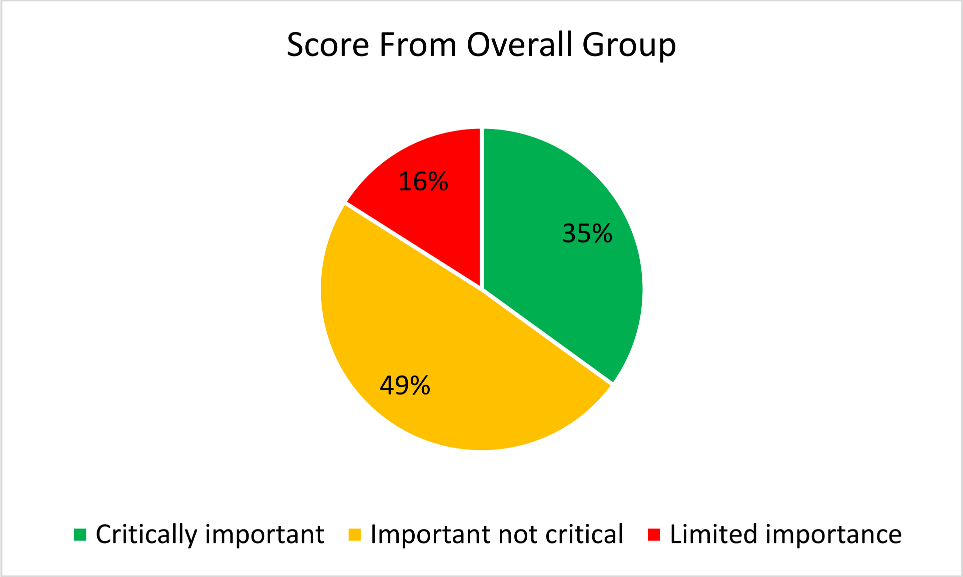

Scores From Each Stakeholder Group

Patients
Critically important: 62%
Important not critical: 25%
Limited importance: 13%

Researchers
Critically important: 24%
Important not critical: 41%
Limited importance: 35%

Clinicians
Critically important: 18%
Important not critical: 82%
Limited importance: 0%

Service-planners/Policymakers
Critically important: 57%
Important not critical: 43%
Limited importance: 0%

Reasons For Scores

Please note that the number in brackets shows the percentage of participants that gave that reason.

Reasons for including:
Unlikely to prevent all future falls but could prevent serious injury due to falls (4%).

Reasons for excluding:
Goal of intervention is to prevent falls, not to fall safely (4%).
Falls management rather than falls prevention (2%).
Most interventions do not include this component (2%).
Ability is more important than knowledge (2%).
Difficult to measure (2%).
Unsure how this would be taught (2%).

Ability to independently perform activities of daily living

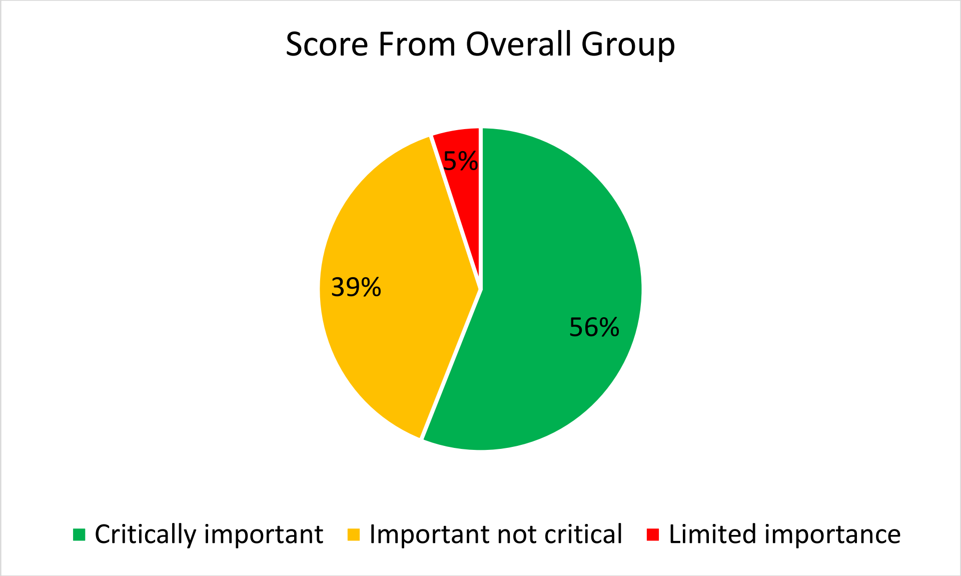

Scores From Each Stakeholder Group

Patients
Critically important: 75%
Important not critical: 25%
Limited importance: 0%

Researchers
Critically important: 41%
Important not critical: 53%
Limited importance: 6%

Clinicians
Critically important: 55%
Important not critical: 36%
Limited importance: 9%

Service-planners/Policymakers
Critically important: 71%
Important not critical: 29%
Limited importance: 0%

Reasons For Scores

Please note that the number in brackets shows the percentage of participants that gave that reason.

Reasons for including:
Often is a key goal for patients (9%).

Of note, some participants who scored this outcome as critically important highlighted that independence could only be facilitated to the point that it is reasonably safe (2%).

Reasons for excluding:
May not be physically possible for the patient to complete all activities independently (4%).
Completion of activities of daily living has many factors and the contents of a falls prevention intervention may not sufficiently address these and so this outcome may not change due to a falls prevention intervention (2%).
Asking for appropriate support may be an effective falls prevention strategy (2%).
Not related to the primary goal of the intervention (2%).

Number of injurious falls

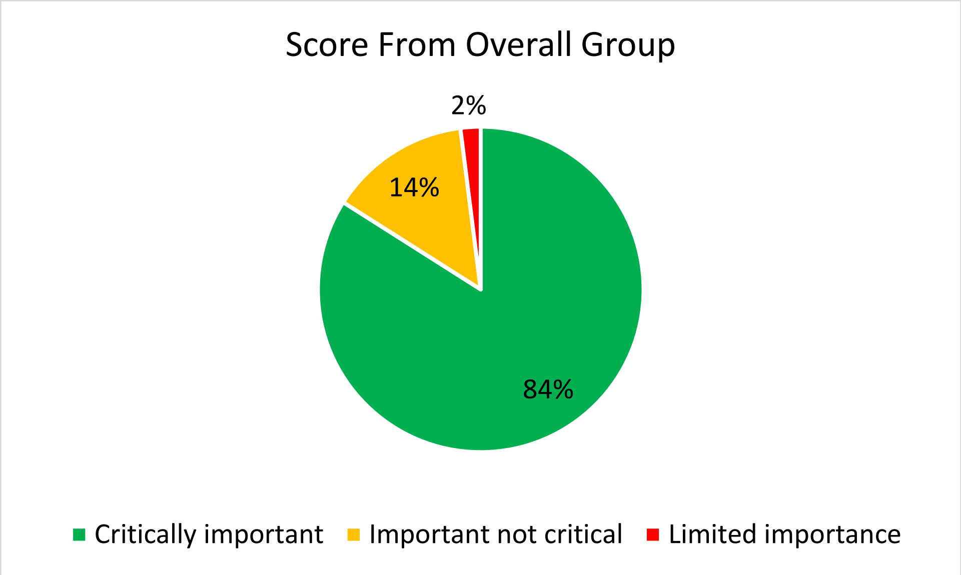

Scores From Each Stakeholder Group

Patients
Critically important: 75%
Important not critical: 25%
Limited importance: 0%

Researchers
Critically important: 88%
Important not critical: 12%
Limited importance: 0%

Clinicians
Critically important: 73%
Important not critical: 18%
Limited importance: 9%

Service-planners/Policymakers
Critically important: 100%
Important not critical: 0%
Limited importance: 0%

Reasons For Scores

Please note that the number in brackets shows the percentage of participants that gave that reason.

Reasons for including:
Critical information to persuade funders when implementing interventions into real-world healthcare settings (4%).
Injuries have a personal and economic cost (2%).

Reasons for excluding:
Rare events and so most studies will be underpowered to show difference (4%).
Difficult to define (2%).

Level of physical activity

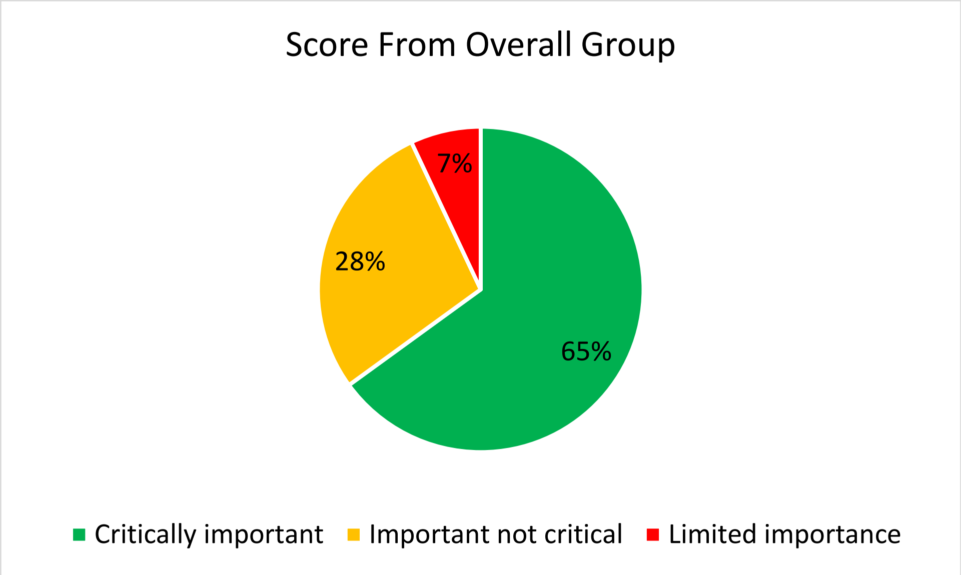

Scores From Each Stakeholder Group

Patients
Critically important: 75%
Important not critical: 25%
Limited importance: 0%

Researchers
Critically important: 47%
Important not critical: 35%
Limited importance: 18%

Clinicians
Critically important: 64%
Important not critical: 36%
Limited importance: 0%

Service-planners/Policymakers
Critically important: 100%
Important not critical: 0%
Limited importance: 0%

Reasons For Scores

Please note that the number in brackets shows the percentage of participants that gave that reason.

Reasons for including:
Can help to understand fall rates in relation to exposure – to demonstrate falls are not being reduced by people not moving around (4%).
Important for falls prevention (2%).
Captures increased participation/socialisation which may have been reduced due to fear of falling/activity curtailment (2%).
Increased physical activity may be an indication of improved balance (2%).

Lower limb strength

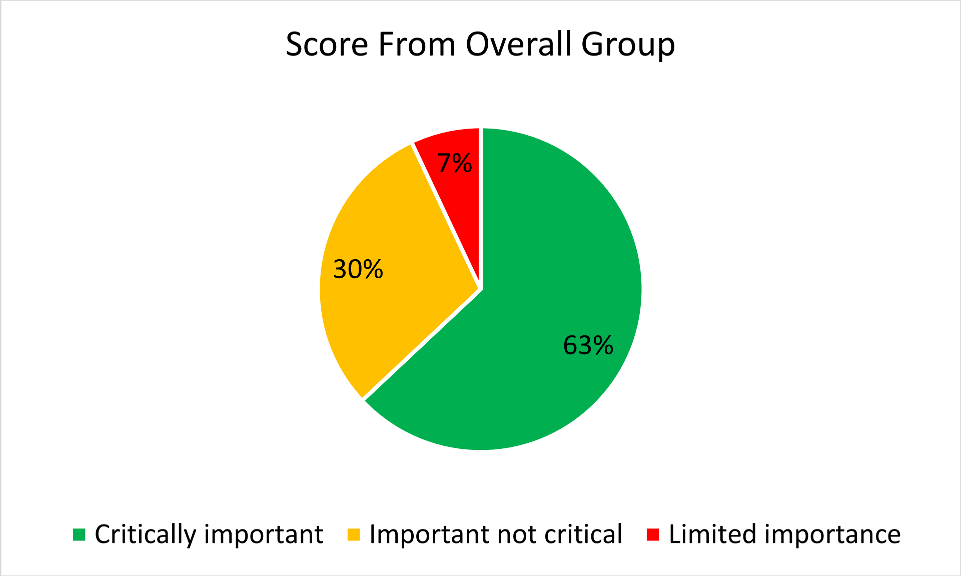

Scores From Each Stakeholder Group

Patients
Critically important: 62%
Important not critical: 38%
Limited importance: 0%

Researchers
Critically important: 47%
Important not critical: 35%
Limited importance: 18%

Clinicians
Critically important: 64%
Important not critical: 36%
Limited importance: 0%

Service-planners/Policymakers
Critically important: 100%
Important not critical: 0%
Limited importance: 0%

Reasons For Scores

Please note that the number in brackets shows the percentage of participants that gave that reason.

Reasons for including:
Important for falls prevention (7%).
Necessary to get up from floor (2%).

Reasons for excluding:
Not relevant to all interventions only those where the goal is to prevent falls by improving lower limb strength (2%).

Number of falls resulting in a long lie

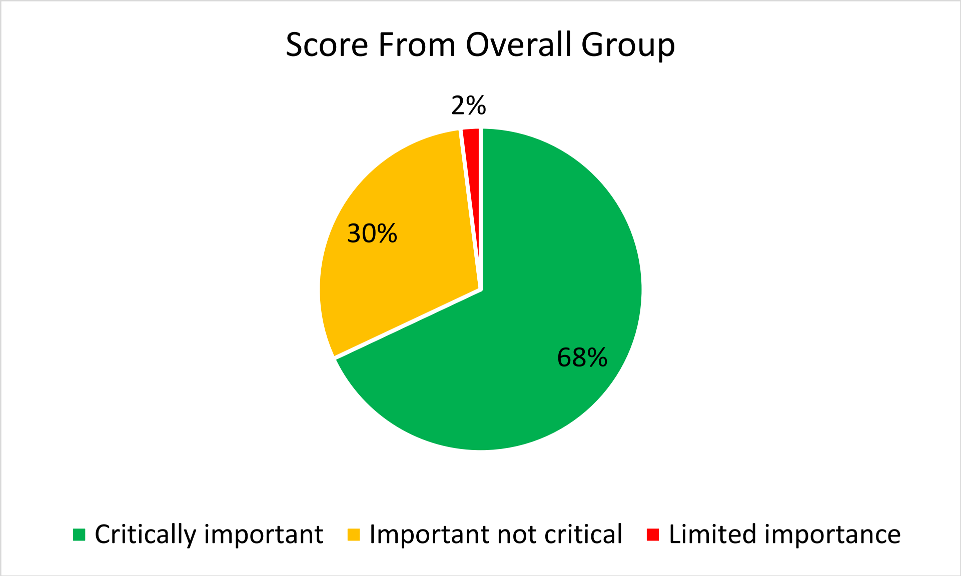

Scores From Each Stakeholder Group

Patients
Critically important: 62%
Important not critical: 25%
Limited importance: 13%

Researchers
Critically important: 53%
Important not critical: 47%
Limited importance: 0%

Clinicians
Critically important: 73%
Important not critical: 27%
Limited importance: 0%

Service-planners/Policymakers
Critically important: 100%
Important not critical: 0%
Limited importance: 0%

Reasons For Scores

Please note that the number in brackets shows the percentage of participants that gave that reason.

<u>Reasons for including:</u>
Can influence patient outcomes (4%).
Contributes to fear of falling (2%).
These events are important to patients (2%).

<u>Reasons for excluding:</u>
Not relevant to all interventions only those where the intervention would improve ability to get up after a fall (2%).

Number of near falls

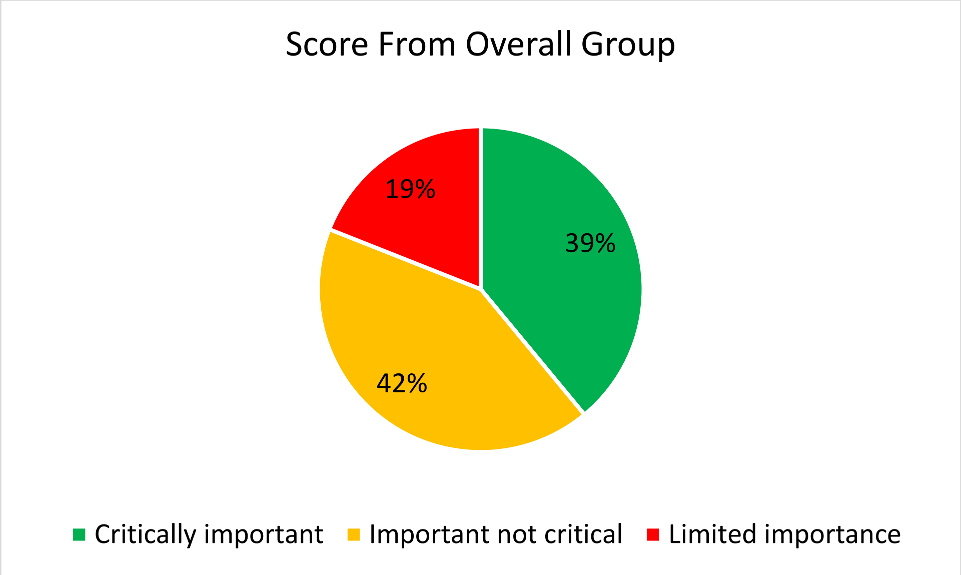

Scores From Each Stakeholder Group

Patients
Critically important: 62%
Important not critical: 38%
Limited importance: 0%

Researchers
Critically important: 24%
Important not critical: 35%
Limited importance: 41%

Clinicians
Critically important: 27%
Important not critical: 64%
Limited importance: 9%

Service-planners/Policymakers
Critically important: 71%
Important not critical: 29%
Limited importance: 0%

Reasons For Scores

Please note that the number in brackets shows the percentage of participants that gave that reason.

Reasons for excluding:
Difficult to define (9%).
Very unreliable/inaccurate measure (4%).
Unclear if near falls predict future falls (2%).

Total number of falls

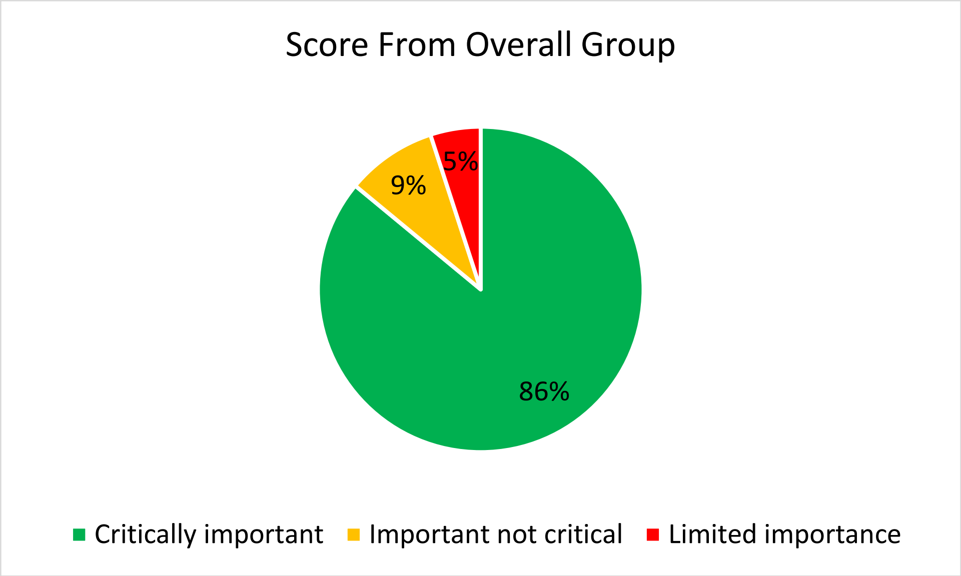

Scores From Each Stakeholder Group

Patients
Critically important: 87%
Important not critical: 13%
Limited importance: 0%

Researchers
Critically important: 82%
Important not critical: 6%
Limited importance: 12%

Clinicians
Critically important: 91%
Important not critical: 9%
Limited importance: 0%

Service-planners/Policymakers
Critically important: 86%
Important not critical: 14%
Limited importance: 0%

Reasons For Scores

Please note that the number in brackets shows the percentage of participants that gave that reason.

Reasons for including:
Primary goal of the intervention is to prevent falls (4%).
Critical information to persuade funders when implementing interventions into real-world healthcare settings (2%).

Reasons for excluding:
Better captured by falls rate (2%).
Does not account for the time period over which falls were recorded (2%).
Issues with validity of this outcome (2%).

Objectively assessed ability to perform activities of daily living

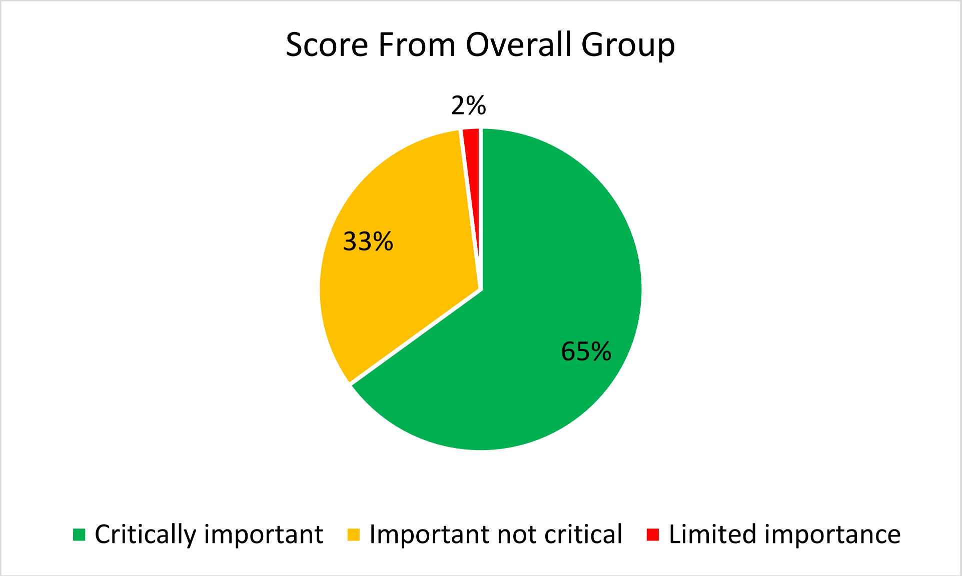

Scores From Each Stakeholder Group

Patients
Critically important: 87%
Important not critical: 13%
Limited importance: 0%

Researchers
Critically important: 35%
Important not critical: 59%
Limited importance: 6%

Clinicians
Critically important: 82%
Important not critical: 18%
Limited importance: 0%

Service-planners/Policymakers
Critically important: 86%
Important not critical: 14%
Limited importance: 0%

Reasons For Scores

Please note that the number in brackets shows the percentage of participants that gave that reason.

Reasons for including:
Objective assessment is more reliable (2%).
Objective assessment can highlight issues (2%).
Rated highly by patients (2%).

Reasons for excluding:
Not related to the primary goal of the intervention (2%).
Does not capture how patient feels (2%).
Completion of activities of daily living has many factors and the contents of a falls prevention intervention may not sufficiently address these and so this outcome may not change due to a falls prevention intervention (2%).
Safety awareness during task can only be assessed through objective assessment (2%).

Objectively assessed mobility

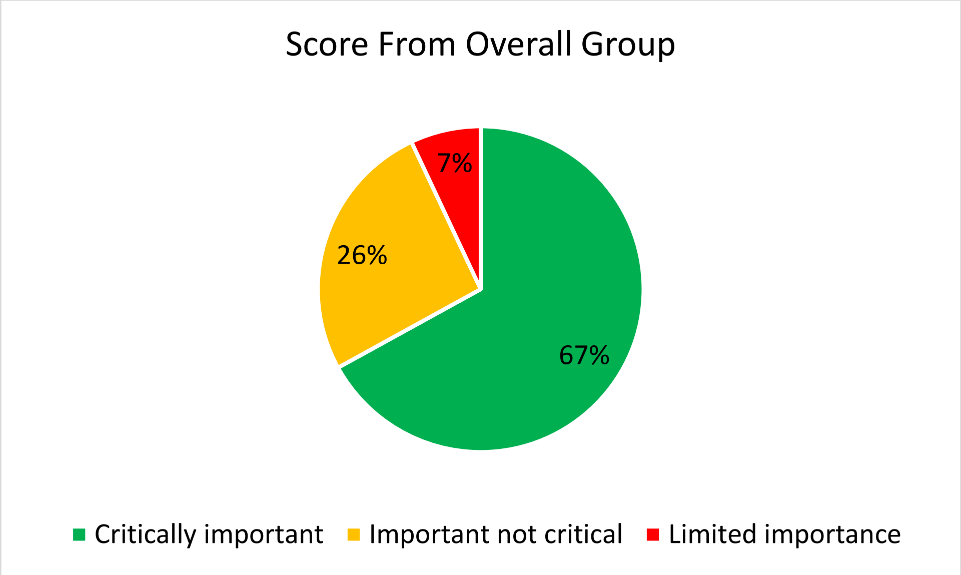

Scores From Each Stakeholder Group

Patients
Critically important: 100%
Important not critical: 0%
Limited importance: 0%

Researchers
Critically important: 41%
Important not critical: 41%
Limited importance: 18%

Clinicians
Critically important: 73%
Important not critical: 27%
Limited importance: 0%

Service-planners/Policymakers
Critically important: 86%
Important not critical: 14%
Limited importance: 0%

Reasons For Scores

Please note that the number in brackets shows the percentage of participants that gave that reason.

Reasons for including:
Efficient gait pattern minimises falls risk (4%).
Can provide insight into causes of falls (2%).

Reasons for excluding:
Not related to the primary goal of the intervention (4%).

Pain

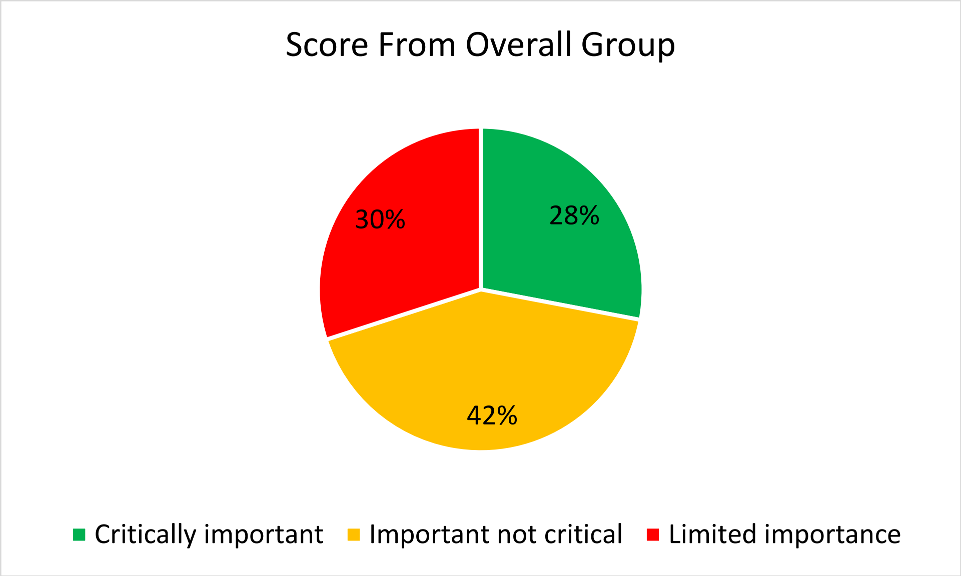

Scores From Each Stakeholder Group

Patients
Critically important: 62%
Important not critical: 38%
Limited importance: 0%

Researchers
Critically important: 0%
Important not critical: 35%
Limited importance: 65%

Clinicians
Critically important: 9%
Important not critical: 73%
Limited importance: 18%

Service-planners/Policymakers
Critically important: 86%
Important not critical: 14%
Limited importance: 0%

Reasons For Scores

Please note that the number in brackets shows the percentage of participants that gave that reason.

Reasons for excluding:
Not relevant to all participants (4%).
Not related to the primary goal of the intervention (4%).

Peer support

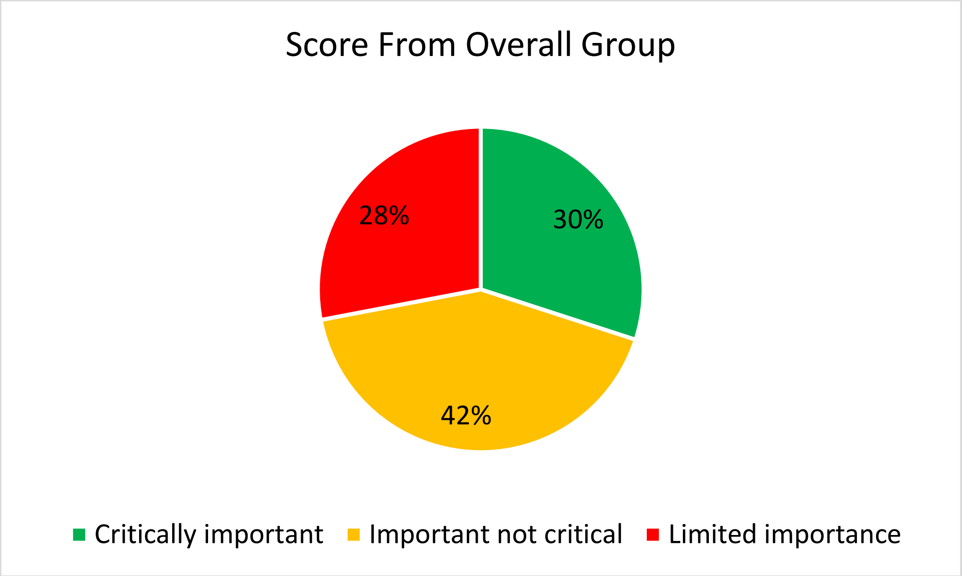

Scores From Each Stakeholder Group

Patients
Critically important: 62%
Important not critical: 38%
Limited importance: 0%

Researchers
Critically important: 6%
Important not critical: 41%
Limited importance: 53%

Clinicians
Critically important: 18%
Important not critical: 73%
Limited importance: 9%

Service-planners/Policymakers
Critically important: 71%
Important not critical: 0%
Limited importance: 29%

Reasons For Scores

Please note that the number in brackets shows the percentage of participants that gave that reason.

Reasons for including:
May provide mental health support which may impact on overall wellbeing (2%).

Reasons for excluding:
Reflects process rather than an outcome of the intervention (4%).
Not related to the primary goal of the intervention (4%).
Difficult to quantify (2%).

Perceived control of falls

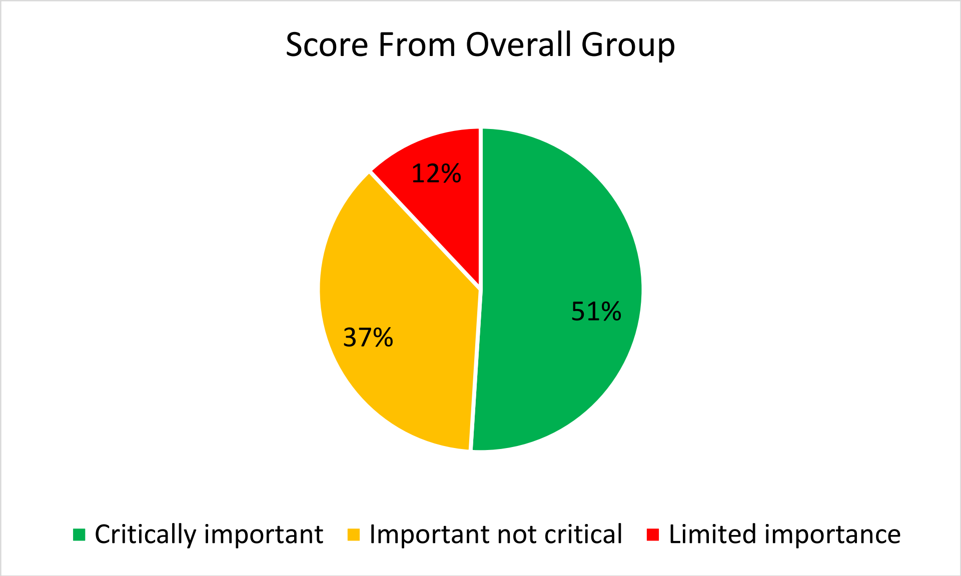

Scores From Each Stakeholder Group

Patients
Critically important: 50%
Important not critical: 37%
Limited importance: 13%

Researchers
Critically important: 53%
Important not critical: 23%
Limited importance: 24%

Clinicians
Critically important: 36%
Important not critical: 64%
Limited importance: 0%

Service-planners/Policymakers
Critically important: 71%
Important not critical: 29%
Limited importance: 0%

Reasons For Scores

Please note that the number in brackets shows the percentage of participants that gave that reason.

Reasons for including:
Enables self-confidence and increased participation (2%).

Reasons for excluding:
Difficult to measure (2%).
This is a vague concept (2%).

Self-perceived impact on carer/family

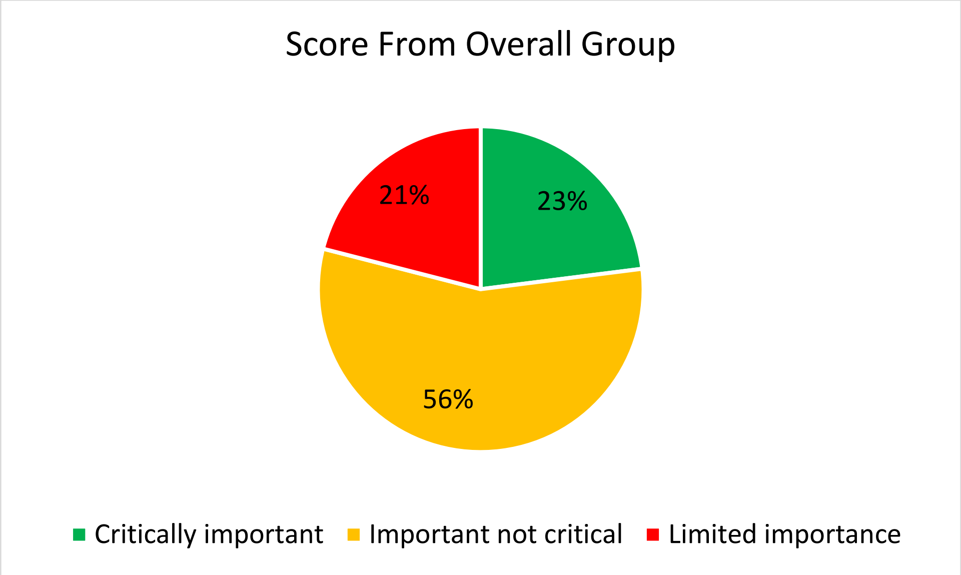

Scores From Each Stakeholder Group

Patients
Critically important: 50%
Important not critical: 50%
Limited importance: 0%

Researchers
Critically important: 6%
Important not critical: 53%
Limited importance: 41%

Clinicians
Critically important: 18%
Important not critical: 64%
Limited importance: 18%

Service-planners/Policymakers
Critically important: 43%
Important not critical: 57%
Limited importance: 0%

Reasons For Scores

Please note that the number in brackets shows the percentage of participants that gave that reason.

<u>Reasons for including:</u>
Could act as a motivational factor (2%).

<u>Reasons for excluding:</u>
Not related to the primary goal of the intervention (12%).

Quality of life

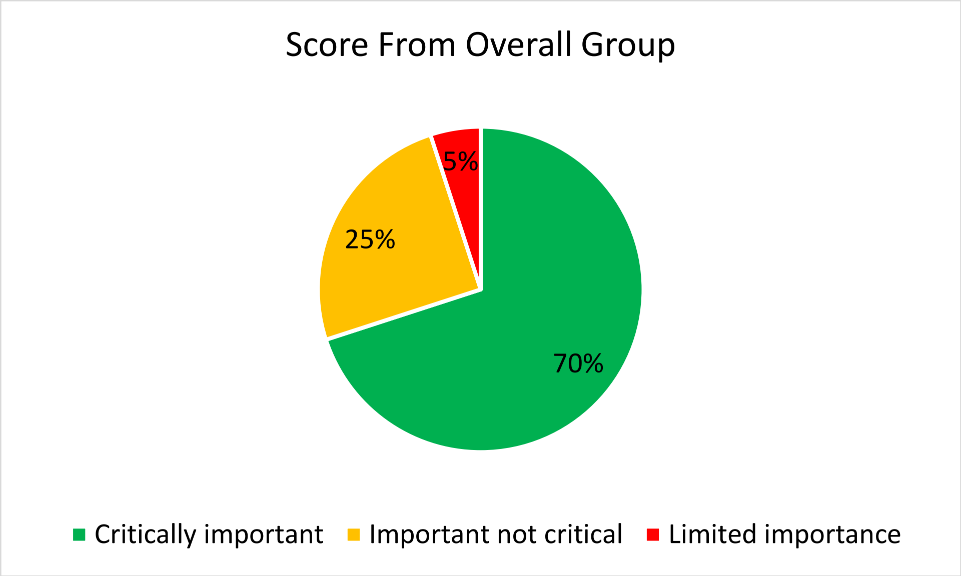

Scores From Each Stakeholder Group

Patients
Critically important: 100%
Important not critical: 0%
Limited importance: 0%

Researchers
Critically important: 59%
Important not critical: 41%
Limited importance: 0%

Clinicians
Critically important: 64%
Important not critical: 36%
Limited importance: 0%

Service-planners/Policymakers
Critically important: 71%
Important not critical: 0%
Limited importance: 21%

Reasons For Scores

Please note that the number in brackets shows the percentage of participants that gave that reason.

Reasons for including:
May be needed for cost-effectiveness analysis (2%).
Should be the ultimate purpose of the intervention (2%).
Critical information to persuade funders when implementing interventions into real-world healthcare settings (2%).

Number of recurrent fallers

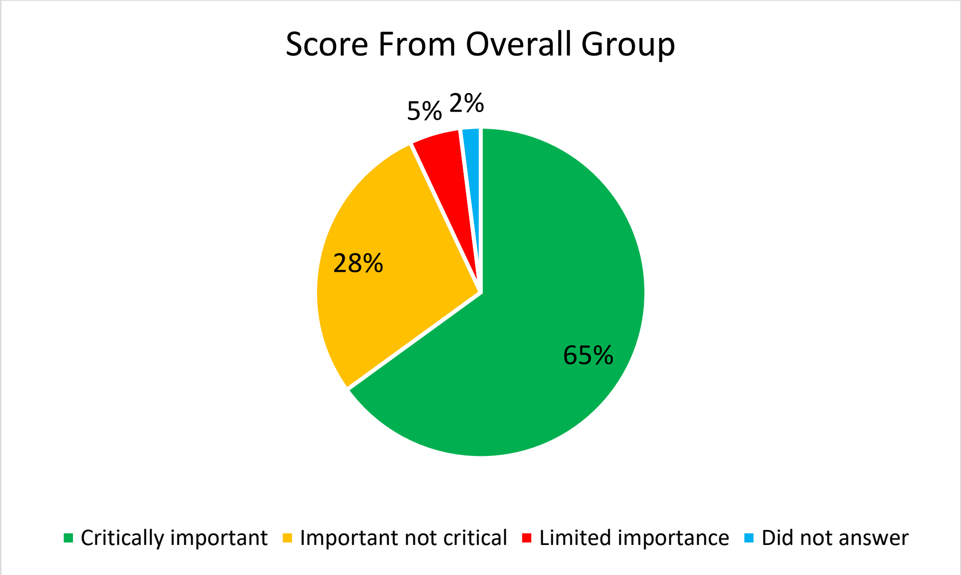

Scores From Each Stakeholder Group

Patients
Critically important: 50%
Important not critical: 37%
Limited importance: 0%
Did not answer: 13%

Researchers
Critically important: 76%
Important not critical: 12%
Limited importance: 12%

Clinicians
Critically important: 55%
Important not critical: 45%
Limited importance: 0%

Service-planners/Policymakers
Critically important: 71%
Important not critical: 29%
Limited importance: 0%

Reasons For Scores

Please note that the number in brackets shows the percentage of participants that gave that reason.

Reasons for including:
Direct and critical information to persuade funders when implementing programmes into real world healthcare settings (2%).
If a high number of participants are experiencing multiple falls then the intervention is not effective (2%).

Reasons for excluding:
Intervention may not reverse this (2%).

Self-efficacy

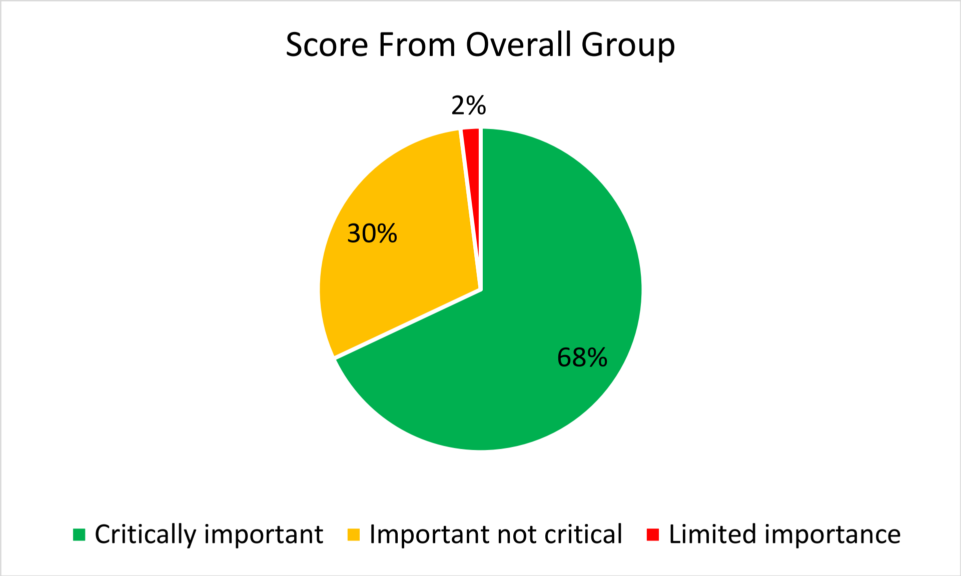

Scores From Each Stakeholder Group

Patients
Critically important: 75%
Important not critical: 25%
Limited importance: 0%

Researchers
Critically important: 59%
Important not critical: 35%
Limited importance: 6%

Clinicians
Critically important: 73%
Important not critical: 27%
Limited importance: 0%

Service-planners/Policymakers
Critically important: 71%
Important not critical: 29%
Limited importance: 0%

Reasons For Scores

Please note that the number in brackets shows the percentage of participants that gave that reason.

Reasons for including:
Important to patients (2%).
Low self-efficacy can affect goals and motivation for intervention (2%).

Reasons for excluding:
Outcome is not reliable (7%).
Self-belief cannot improve physical impairments (2%).
Concept of outcome is vague (2%).
Not related to the primary goal of the intervention (2%).

Falls self-management skills

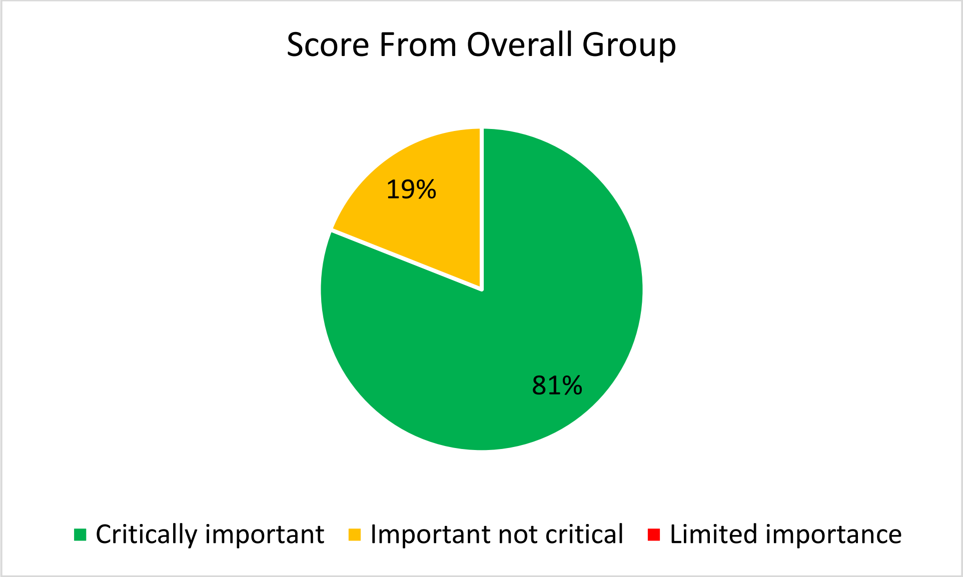

Scores From Each Stakeholder Group

Patients
Critically important: 87%
Important not critical: 13%
Limited importance: 0%

Researchers
Critically important: 82%
Important not critical: 18%
Limited importance: 0%

Clinicians
Critically important: 82%
Important not critical: 18%
Limited importance: 0%

Service-planners/Policymakers
Critically important: 71%
Important not critical: 29%
Limited importance: 0%

Self-reported ability to perform activities of daily living

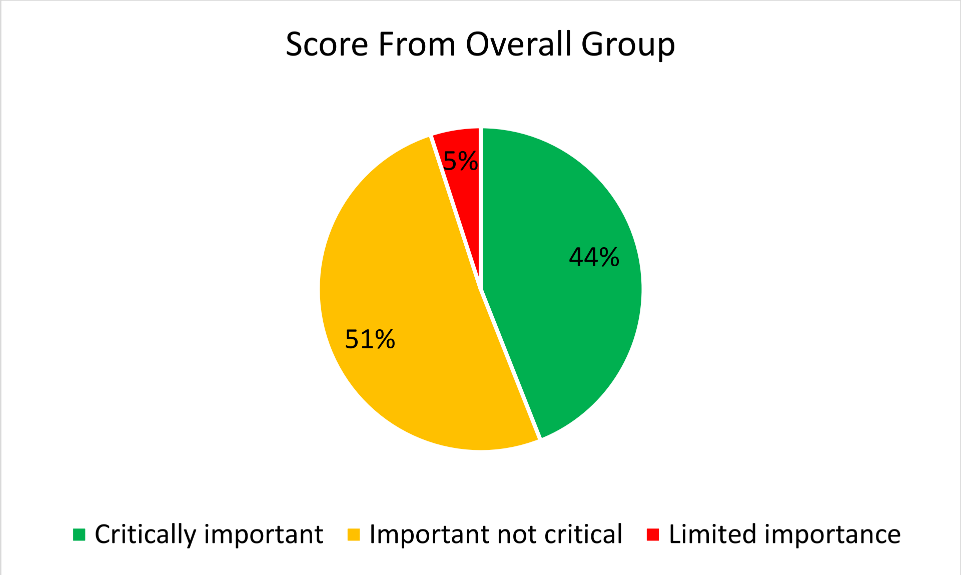

Scores From Each Stakeholder Group

Patients
Critically important: 62%
Important not critical: 38%
Limited importance: 0%

Researchers
Critically important: 24%
Important not critical: 70%
Limited importance: 6%

Clinicians
Critically important: 55%
Important not critical: 36%
Limited importance: 9%

Service-planners/Policymakers
Critically important: 57%
Important not critical: 43%
Limited importance: 0%

Reasons For Scores

Please note that the number in brackets shows the percentage of participants that gave that reason.

Reasons for including:
An individual's belief in their ability to complete activities of daily living will likely reflect their falls risk (2%).
Reported as important by patients in qualitative research (2%).
Gives an insight into the person's own self-awareness of their safety and their ability (2%).

Reasons for excluding:
Not related to the primary goal of the intervention (4%).
Completion of activities of daily living has many factors and the contents of a falls prevention intervention may not sufficiently address these and so this outcome may not change due to a falls prevention intervention (2%).

Self-reported mobility

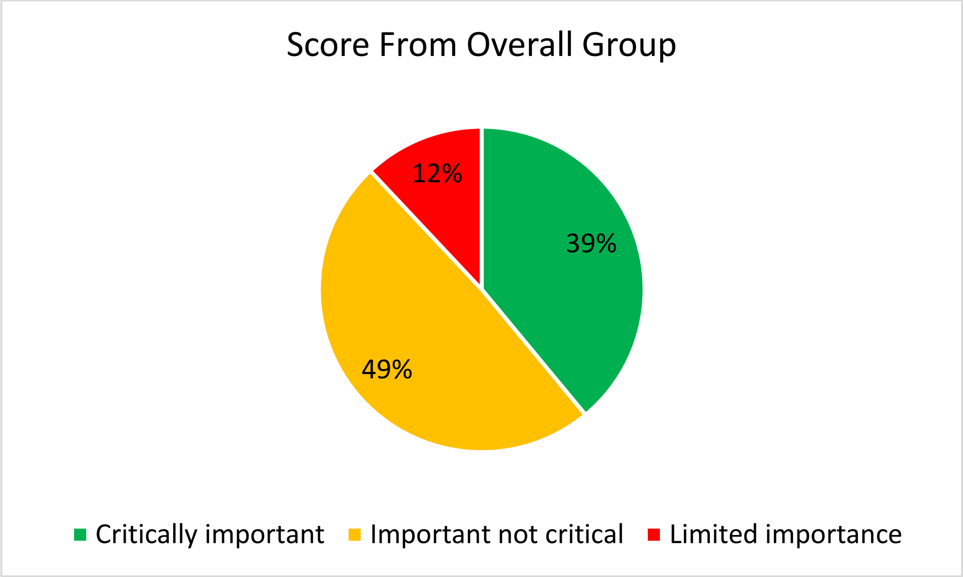

Scores From Each Stakeholder Group

Patients
Critically important: 62%
Important not critical: 38%
Limited importance: 0%

Researchers
Critically important: 24%
Important not critical: 47%
Limited importance: 29%

Clinicians
Critically important:27%
Important not critical: 73%
Limited importance: 0%

Service-planners/Policymakers
Critically important: 71%
Important not critical: 29%
Limited importance: 0%

Reasons For Scores

Please note that the number in brackets shows the percentage of participants that gave that reason.

<u>Reasons for excluding:</u>
Not related to the primary goal of the intervention (2%).
Unreliable outcome (2%).

Sleep quality

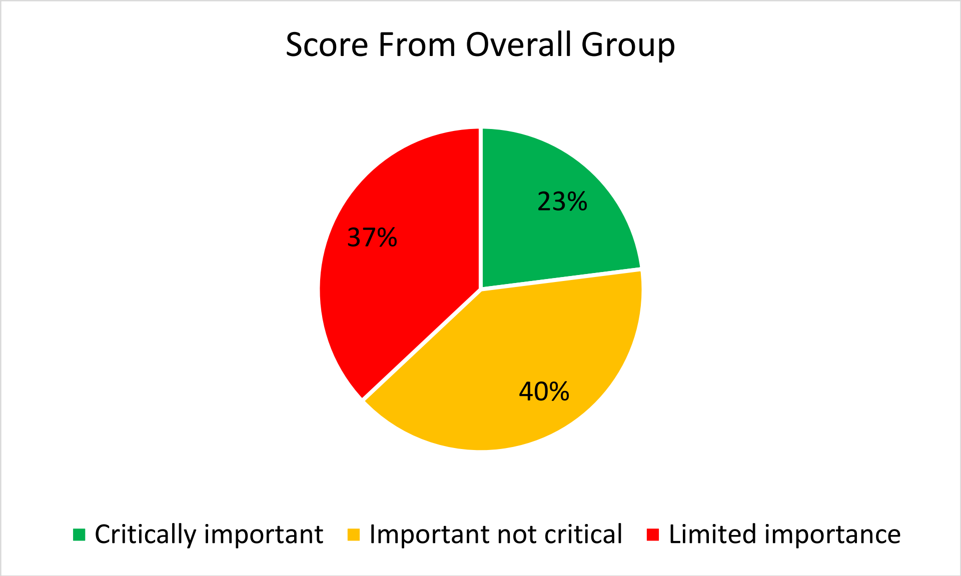

Scores From Each Stakeholder Group

Patients
Critically important: 50%
Important not critical: 50%
Limited importance: 0%

Researchers
Critically important: 0%
Important not critical: 35%
Limited importance: 65%

Clinicians
Critically important: 18%
Important not critical: 46%
Limited importance: 36%

Service-planners/Policymakers
Critically important: 57%
Important not critical: 29%
Limited importance: 14%

Reasons For Scores

Please note that the number in brackets shows the percentage of participants that gave that reason.

Reasons for excluding:
Not related to the primary goal of the intervention (7%).

Ability to engage in social activities

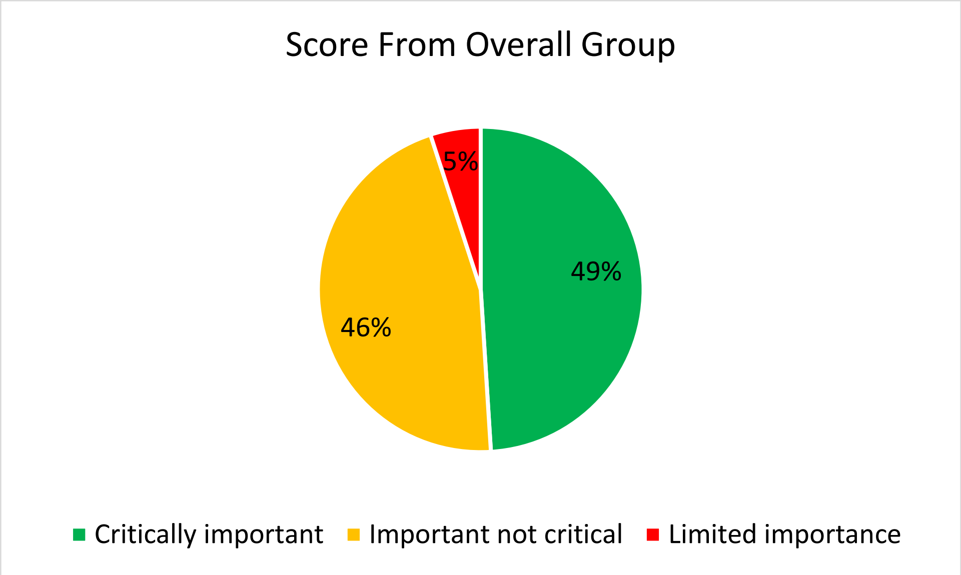

Scores From Each Stakeholder Group

Patients
Critically important: 87%
Important not critical: 13%
Limited importance: 0%

Researchers
Critically important: 35%
Important not critical: 53%
Limited importance: 12%

Clinicians
Critically important:36%
Important not critical: 64%
Limited importance: 0%

Service-planners/Policymakers
Critically important: 57%
Important not critical: 43%
Limited importance: 0%

Reasons For Scores

Please note that the number in brackets shows the percentage of participants that gave that reason.

Reasons for including:
Impacts on overall physical and mental wellbeing (4%).
Can be a motivational factor for patients (2%).
Social interaction is an important holistic goal (2%).

Reasons for excluding:
Difficult to measure (2%).
Social activities may not be related to falls (2%).
Not related to the primary goal of the intervention (2%).

Static balance

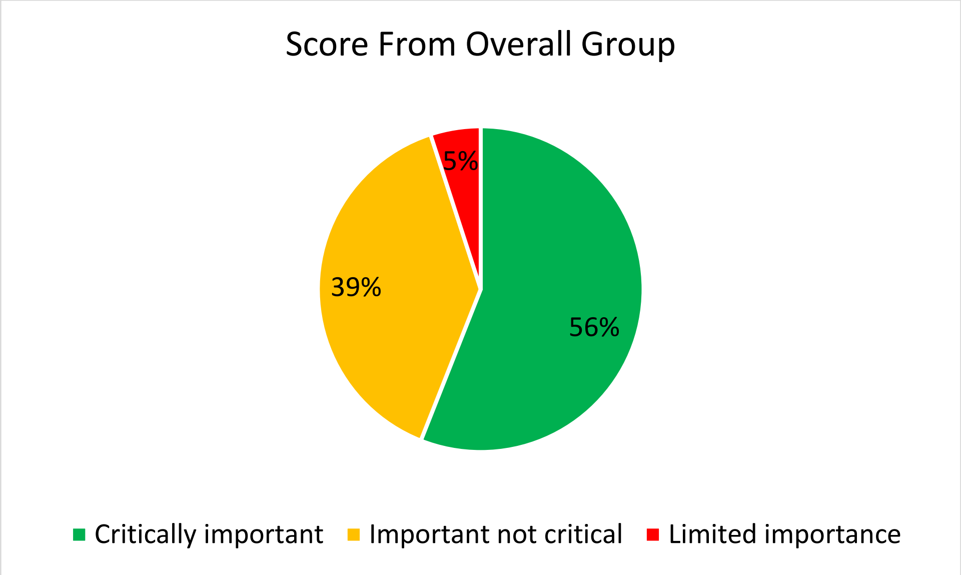

Scores From Each Stakeholder Group

Patients
Critically important: 100%
Important not critical: 0%
Limited importance: 0%

Researchers
Critically important: 18%
Important not critical: 70%
Limited importance: 12%

Clinicians
Critically important: 55%
Important not critical: 45%
Limited importance: 0%

Service-planners/Policymakers
Critically important: 100%
Important not critical: 0%
Limited importance: 0%

Reasons For Scores

Please note that the number in brackets shows the percentage of participants that gave that reason.

<u>Reasons for including:</u>
Indication of overall balance ability (2%).
Can link with patients' fears and confidence (2%).
Important building block to balance in functional/dynamic activities (2%).
Falls can occur from a loss of static balance (2%).

<u>Reasons for excluding:</u>
Not as important as dynamic balance as falls mostly occur with movement (9%).
Not relevant to all interventions only those where they goal is to prevent falls by improving balance control (2%).

Stride length

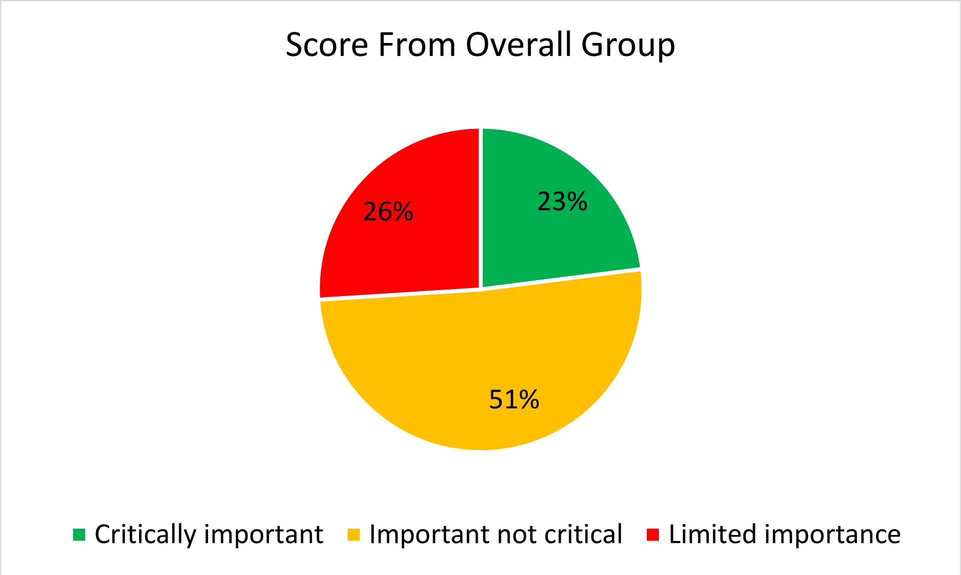

Scores From Each Stakeholder Group

Patients
Critically important: 38%
Important not critical: 62%
Limited importance: 0%

Researchers
Critically important: 6%
Important not critical: 41%
Limited importance: 53%

Clinicians
Critically important: 27%
Important not critical: 55%
Limited importance: 18%

Service-planners/Policymakers
Critically important: 43%
Important not critical: 57%
Limited importance: 0%

Reasons For Scores

Please note that the number in brackets shows the percentage of participants that gave that reason.

Reasons for including:
Affects toe push off, efficiency of gait and, thus, falls risk (2%).

Reasons for excluding:
Not related to the primary goal of the intervention (2%).

Time to first post-intervention fall

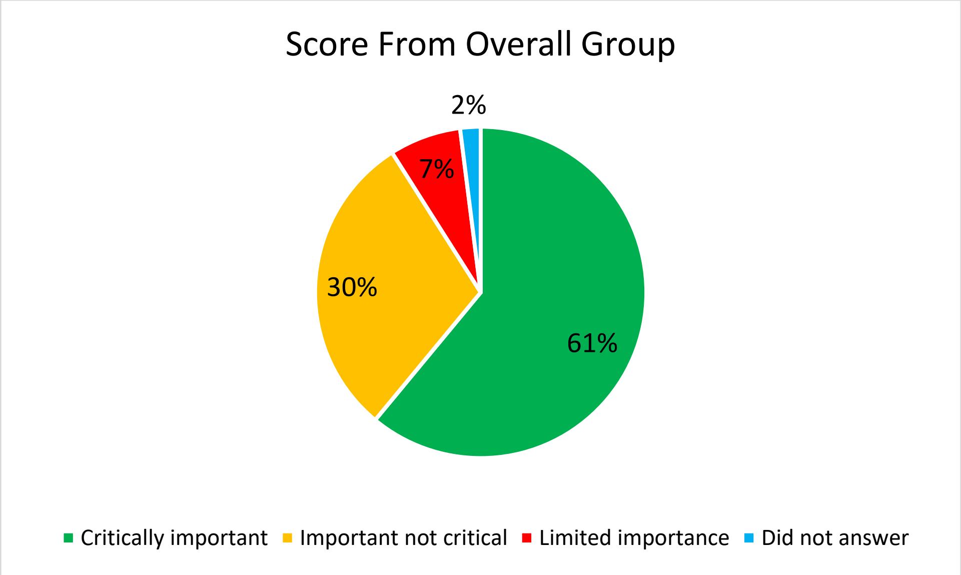

Scores From Each Stakeholder Group

Patients
Critically important: 50%
Important not critical: 25%
Limited importance: 13%
Did not answer: 12%

Researchers
Critically important: 65%
Important not critical: 35%
Limited importance: 0%

Clinicians
Critically important: 45%
Important not critical: 45%
Limited importance: 10%

Service-planners/Policymakers
Critically important: 86%
Important not critical: 0%
Limited importance: 14%

Reasons For Scores

Please note that the number in brackets shows the percentage of participants that gave that reason.

Reasons for including:
Important when measuring the cost/benefit of the intervention (4%).
May show length of treatment effect and compliance with home exercise programme (2%).

Reasons for excluding:
Outcome is saying intervention is delaying the inevitable – falls per person over follow-up is a better way of investigating this (2%).

Understanding of personal falls risk factors

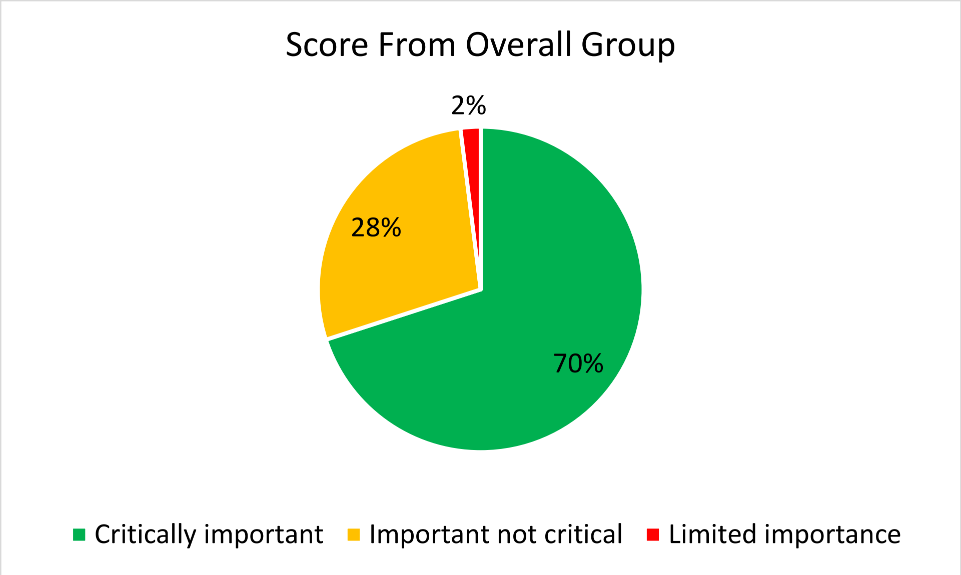

Scores From Each Stakeholder Group

Patients
Critically important: 100%
Important not critical: 0%
Limited importance: 0%

Researchers
Critically important: 59%
Important not critical: 35%
Limited importance: 6%

Clinicians
Critically important: 55%
Important not critical: 45%
Limited importance: 0%

Service-planners/Policymakers
Critically important: 86%
Important not critical: 14%
Limited importance: 0%

Reasons For Scores

Please note that the number in brackets shows the percentage of participants that gave that reason.

Reasons for including:
May facilitate self-management (4%).
Understanding risk is essential to prevent falls (2%).

Walking distance

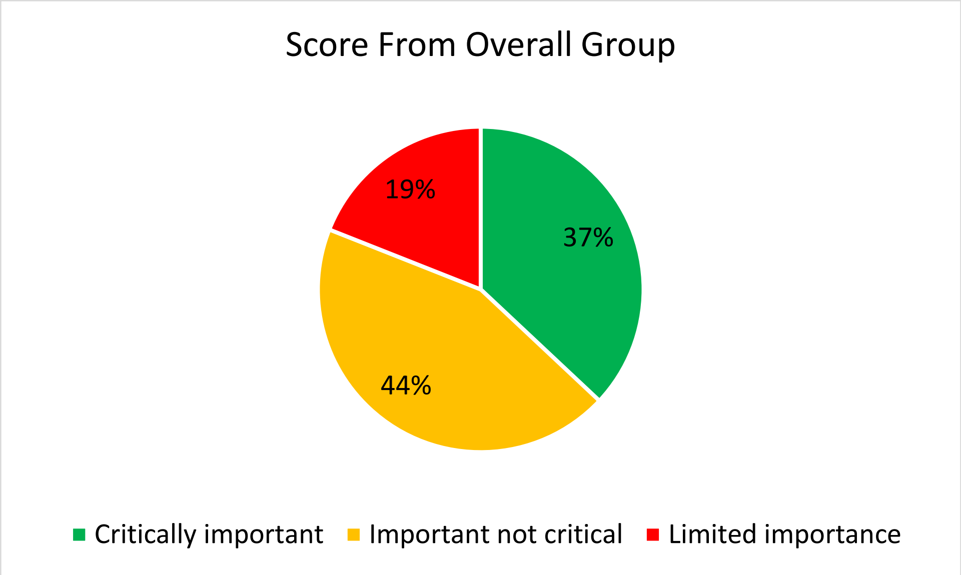

Scores From Each Stakeholder Group

Patients
Critically important: 62%
Important not critical: 38%
Limited importance: 0%

Researchers
Critically important: 18%
Important not critical: 41%
Limited importance: 41%

Clinicians
Critically important: 27%
Important not critical: 64%
Limited importance: 9%

Service-planners/Policymakers
Critically important: 71%
Important not critical: 29%
Limited importance: 0%

Reasons For Scores

Please note that the number in brackets shows the percentage of participants that gave that reason.

Reasons for excluding:
Not relevant to all interventions only those aiming to prevent falls by improving walking endurance (2%).
Not related to the primary goal of the intervention (2%).

Walking self-efficacy

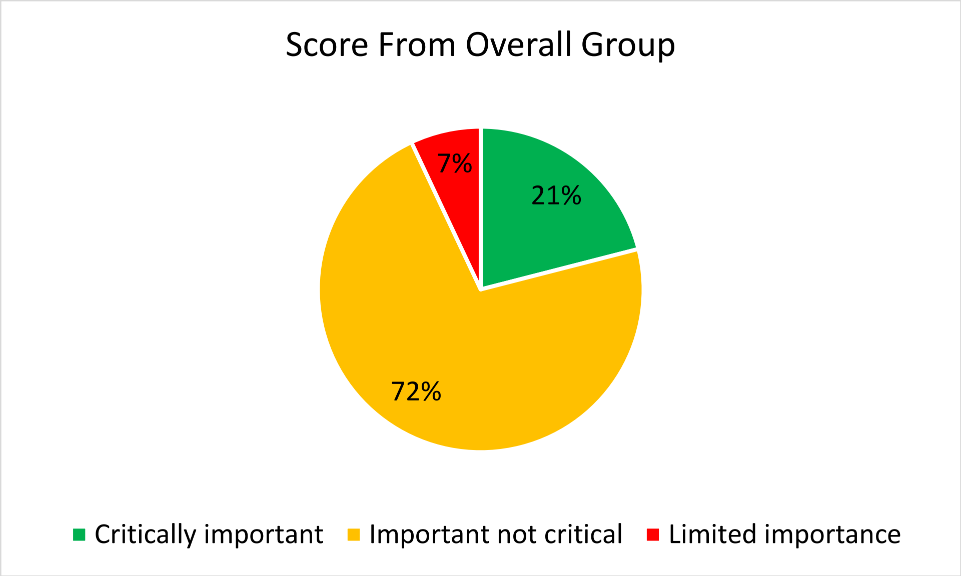

Scores From Each Stakeholder Group

Patients
Critically important: 25%
Important not critical: 75%
Limited importance: 0%

Researchers
Critically important: 12%
Important not critical: 70%
Limited importance: 18%

Clinicians
Critically important: 18%
Important not critical: 82%
Limited importance: 0%

Service-planners/Policymakers
Critically important: 43%
Important not critical: 57%
Limited importance: 0%

Reasons For Scores

Please note that the number in brackets shows the percentage of participants that gave that reason.

Reasons for excluding:
Not related to the primary goal of the intervention (4%).
May not be a reliable outcome (2%).
Higher belief in ability can leading to higher risk taking (2%).

Walking speed

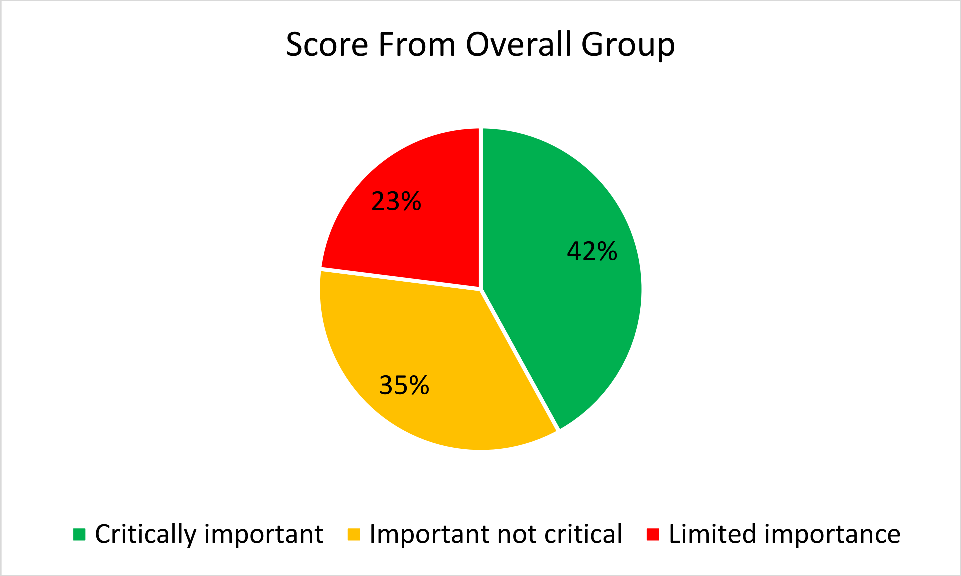

Scores From Each Stakeholder Group

Patients
Critically important: 50%
Important not critical: 25%
Limited importance: 25%

Researchers
Critically important: 30%
Important not critical: 35%
Limited importance: 35%

Clinicians
Critically important: 46%
Important not critical: 36%
Limited importance: 18%

Service-planners/Policymakers
Critically important: 57%
Important not critical: 43%
Limited importance: 0%

Reasons For Scores

Please note that the number in brackets shows the percentage of participants that gave that reason.

Reasons for including:
Can indicate falls risk (2%).

Reasons for excluding:
Not related to risk of falling (4%).
Quality of movement rather than speed may be of more significance (2%).
Reduced walking speed may be a positive outcome for some patients (2%).
Not related to the primary goal of the intervention (2%).

Falls rate adjusted for activity exposure

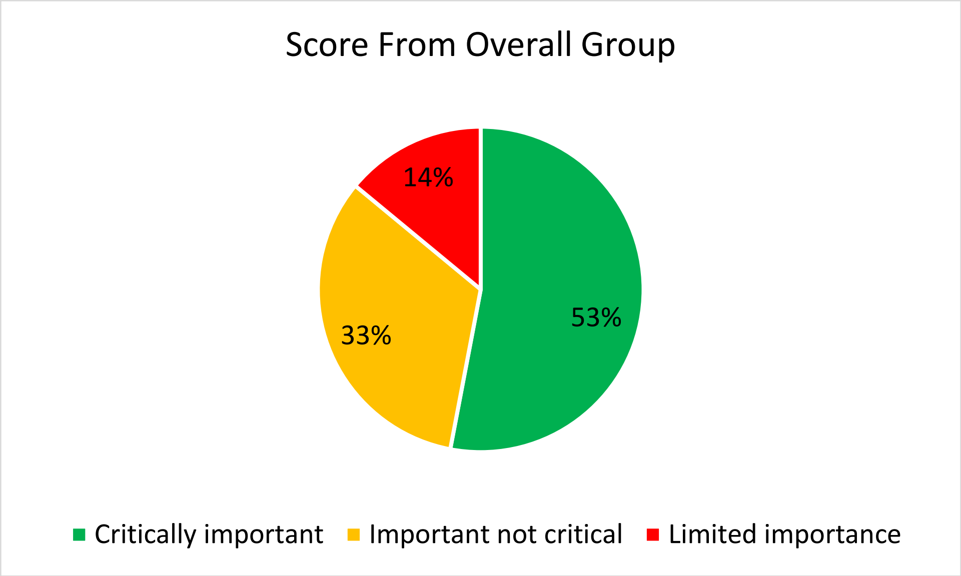

Scores From Each Stakeholder Group

Patients
Critically important: 75%
Important not critical: 0%
Limited importance: 25%

Researchers
Critically important: 59%
Important not critical: 35%
Limited importance: 6%

Clinicians
Critically important: 36%
Important not critical: 55%
Limited importance: 9%

Service-planners/Policymakers
Critically important: 43%
Important not critical: 29%
Limited importance: 28%

Reasons For Scores

Please note that the number in brackets shows the percentage of participants that gave that reason.

Reasons for including:
Important to look at falls rate relative to participation (2%).
Could be used to account for disease severity and mobility (2%).

Reasons for excluding:
Subject to memory/recall of patients (4%).
Disease severity will influence the relevance of this outcome (2%).
May be difficult to quantify (2%).
Physical activity measures are unreliable and dividing number of falls by an unreliable measure introduces error into estimates of number of falls – better to use physical activity as a co-variate in analysis (2%).

Time spent out of bed during daytime

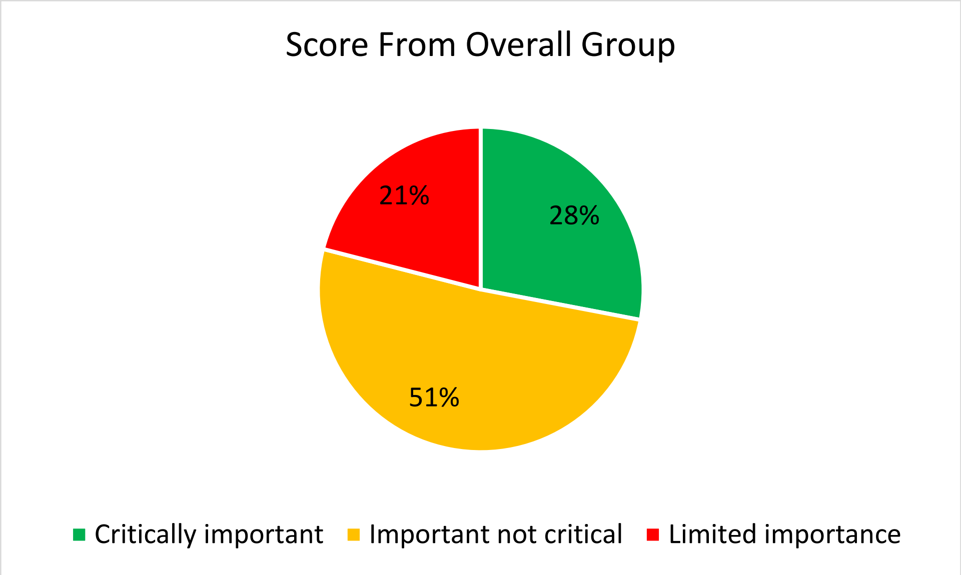

Scores From Each Stakeholder Group

Patients
Critically important: 62%
Important not critical: 25%
Limited importance: 13%

Researchers
Critically important: 12%
Important not critical: 53%
Limited importance: 35%

Clinicians
Critically important: 27%
Important not critical: 64%
Limited importance: 9%

Service-planners/Policymakers
Critically important: 29%
Important not critical: 57%
Limited importance: 14%

Reasons For Scores

Please note that the number in brackets shows the percentage of participants that gave that reason.

Reasons for including:
Reduces risk of sarcopenia (2%).

Reasons for excluding:
Unlikely to improve with a falls prevention intervention (4%).

Joining a support/community group

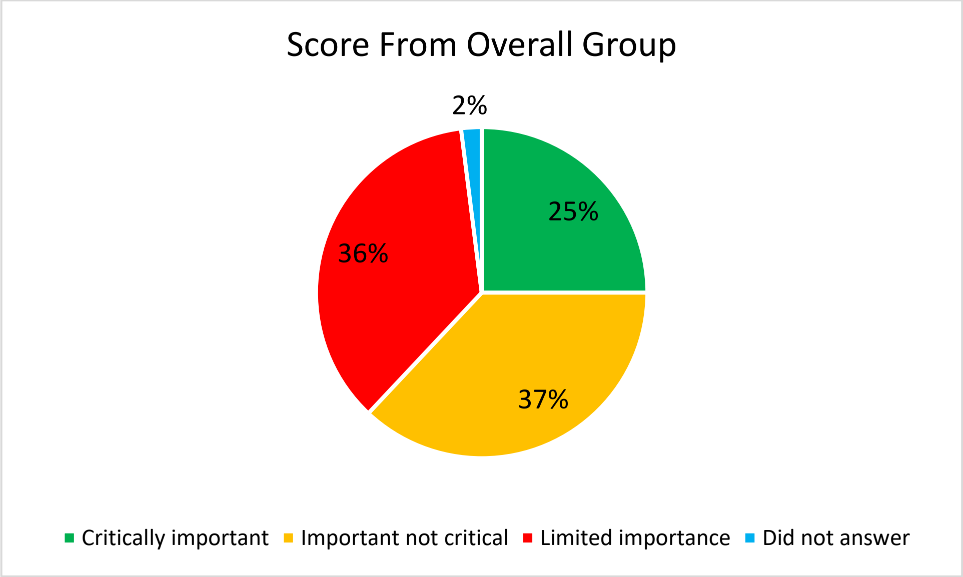

Scores From Each Stakeholder Group

Patients
Critically important: 37%
Important not critical: 38%
Limited importance: 13%
Did not answer: 12%

Researchers
Critically important: 18%
Important not critical: 23%
Limited importance: 59%

Clinicians
Critically important: 18%
Important not critical: 64%
Limited importance: 18%

Service-planners/Policymakers
Critically important: 42%
Important not critical: 29%
Limited importance: 29%

Reasons For Scores

Please note that the number in brackets shows the percentage of participants that gave that reason.

Reasons for excluding:
Not related to the primary goal of the intervention (4%).
Not always possible/desired by all participants (4%).

Changes to home/work environment

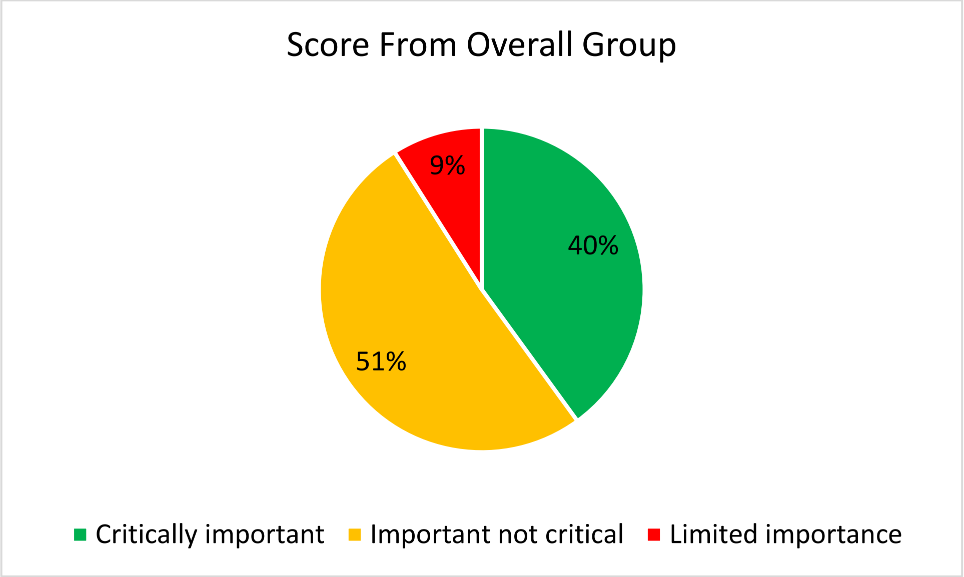

Scores From Each Stakeholder Group

Patients
Critically important: 62%
Important not critical: 38%
Limited importance: 0%

Researchers
Critically important: 29%
Important not critical: 59%
Limited importance: 12%

Clinicians
Critically important: 18%
Important not critical: 73%
Limited importance: 9%

Service-planners/Policymakers
Critically important: 72%
Important not critical: 14%
Limited importance: 14%

Reasons For Scores

Please note that the number in brackets shows the percentage of participants that gave that reason.

Reasons for including:
Optimises function in a safe environment (2%).

Reasons for excluding:
Not relevant to all interventions only those aiming to prevent falls by environmental modifications (7%).
Subject to budget limitations (2%).
Not relevant to all participants (2%).
Not related to the primary goal of the intervention (2%).
