# Supplementary material for: A core outcome set for evaluating the effectiveness of mixed-diagnosis falls prevention interventions for people with Multiple Sclerosis, Parkinson’s Disease and stroke
Source: PLoS One. 2023 Nov 13;18(11):e0294193. doi: 10.1371/journal.pone.0294193 (PMC10642845; doi:10.1371/journal.pone.0294193)
Supplement: S5 Appendix — (PDF) [file pone.0294193.s005.pdf]

Appendix 5: Summary of survey responses from round three.

Cost-effectiveness

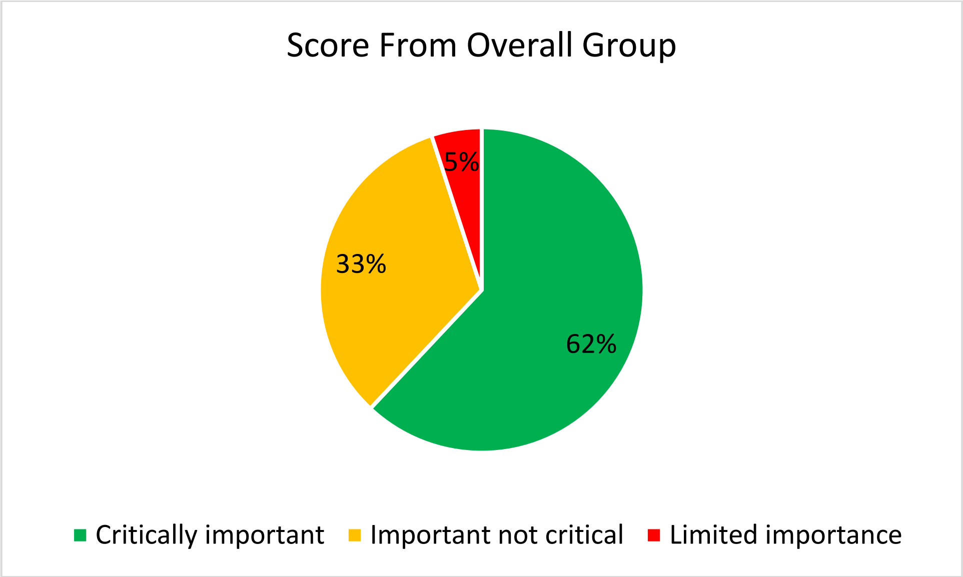

Scores From Each Stakeholder Group

Patients
Critically important: 74%
Important not critical: 13%
Limited importance: 13%

Researchers
Critically important: 88%
Important not critical: 12%
Limited importance: 0%

Clinicians
Critically important: 18%
Important not critical: 82%
Limited importance: 0%

Service-planners/Policymakers
Critically important: 57%
Important not critical: 29%
Limited importance: 14%

Reasons For Scores

Please note that the number in brackets shows the percentage of participants that gave that reason.

<u>Reasons for including:</u>
Important information for funders to implement evidence-based interventions into real-life healthcare settings (7%).
Important in deciding between multiple interventions (2%).
Needed to motivate providers to deliver evidence-based interventions (2%).
If an intervention is not found to be cost-effective it will be discontinued (2%).
Resources are finite and should be distributed in a transparent and accountable manner to achieve the best outcomes (2%).

Number of fallers

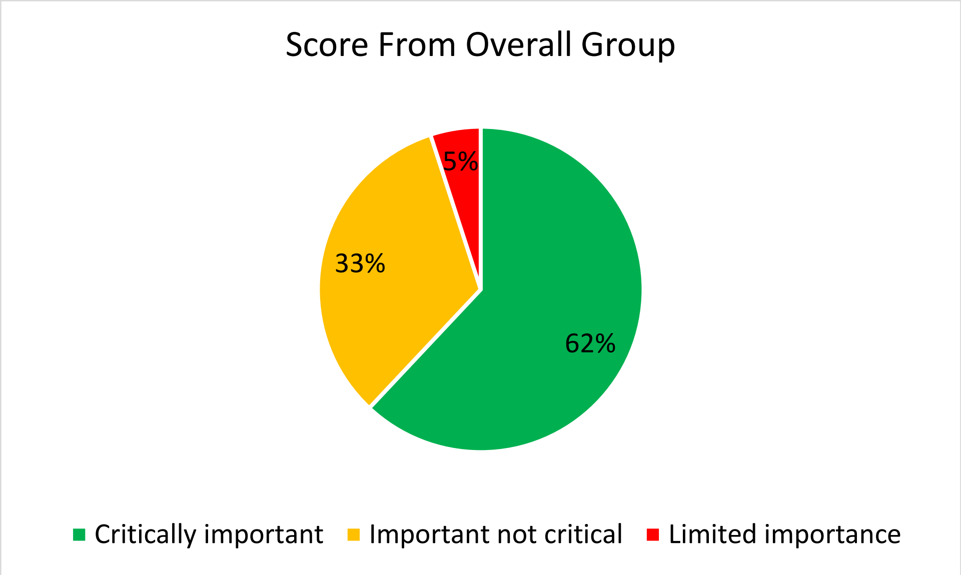

Scores From Each Stakeholder Group

Patients
Critically important: 62%
Important not critical: 25%
Limited importance: 13%

Researchers
Critically important: 75%
Important not critical: 19%
Limited importance: 6%

Clinicians
Critically important: 45%
Important not critical: 55%
Limited importance: 0%

Service-planners/Policymakers
Critically important: 57%
Important not critical: 43%
Limited importance: 0%

Reasons For Scores

Please note that the number in brackets shows the percentage of participants that gave that reason.

<u>Reasons for excluding:</u>
You can reduce falls but cannot eradicate them completely (2%).

Falls risk

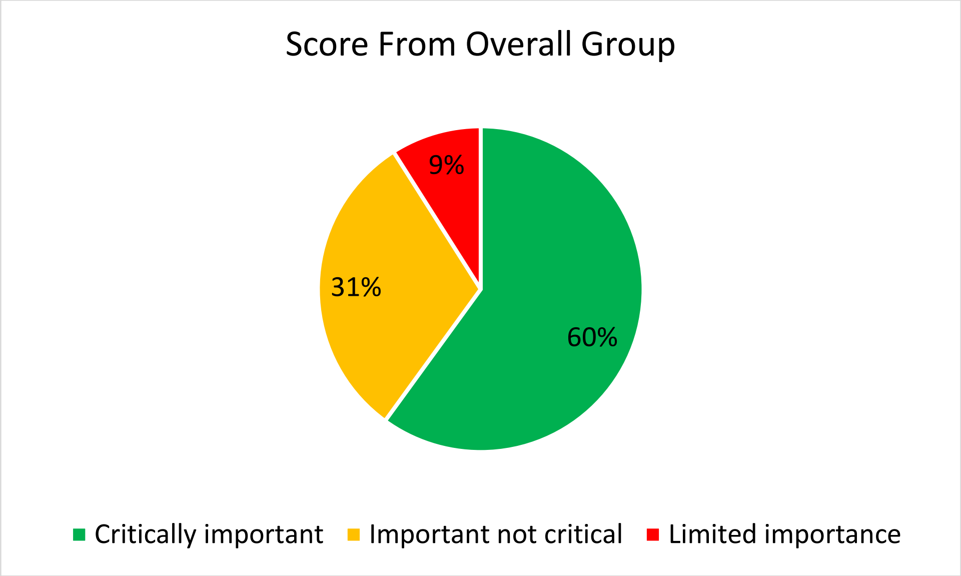

Scores From Each Stakeholder Group

Patients
Critically important: 74%
Important not critical: 13%
Limited importance: 13%

Researchers
Critically important: 31%
Important not critical: 56%
Limited importance: 13%

Clinicians
Critically important: 64%
Important not critical: 27%
Limited importance: 9%

Service-planners/Policymakers
Critically important: 100%
Important not critical: 0%
Limited importance: 0%

Reasons For Scores

Please note that the number in brackets shows the percentage of participants that gave that reason.

<u>Reasons for excluding:</u>
Some risk factors for falls are non-reversible (2%).
Unclear how this differs from actual fall rates in daily life (2%).
Risk of falling is difficult to measure (2%).

Falls self-efficacy

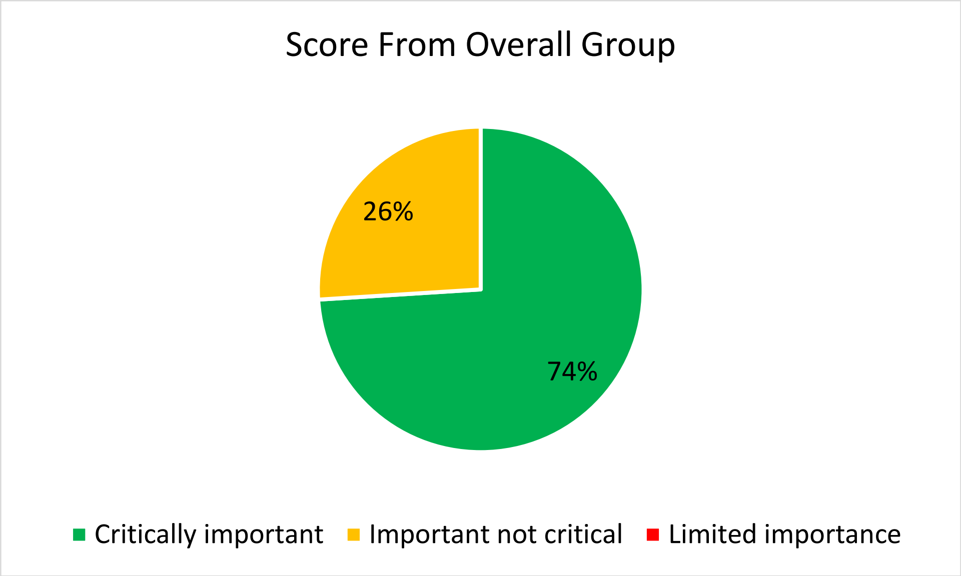

Scores From Each Stakeholder Group

Patients
Critically important: 75%
Important not critical: 25%
Limited importance: 0%

Researchers
Critically important: 75%
Important not critical: 25%
Limited importance: 0%

Clinicians
Critically important: 73%
Important not critical: 27%
Limited importance: 0%

Service-planners/Policymakers
Critically important: 71%
Important not critical: 29%
Limited importance: 0%

Reasons For Scores

Please note that the number in brackets shows the percentage of participants that gave that reason.

Reasons for including:
Reduced falls self-efficacy, and subsequent reduction in activity, may increase risk of falls (7%).
Reduced falls self-efficacy may prevent an individual from carrying out activities of daily living (4%).
The FES-I, which measures falls self-efficacy, has been identified through consensus as an important outcome measure for falls prevention trials (2%).

Knowledge of how to get up from the floor after a fall

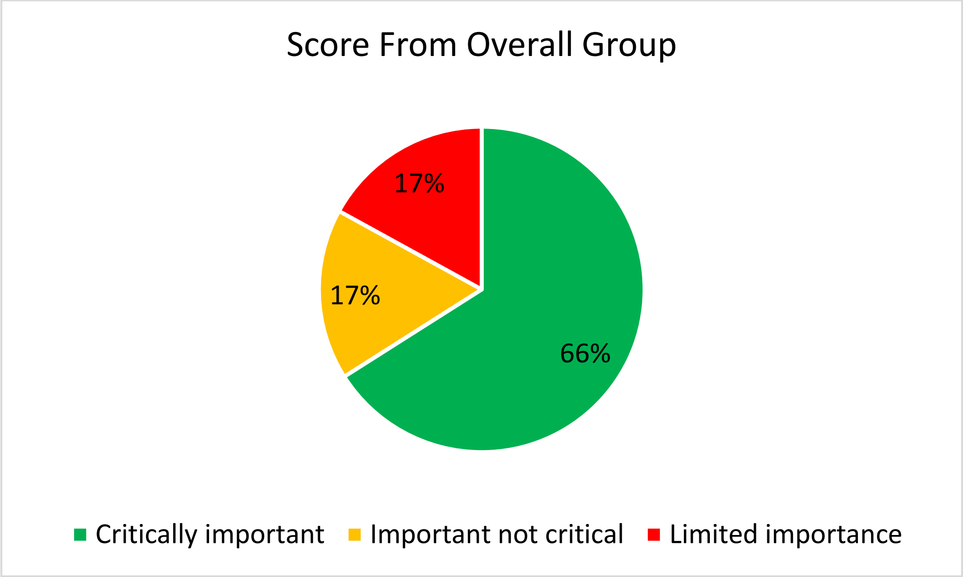

Scores From Each Stakeholder Group

Patients
Critically important: 87%
Important not critical: 13%
Limited importance: 0%

Researchers
Critically important: 37%
Important not critical: 25%
Limited importance: 38%

Clinicians
Critically important: 82%
Important not critical: 9%
Limited importance: 9%

Service-planners/Policymakers
Critically important: 86%
Important not critical: 14%
Limited importance: 0%

Reasons For Scores

Please note that the number in brackets shows the percentage of participants that gave that reason.

Reasons for including:
May reduce fear of falling (4%).
Good to have knowledge even if ability to so is limited (2%).
Knowing how to get up safely may reduce injuries (2%).
Will improve planning and awareness (2%).

Reasons for excluding:
May not be possible for/applicable to all participants (4%).
Ability to get up from the floor more important than knowledge of how to (2%).

Ability to independently perform activities of daily living

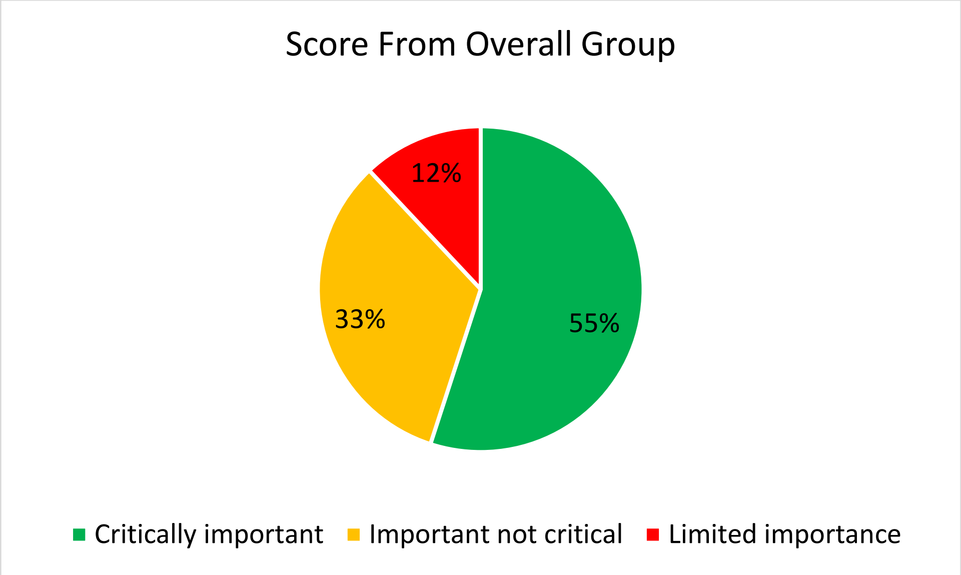

Scores From Each Stakeholder Group

Patients
Critically important: 75%
Important not critical: 25%
Limited importance: 0%

Researchers
Critically important: 31%
Important not critical: 44%
Limited importance: 25%

Clinicians
Critically important: 55%
Important not critical: 36%
Limited importance: 9%

Service-planners/Policymakers
Critically important: 86%
Important not critical: 14%
Limited importance: 0%

Reasons For Scores

Please note that the number in brackets shows the percentage of participants that gave that reason.

Reasons for excluding:
Directing others in care/receiving assistance may be an important falls prevention strategy for some individuals (2%).
Not related to the primary goal of the intervention (2%).
If help is available to a patient this may not be an issue (2%).
This outcome has subjective components making it difficult to measure (2%).

Level of physical activity

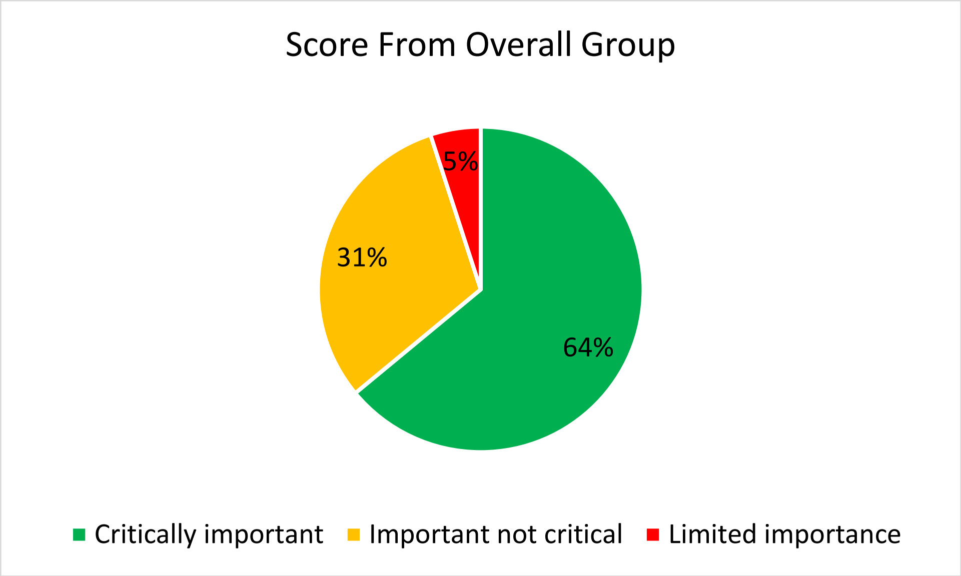

Scores From Each Stakeholder Group

Patients
Critically important: 75%
Important not critical: 25%
Limited importance: 0%

Researchers
Critically important: 44%
Important not critical: 44%
Limited importance: 12%

Clinicians
Critically important: 64%
Important not critical: 36%
Limited importance: 0%

Service-planners/Policymakers
Critically important: 100%
Important not critical: 0%
Limited importance: 0%

Reasons For Scores

Please note that the number in brackets shows the percentage of participants that gave that reason.

<u>Reasons for including:</u>
A person who is inactive may not have any falls but this does not indicate that an intervention is working (2%).
Activity reduces fear of falling, and maintains strength, mood, and mobility (2%).

<u>Reasons for excluding:</u>
Increased physical activity does not always reduce falls (2%).

Lower limb strength

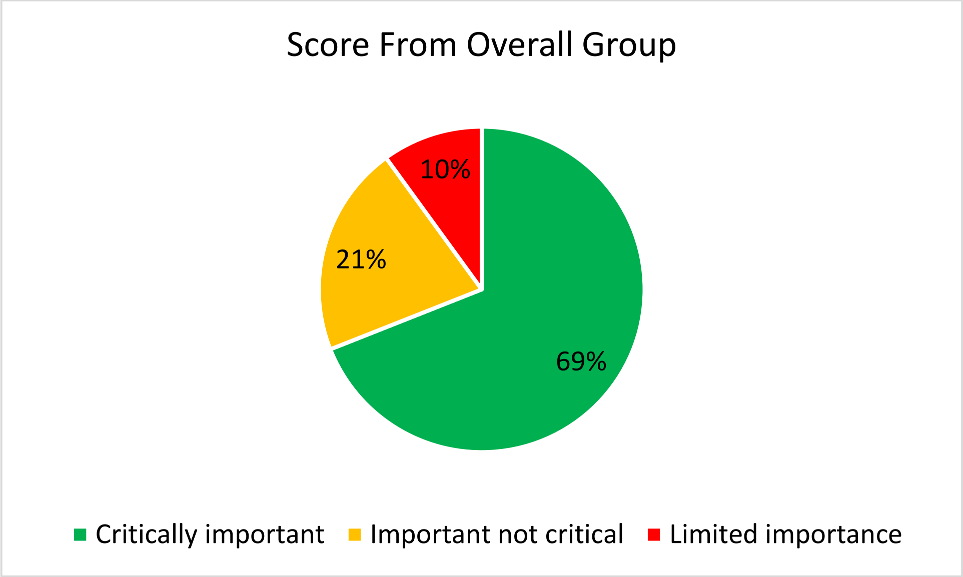

Scores From Each Stakeholder Group

Patients
Critically important: 75%
Important not critical: 25%
Limited importance: 0%

Researchers
Critically important: 50%
Important not critical: 25%
Limited importance: 25%

Clinicians
Critically important: 73%
Important not critical: 27%
Limited importance: 0%

Service-planners/Policymakers
Critically important: 100%
Important not critical: 0%
Limited importance: 0%

Reasons For Scores

Please note that the number in brackets shows the percentage of participants that gave that reason.

Reasons for including:
Important for safe ambulation (2%).
Important for balance (2%).

Reasons for excluding:
Not consistently emerging as a risk factor for falls (2%).
Not relevant to all interventions only those aiming to reduce falls by improving lower limb strength (2%).

Number of falls resulting in a long lie

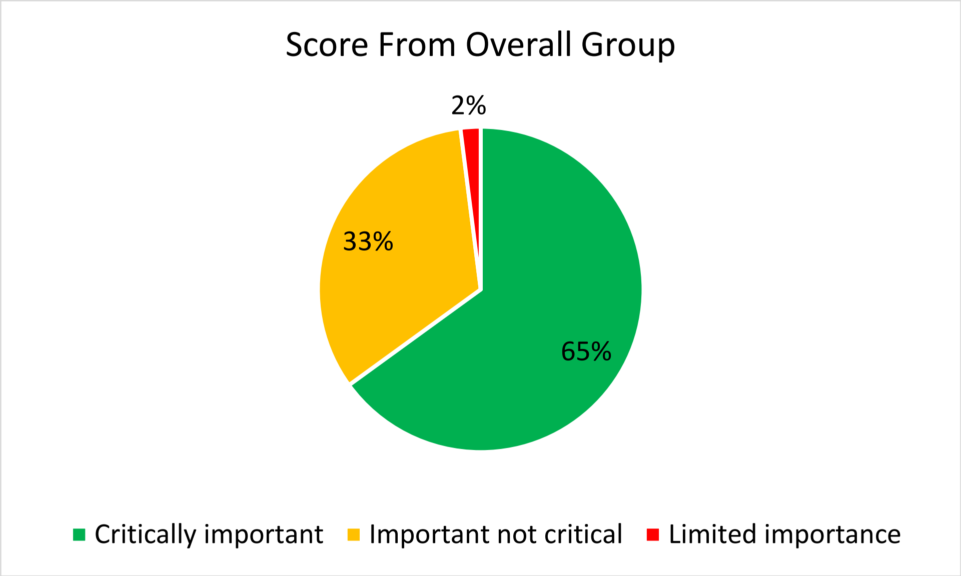

Scores From Each Stakeholder Group

Patients
Critically important: 62%
Important not critical: 25%
Limited importance: 13%

Researchers
Critically important: 50%
Important not critical: 50%
Limited importance: 0%

Clinicians
Critically important: 64%
Important not critical: 36%
Limited importance: 0%

Service-planners/Policymakers
Critically important: 100%
Important not critical: 0%
Limited importance: 0%

Reasons For Scores

Please note that the number in brackets shows the percentage of participants that gave that reason.

Reasons for including:
Indication of muscle strength, plans for falls and social isolation (2%).

Objectively assessed ability perform activities of daily living

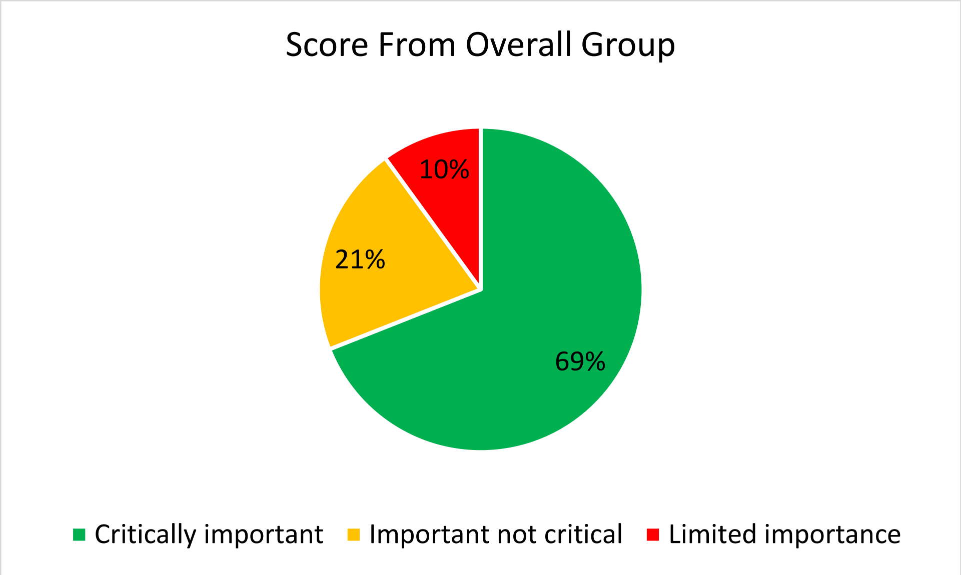

Scores From Each Stakeholder Group

Patients
Critically important: 100%
Important not critical: 0%
Limited importance: 0%

Researchers
Critically important: 44%
Important not critical: 37%
Limited importance: 19%

Clinicians
Critically important: 73%
Important not critical: 18%
Limited importance: 9%

Service-planners/Policymakers
Critically important: 86%
Important not critical: 14%
Limited importance: 0%

Reasons For Scores

Please note that the number in brackets shows the percentage of participants that gave that reason.

Reasons for including:
Although improving activities of daily living may not be the aim of the intervention, it could be argued that falls could be reduced through further restricting participation/activities which would not be a desirable outcome (2%).
Participants most likely to fall while they are completing an activity (2%).
If poor strength and/or balance is reducing their ability to perform activities, this may improve with intervention (2%).

Reasons for excluding:
Not related to the primary goal of the intervention (2%).
Unlikely to improve with a falls prevention intervention (2%).

Objectively assessed mobility

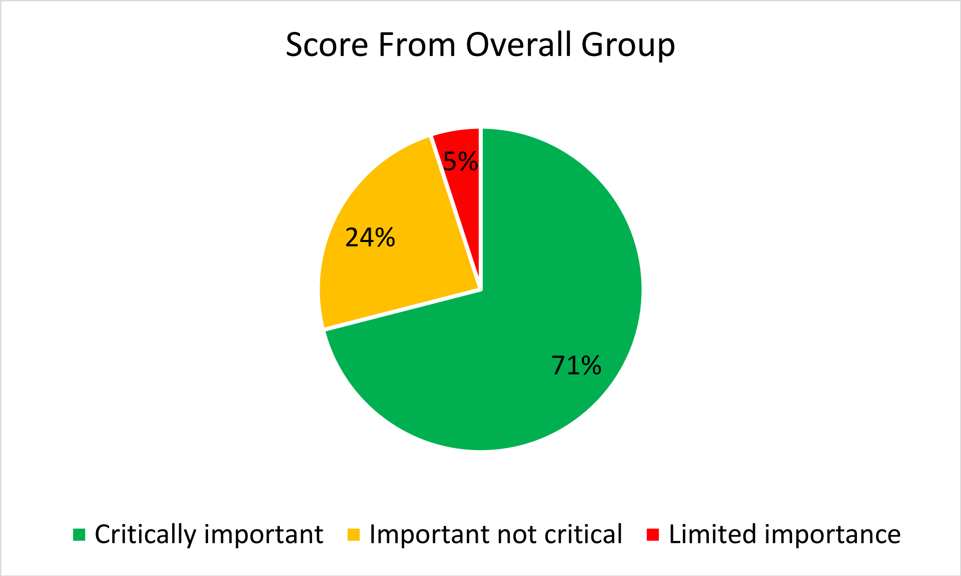

Scores From Each Stakeholder Group

Patients
Critically important: 87%
Important not critical: 13%
Limited importance: 0%

Researchers
Critically important: 82%
Important not critical: 18%
Limited importance: 0%

Clinicians
Critically important: 100%
Important not critical: 0%
Limited importance: 0%

Service-planners/Policymakers
Critically important: 100%
Important not critical: 0%
Limited importance: 0%

Reasons For Scores

Please note that the number in brackets shows the percentage of participants that gave that reason.

Reasons for including:
Important for independence and safety (2%).
Linked to fear of falling (2%).

Reasons for excluding:
Not related to the primary goal of the intervention (4%).
Not relevant to all participants (2%).

Perceived control of falls

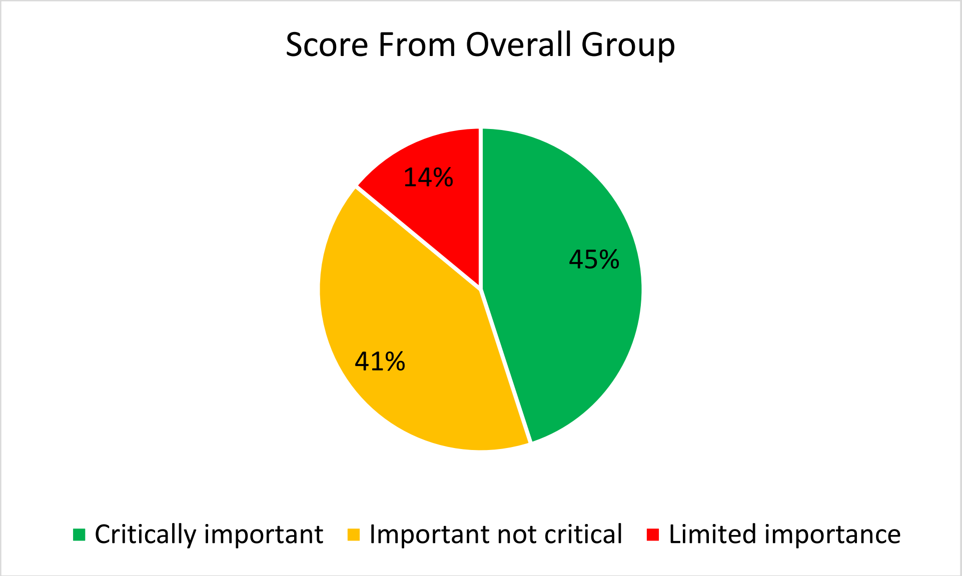

Scores From Each Stakeholder Group

Patients
Critically important: 62%
Important not critical: 38%
Limited importance: 0%

Researchers
Critically important: 44%
Important not critical: 25%
Limited importance: 31%

Clinicians
Critically important: 27%
Important not critical: 64%
Limited importance: 9%

Service-planners/Policymakers
Critically important: 57%
Important not critical: 43%
Limited importance: 0%

Reasons For Scores

Please note that the number in brackets shows the percentage of participants that gave that reason.

Reasons for including:
Encourages positive risk-taking and participation (4%).
If an individual feels they have control over falling, it will increase their activity levels (2%).

Number of recurrent fallers

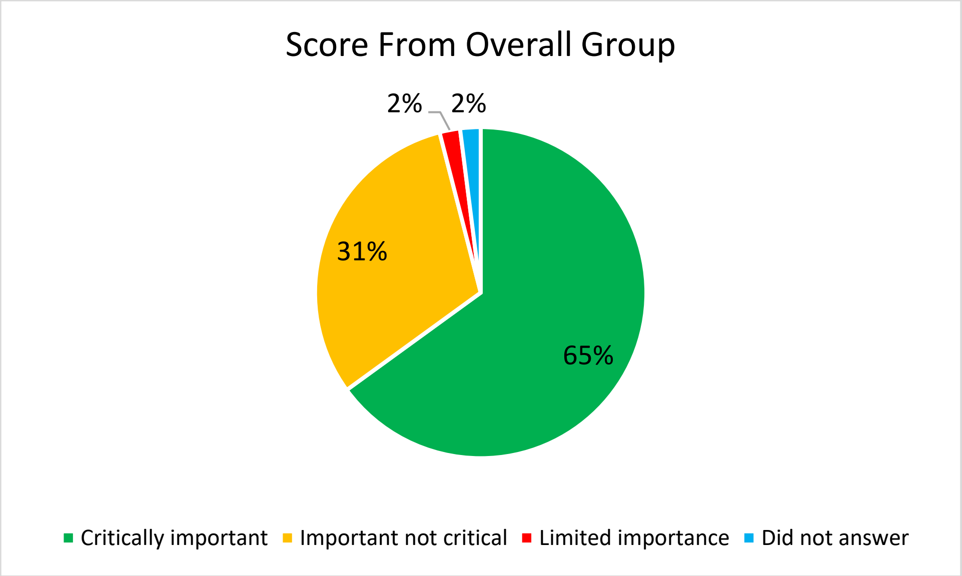

Scores From Each Stakeholder Group

Patients
Critically important: 50%
Important not critical: 37%
Limited importance: 0%
Did not answer: 13%

Researchers
Critically important: 75%
Important not critical: 29%
Limited importance: 6%

Clinicians
Critically important: 55%
Important not critical: 45%
Limited importance: 0%

Service-planners/Policymakers
Critically important: 71%
Important not critical: 29%
Limited importance: 0%

Self-efficacy

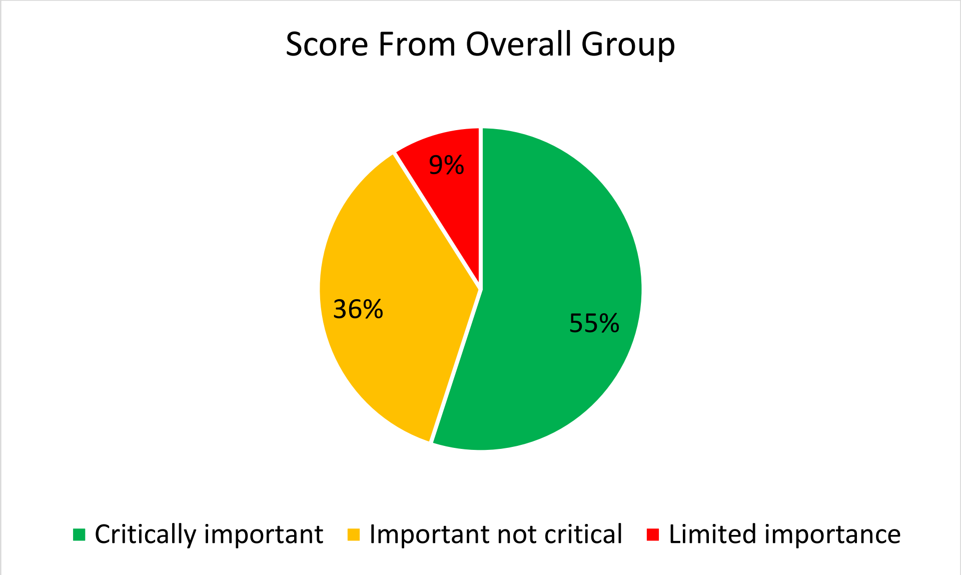

Scores From Each Stakeholder Group

Patients
Critically important: 75%
Important not critical: 25%
Limited importance: 0%

Researchers
Critically important: 37%
Important not critical: 44%
Limited importance: 19%

Clinicians
Critically important: 64%
Important not critical: 27%
Limited importance: 9%

Service-planners/Policymakers
Critically important: 57%
Important not critical: 43%
Limited importance: 0%

Reasons For Scores

Please note that the number in brackets shows the percentage of participants that gave that reason.

<u>Reasons for excluding:</u>
Not related to the primary goal of the intervention (2%).
Not all tasks are related to falls or fear of falling (2%).

Static balance

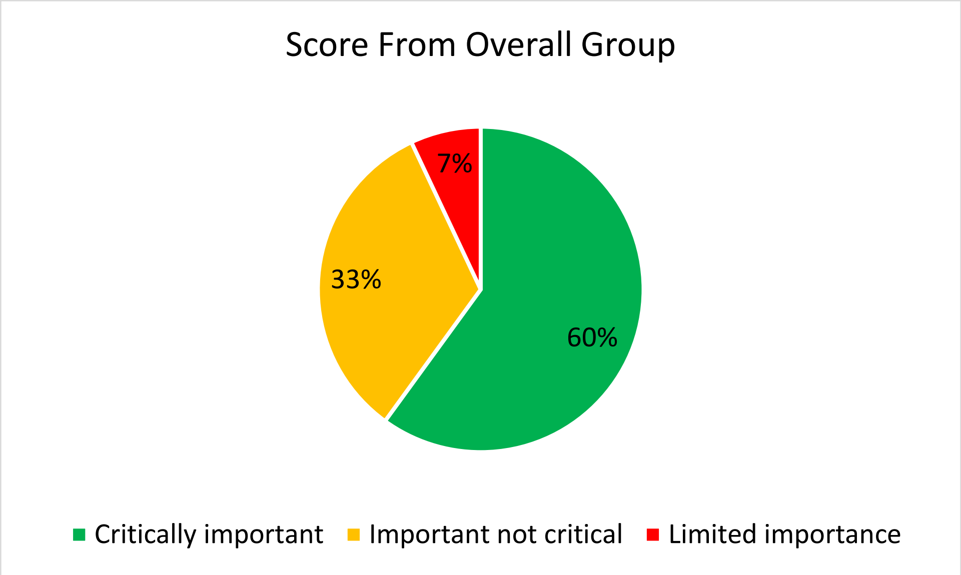

Scores From Each Stakeholder Group

Patients
Critically important: 100%
Important not critical: 0%
Limited importance: 0%

Researchers
Critically important: 25%
Important not critical: 56%
Limited importance: 19%

Clinicians
Critically important: 55%
Important not critical: 45%
Limited importance: 0%

Service-planners/Policymakers
Critically important: 100%
Important not critical: 0%
Limited importance: 0%

Reasons For Scores

Please note that the number in brackets shows the percentage of participants that gave that reason.

<u>Reasons for including:</u>
Static balance ability is foundational to dynamic balance ability (2%).
Important for falls prevention (2%).

<u>Reasons for excluding:</u>
Falls occur during dynamic activities and so static balance should not be the focus of a falls prevention intervention (2%).
Not as important as proactive/anticipatory and reactive balance (2%).
Static balance is not an indicator of overall balance (2%).

Time to first post-intervention fall

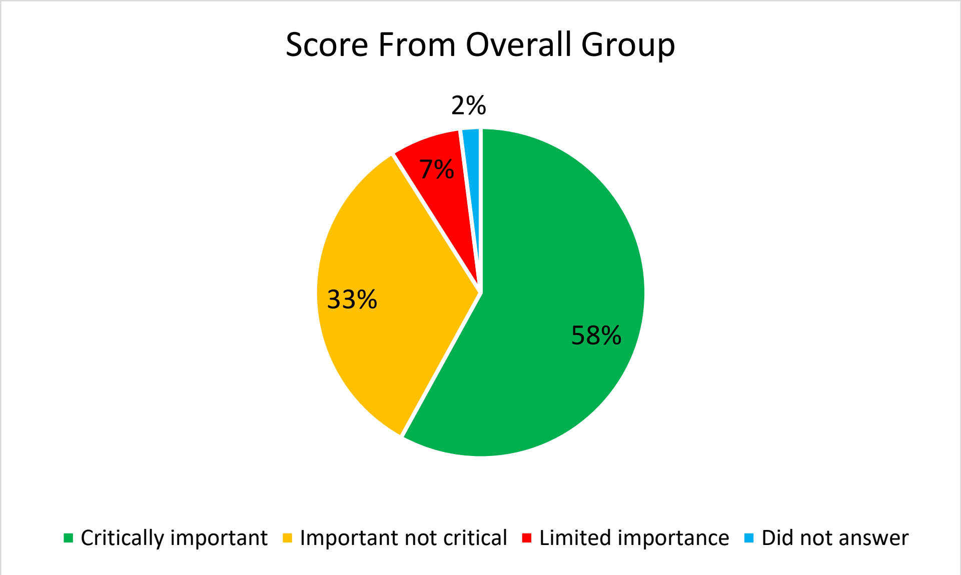

Scores From Each Stakeholder Group

Patients
Critically important: 50%
Important not critical: 25%
Limited importance: 13%
Did not answer: 12%

Researchers
Critically important: 63%
Important not critical: 31%
Limited importance: 6%

Clinicians
Critically important: 36%
Important not critical: 55%
Limited importance: 9%

Service-planners/Policymakers
Critically important: 86%
Important not critical: 14%
Limited importance: 0%

Reasons For Scores

Please note that the number in brackets shows the percentage of participants that gave that reason.

Reasons for including:
Provides information regarding how long treatment effects last (2%).

Falls rate adjusted for activity exposure

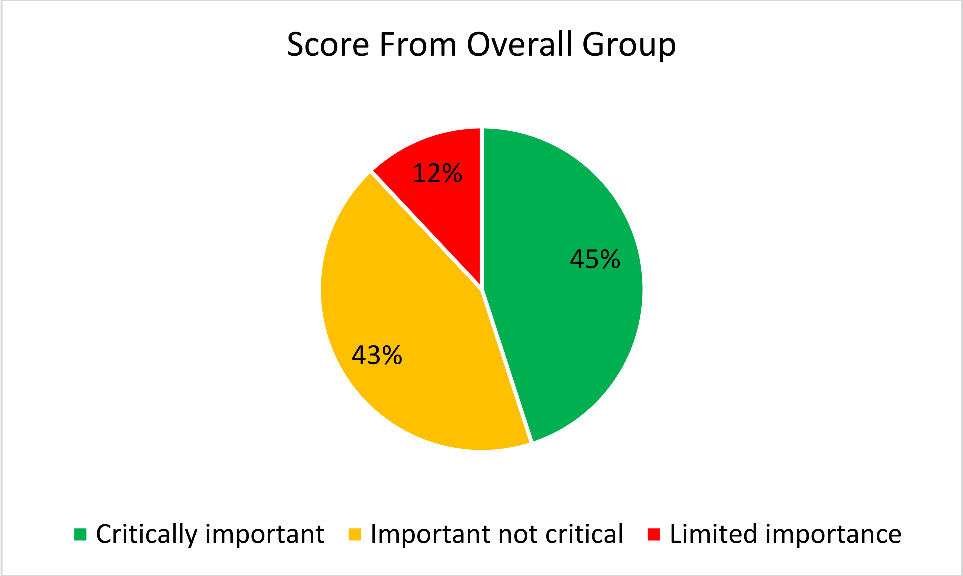

Scores From Each Stakeholder Group

Patients
Critically important: 74%
Important not critical: 13%
Limited importance: 13%

Researchers
Critically important: 50%
Important not critical: 38%
Limited importance: 12%

Clinicians
Critically important: 27%
Important not critical: 64%
Limited importance: 9%

Service-planners/Policymakers
Critically important: 29%
Important not critical: 57%
Limited importance: 14%

Reasons For Scores

Please note that the number in brackets shows the percentage of participants that gave that reason.

Reasons for excluding:
Unreliable measure due to the number of variables included in the outcome (4%).
Reliant on patient recall (2%).
