# Supplementary material for: A core outcome set for evaluating the effectiveness of mixed-diagnosis falls prevention interventions for people with Multiple Sclerosis, Parkinson’s Disease and stroke
Source: PLoS One. 2023 Nov 13;18(11):e0294193. doi: 10.1371/journal.pone.0294193 (PMC10642845; doi:10.1371/journal.pone.0294193)
Supplement: S6 Appendix — (PDF) [file pone.0294193.s006.pdf]

Appendix 6: Summary of the categorisation of outcomes following completion of the Delphi survey rounds.

Outcomes	Limited importance	Important not critical	Critically important
**Ability to engage in social activities**	5%	46%	49%
**Ability to independently perform activities of daily living**	12%	33%	55%
**Activity curtailment due to fear of falling**	0%	9%	91%
**Anxiety**	21%	40%	39%
**Balance confidence**	0%	28%	72%
**Bone density**	35%	46%	19%
**Bradykinesia**	30%	47%	23%
**Cadence**	28%	56%	16%
**Changes to home/work environment**	9%	51%	40%
**Cognition**	16%	21%	63%
**Cost-effectiveness**	5%	33%	62%
**Depression**	23%	51%	26%
**Disease impact**	14%	40%	46%
**Disease severity**	37%	28%	35%
**Dizziness**	19%	49%	32%
**Dual-tasking ability**	0%	53%	47%
**Dynamic balance**	0%	12%	88%
**Endurance**	12%	58%	30%
**Falls rate**	0%	21%	79%
**Falls rate adjusted for activity exposure**	12%	43%	45%
**Falls risk**	9%	31%	60%
**Falls self-efficacy**	0%	26%	74%
**Falls self-management skills**	0%	19%	81%
**Fatigue impact**	12%	46%	42%
**Fatigue severity**	21%	42%	37%
**Fear of falling**	0%	12%	88%
**Fitness**	19%	63%	18%
**Flexibility**	33%	53%	14%
**Freezing of gait**	16%	37%	47%
**Impact on carer**	21%	56%	23%
**Joining a support/community group**	36%	37%	25%
**Knowledge of how to fall**	16%	49%	35%

**Knowledge of how to get up from the floor after a fall**	17%	17%	66%
**Level of physical activity**	5%	31%	64%
**Lower limb strength**	10%	21%	69%
**Number of fallers**	5%	33%	62%
**Number of fall-related fractures**	7%	19%	74%
**Number of falls resulting in a long lie**	2%	33%	65%
**Number of falls resulting in healthcare utilisation**	7%	21%	72%
**Number of injurious falls**	2%	14%	84%
**Number of near falls**	19%	42%	39%
**Number of recurrent fallers**	2%	31%	65%
**Objectively assessed ability to perform activities of daily living**	10%	21%	69%
**Objectively assessed mobility**	5%	24%	71%
**Pain**	30%	42%	28%
**Peer-support**	28%	42%	30%
**Perceived control of falls**	14%	41%	45%
**Quality of life**	5%	25%	70%
**Self-efficacy**	9%	36%	55%
**Self-perceived impact on carer/family**	21%	56%	23%
**Self-reported ability to perform activities of daily living**	5%	51%	44%
**Self-reported mobility**	12%	49%	39%
**Sleep quality**	37%	40%	23%
**Static balance**	7%	33%	60%
**Stride length**	26%	51%	23%
**Time spent out of bed during daytime**	21%	51%	28%
**Time to first post-intervention fall**	7%	33%	58%
**Total number of falls**	5%	9%	86%
**Understanding of personal falls risk factors**	2%	28%	70%
**Walking distance**	19%	44%	37%
**Walking self-efficacy**	7%	72%	21%
**Walking speed**	23%	35%	42%

Outcome selection results

Category A: Included in preliminary core outcome set (brought forward to consensus meeting)

Category B1: >50% critically important and <15% limited importance (brought forward to consensus meeting)

Category B2: ≤50% critically important or ≥15% limited importance (remain on supplementary outcome list)
