# Supplementary figures and images for: A core outcome set for evaluating the effectiveness of mixed-diagnosis falls prevention interventions for people with Multiple Sclerosis, Parkinson’s Disease and stroke
Source: PLoS One. 2023 Nov 13;18(11):e0294193. doi: 10.1371/journal.pone.0294193 (PMC10642845; doi:10.1371/journal.pone.0294193)

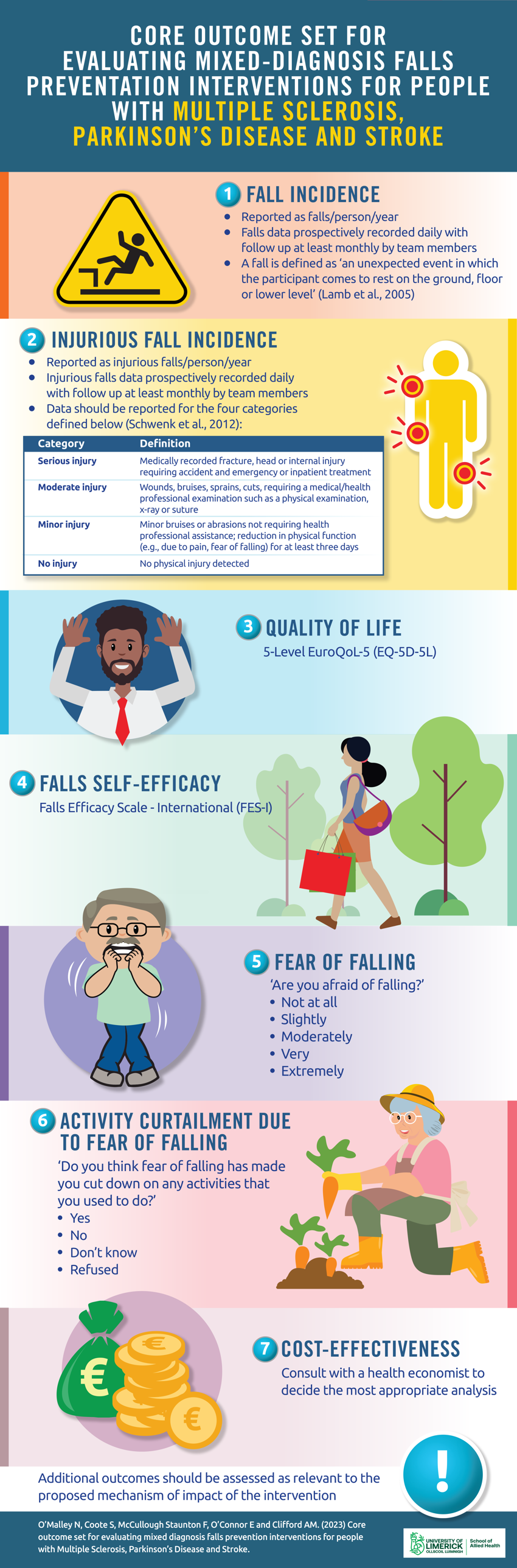

Supplement: S7 Appendix — (PNG) [file pone.0294193.s007.png]
